# Are we still too late to preserve the testes? A global survey of delayed consultation and risk factors for testicular torsion: a systematic review and meta-analysis

**DOI:** 10.3389/frph.2026.1735652

**Published:** 2026-02-24

**Authors:** Miao Sun, Chengjun Yu, Zhongyao Zeng, Yuanzhi Song, Fengming Ji, Yang Liu, Shiyu Peng, Bojingjia Liu, Runchang Wang, Shengde Wu

**Affiliations:** 1 Department of Urology Children's Hospital of Chongqing Medical University, National Clinical Research Center for Child Health and Disorders, Ministry of Education Key Laboratory of Child Development and Disorders, Chongqing, China; 2 Department of Pediatric Urology, Chongqing Key Laboratory of Structural Birth Defect and Reconstruction, Chongqing, China; 3 Department of Pediatric Urology, National Clinical Research Center for Child Health and Disorders, Chongqing, China; 4 Department of Pediatric Urology, China International Science and Technology Cooperation Base of Child Development and Critical Disorders, Chongqing, China; 5 Department of Pediatric Urology, Chongqing Key Laboratory of Pediatrics Chongqing, Chongqing, China; 6 Department of Pediatric Urology, Ministry of Education Key Laboratory of Child Development and Disorders, Chongqing, China

**Keywords:** delayed consultation, global epidemiology, meta-analysis, orchiectomy, symptom duration, testicular torsion

## Abstract

**Background:**

Testicular torsion (TT) is a urological emergency that requires prompt diagnosis and urgent surgical intervention. Delayed presentation is strongly associated with testicular loss and long-term atrophy.

**Objective:**

To systematically assess global trends in delayed consultation and mean symptom duration (MSD) in TT and to identify associated risk factors.

**Methods:**

A systematic review and meta-analysis of studies (1970–2025) that reported delayed consultation rates, MSD, orchiectomy rates, misdiagnosis, and patient transfers (PROSPERO: CRD420251155132).

**Results:**

A total of 176 studies from 45 countries (100,166 cases) were included in this study, of which 15 (5,221 cases) analyzed delayed consultation and 14 (1,513 cases) analyzed MSD. The consultation rate within 6 h ranged from 14.29% to 72.58%, whereas MSD ranged from 4.35 to 107.45 h. Pooled risk ratios (RRs) indicated that abdominal pain reduced the risk of delayed for >6 h [RR 0.91, 95% CI 0.68–1.21] but increased the risk for >12 h [1.19, 1.04–1.37] and >24 h [1.05, 0.77–1.43], while hydrocele decreased [>12 h [0.69, 0.47–1.02], >24 h [0.56, 0.34–0.92]]. Misdiagnosis [>12 h [1.52, 1.27–1.83], >24 h [1.10, 0.63–1.92]] and first visit to primary or secondary care unit [>12 h [1.29, 0.96–1.74], >24 h [1.36, 0.98–1.91]] significantly increased the risk. Transfer was protective and associated with lower odds of prolonged delays [>6 h [0.74, 0.50–1.08], >24 h [0.63, 0.44–0.90]]. A comparative meta-analysis of MSD demonstrated longer durations during the pandemic (SMD −0.37; 95% CI: −0.59, −0.14) in patients without manual detorsion (−0.70; −1.03, −0.37) and in patients misdiagnosed (2.36; 0.34, 4.38). Transfer from other hospitals was associated with shorter durations (−0.42; −0.60, −0.23).

**Conclusions:**

Delayed presentation remains widespread with notable regional disparities. Symptoms, healthcare pathways, misdiagnosis, and public health crises affect timely care. Improved awareness, optimized referral pathways, and strengthened emergency access are essential to minimize testicular loss.

**Systematic Review Registration:**

identifier CRD420251155132.

## 1 Introduction

Testicular torsion (TT) is a urological emergency that requires immediate diagnosis and surgical intervention to preserve testicular viability (1), which declines sharply after 6 h, with irreversible infarction typically occurring within 24 h (2, 3). Mean symptom duration (MSD) reflects testicular ischemia time, and prolonged ischemia leads to germ-cell apoptosis, seminiferous tubule necrosis, and microvascular injury, and also increases susceptibility to reperfusion injury and postoperative atrophy after detorsion. Delayed consultation and treatment are widely reported and are strongly associated with poor outcomes, including testicular loss, long-term atrophy, and hormonal dysfunction (4–8), highlighting the critical importance of timely presentation and management.

Although consultation delays and orchiectomy rates have been reported across regions, the overall global epidemiologic pattern remains unknown (2). Existing studies have reported highly variable timely consultation rates, symptom duration, and salvage outcomes across countries, but these data are fragmented, predominantly single-center, and limited by inconsistent definitions and small sample sizes. Moreover, to our knowledge, no prior meta-analysis or systematic review has comprehensively examined delayed presentation in TT at a global level, its global distribution, or the clinical and healthcare-system factors contributing to it. Global comparisons of MSD have not been synthesized, and the influence of misdiagnosis, interfacility transfer, and the COVID-19 pandemic on consultation delay remains unclear.

To fill these knowledge gaps, in this study, we conducted a global systematic review and meta-analysis that, for the first time (1) compares timely consultation rates and MSD, rates of misdiagnosed scrotum and orchiectomy across countries and regions; (2) quantifies pooled risk estimates for multiple clinical and healthcare-system factors; and (3) assesses the possible influences of misdiagnosis, interfacility transfer, and the COVID-19 pandemic on consultation behavior. This study provides a broad global perspective on delays in testicular torsion presentation and highlights opportunities to enhance early recognition, streamline referral pathways, and improve access to care to reduce preventable testicular loss.

## 2 Method

This systematic review and meta-analysis adhered to the Preferred Reporting Items for Systematic Reviews and Meta-Analyses (PRISMA) guidelines (9) and the Meta Analyses of Observational Studies in Epidemiology (MOOSE) guidelines (10) and was prospectively registered with PROSPERO (CRD420251155132).

### 2.1 Search strategy and eligibility criteria

A comprehensive search was independently performed by two reviewers across multiple databases between 1 January 1970 and 30 September 2025, including PubMed, Google Scholar Web of Science, Embase, Cochrane Library, Chinese Biomedical Literature Database (CBM), China National Knowledge Infrastructure (CNKI), WanFang Data, African Journals Online (AJOL), and eLibrary.RU. Boolean operators and MeSH terms were applied using keywords such as “delayed diagnosis,” “treatment delay,” and “spermatic cord torsion.” The detailed search strategy is presented in Supplementary Table S1.

**Figure 1:**
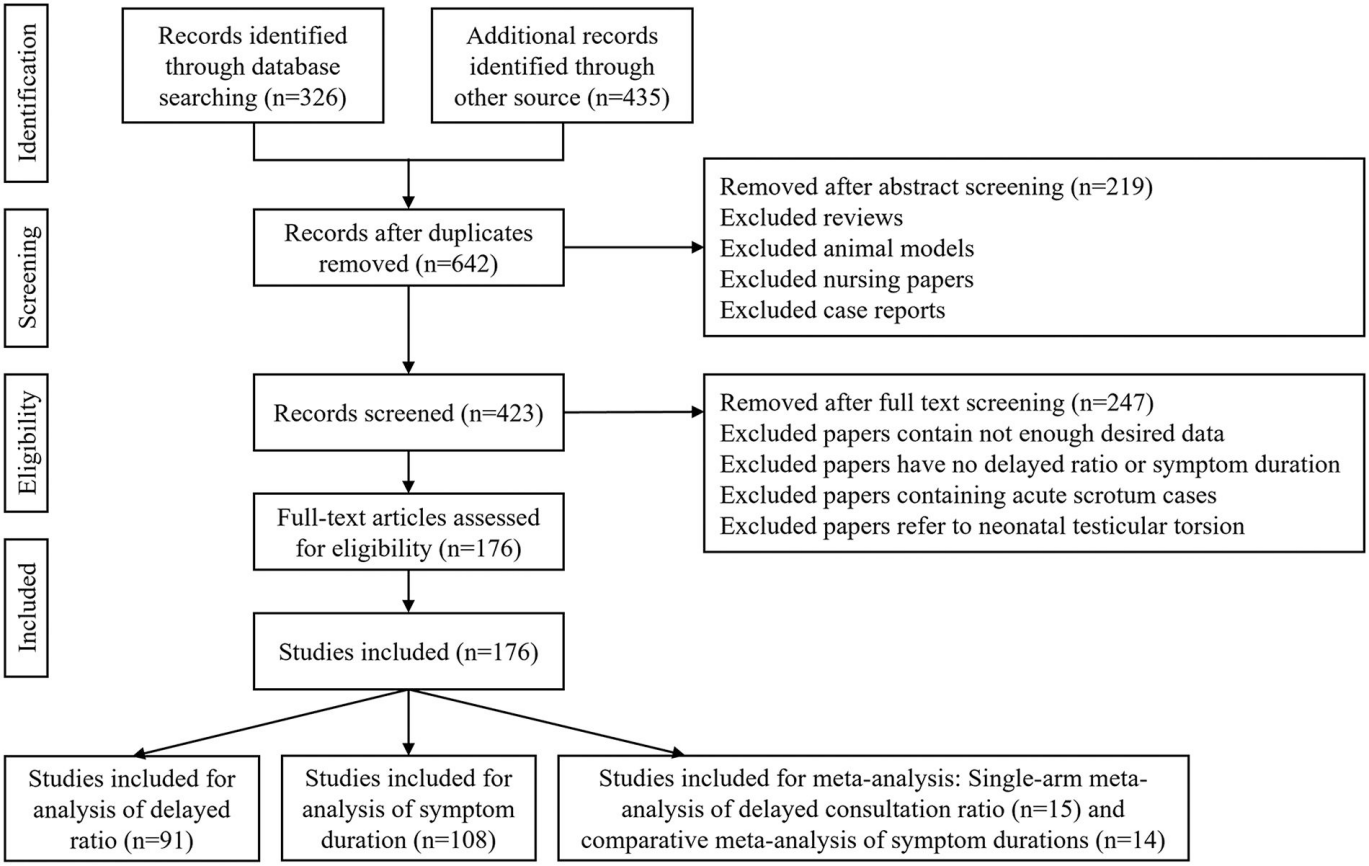
Flow diagram of identification and eligibility of publications.

Eligibility criteria for included studies were summarized using the P–E–C–O–S framework (Supplementary Table S2). Potential determinants of delayed consultation and mean symptom duration (MSD) were categorized into five domains: symptoms, first-consultant factors, interfacility transfer, health insurance status, and the COVID-19 pandemic. These exposures were analyzed for their association with outcomes in included studies. The process of identifying eligible studies and reasons for exclusion are presented in Figure 1.

### 2.2 Data extraction and quality assessment

Data extracted from eligible studies included first author, publication year, study period, study type, sample size, region, age, timely visits, orchiectomy rate, duration of symptoms, and factors and outcome measurement (Supplementary Tables S2, S3, Table 2). All data were extracted and quality was independently assessed by two authors, and discrepancies were resolved by discussion or consultation with a third author. The modified Newcastle–Ottawa Scale (mNOS) was used for quality assessment of cohort and case-control studies, and Joanna Briggs Institute (JBI) Critical Appraisal Tools were used for other types include case series, cross-sectional study, non-randomized intervention studies and diagnostic accuracy study (11, 12) (Supplementary Tables S2, S3, Table 2).

### 2.3 Definition

Symptom duration was defined as time between symptom's onset and surgery. Misdiagnosis referred to instances in which testicular torsion was not recognized at first evaluation, causing discharge by a primary care physician in a community, outpatient, or emergency setting. Factors contributing to delayed consultation were symptoms (nausea or vomiting, fever, abdominal pain, and hydrocele), first consultation information (misdiagnosis, level of initial healthcare facility, and preoperative ultrasonography), transfer, insurance, and pandemic period.

### 2.4 Data analysis

To compare testicular torsion outcomes such as timely consultation, orchiectomy, and symptom duration across countries, nationwide databases were prioritized. In cases where nationwide data were not available, relevant studies from the same country were aggregated to calculate country-specific mean estimates. Heat maps were generated using Tableau, and regional comparisons were visualized using Microsoft Excel.

The distribution of included studies was summarized by publication year, region, and data source. A meta-analysis of proportions and delayed consultation risk was conducted using the *meta* package in R (version 2024.12.1). Pooled prevalence and relative risks (RRs) were estimated using the Freeman–Tukey double arcsine transformation of the original proportions. Binomial confidence intervals (CIs) were obtained through the Clopper–Pearson method. Because of the small number of included studies, the reported RR values and the corresponding 95% CI were directly extracted for pooled analysis. If the original study reported only the odds ratio (OR) or rate ratio (HR), it was considered equivalent to the RR and included in the combined analysis. If neither measure was reported, the RR was calculated directly from the available case counts.

Continuous variables were compared using the Student's *t*-test or non-parametric tests (Wilcoxon or Mann–Whitney *U* tests), and categorical variables were evaluated by using chi-square or Fisher's exact tests. The symptom duration of different exposure factor groups was analyzed using R language, and the weighted mean difference was recorded as the summary statistic.

Heterogeneity was assessed using the *I*^2^ statistic and Cochran's *Q* test. *I*² values were interpreted as low (<25%), moderate (25%–50%), or high (50%–75%) (13). A *p*-value of < 0.10 for the *Q* test indicated significant heterogeneity. Leave-one-out sensitivity analyses were performed to assess the robustness of the pooled results by sequentially omitting each study (14). Because of the small number of studies included in each analysis (<5), funnel plots and Egger's test were considered unreliable for assessing publication bias (15). A value of *p* < 0.05 was considered statistically significant.

## 3 Results

### 3.1 National trends

#### 3.1.1 Timely consultation rates

**Figure 2:**
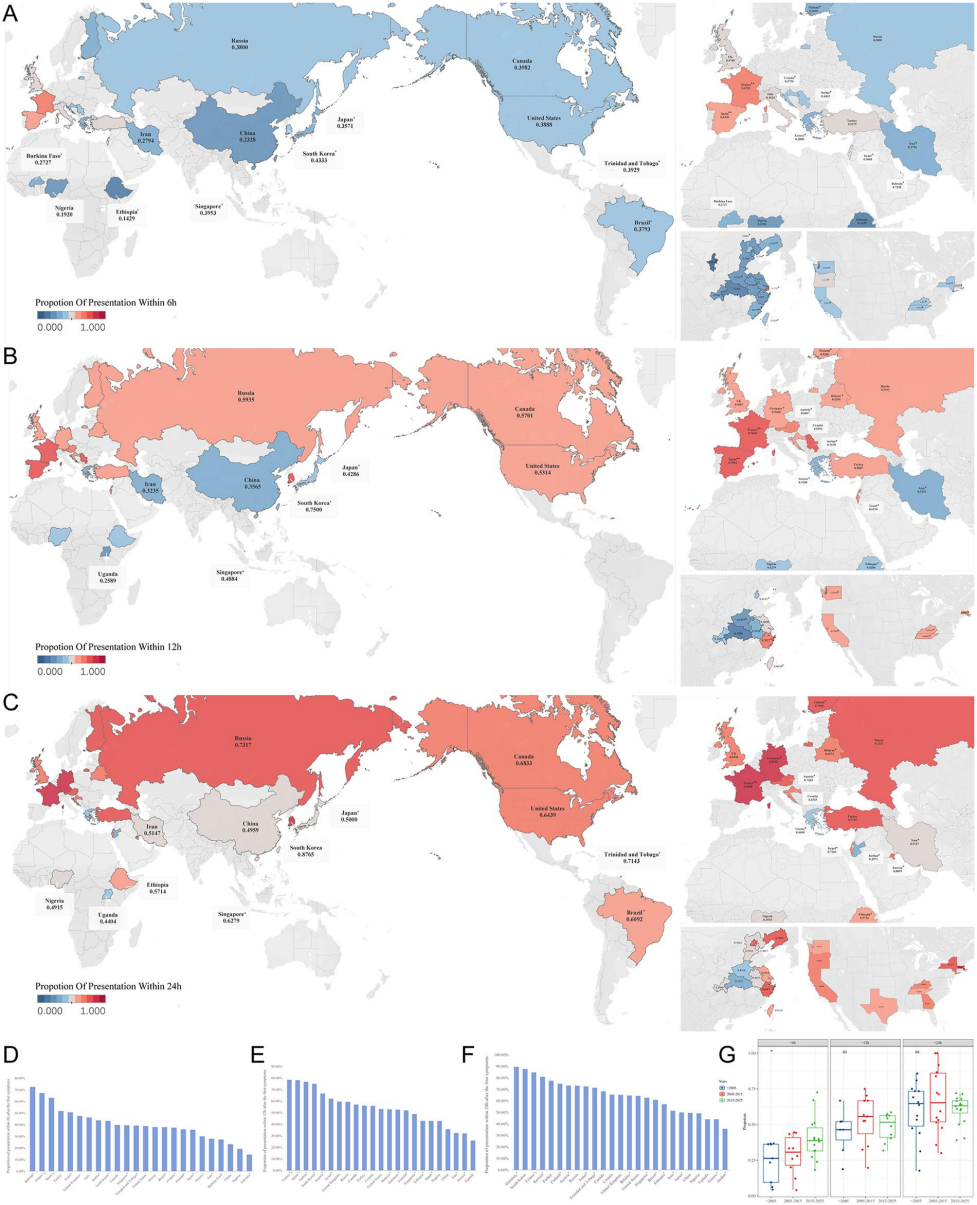
Proportion of timely medical visit for testicular torsion in an international comparison. Heat map of proportion of medical visit **(A)** within 6 h **(B)** within 12 h **(C)** within 24 h. International comparison of visit rates **(D)** within 6 h; **(E)** within 12 h; **(F)** within 24 h. **(G)** Change in the proportion of timely medical visit in different years. Countries or regions marked with a single asterisk (*) represent data from a single-study at a regional level. Countries marked with a double asterisk (**) represent data from a single-study at a national level. All other data are pooled estimates from multiple studies.

Among countries that reported delayed consultation rates for TT, only five countries reported consultation rates above 50% within 6 h (16–23) (Figures 2A,D). More than half of the countries had a 12 or 24 h consultation rate of over 50%, and the consultation rates were generally higher in Europe, America, and South Korea than in other Asian and African countries (Figures 2A–F).

A temporal analysis showed that the overall consultation rate within 6 h had increased over time, whereas consultation rates within 12 and 24 h did not change significantly (Figure 2G).

Substantial intranational variation was observed in China, with the eastern coastal areas reporting higher variation and the western inland areas reporting lower variation. Shanghai's consultation rate (77.27%) within 6 h was significantly higher than that in other provinces (24), while Zhejiang Province had the highest rates within 12 h (70.27%) (25) and within 24 h (80.87%) (25–27). The timely consultation rates reported by different states in the United States (US) were similar, and most were close to the levels reported using the national database (Figures 2A–C).

Overall, higher proportions of timely presentation were observed in economically developed regions.

#### 3.1.2 Mean symptom durations

**Figure 3:**
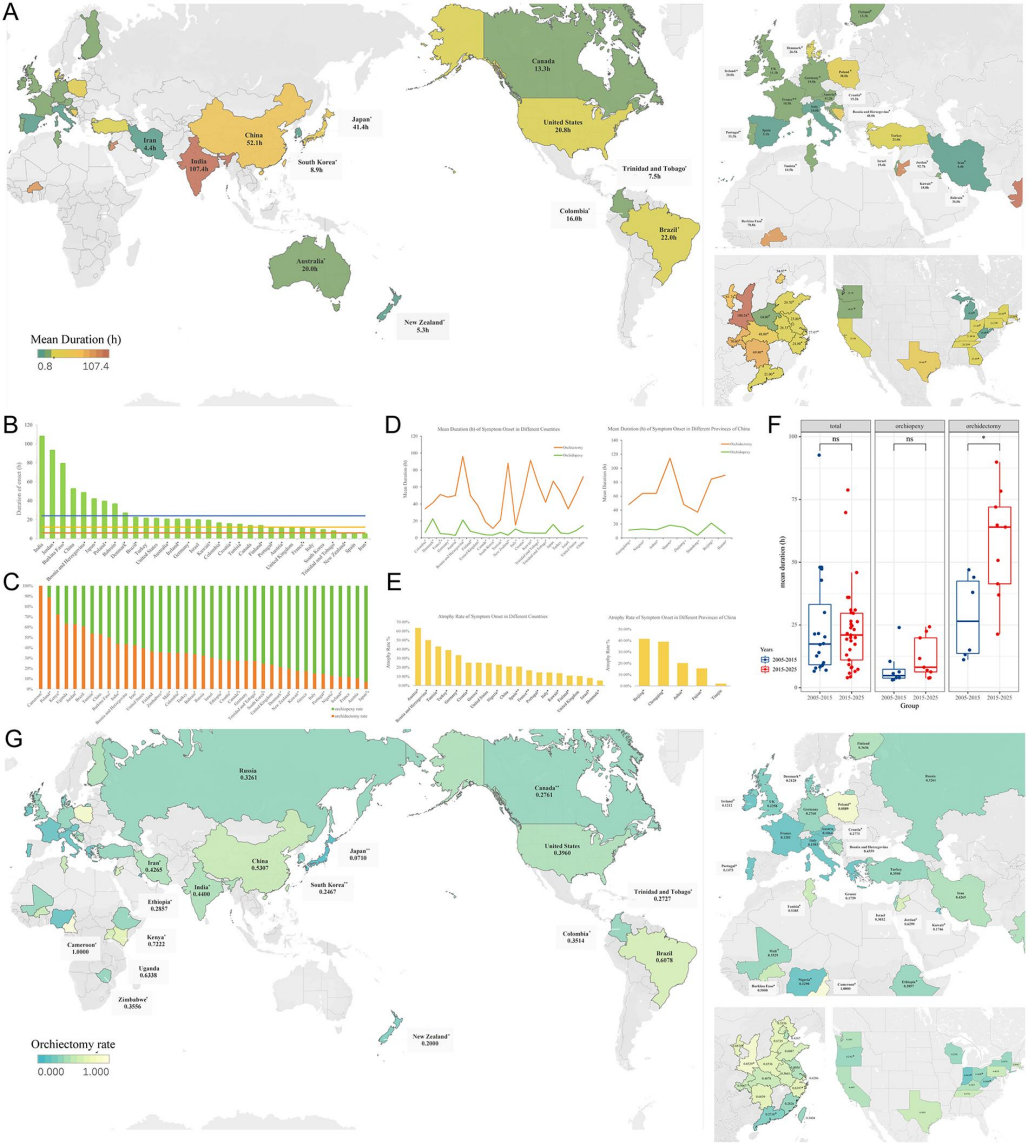
Mean symptom durations (h) and orchiectomy rate for testicular torsion in an international comparison. **(A)** Heat map of mean symptom durations. **(B)** International comparison of mean symptom durations. **(C)** International comparison of orchiectomy and orchiopexy rate. **(D)** Comparison of atrophy rate in different countries and in different provinces of China. **(E)** Comparison of mean symptom duration in patients undergoing orchiopexy and orchiectomy in different countries and in different provinces of China. **(F)** Change in the mean symptom durations in different years. **(G)** Heat map of orchiectomy rate. Countries or regions marked with a single asterisk (*) represent data from a single-study at a regional level. Countries marked with a double asterisk (**) represent data from a single-study at a national level. All other data are pooled estimates from multiple studies.

Among the countries that reported the MSD of TT, only three countries reported an average symptom duration of less than 6 h. Bosnia and Herzegovina, South Korea, Italy, France, the United Kingdom (UK), Australia, and Portugal had an MSD of less than 12 h (Figures 3A,B). India reported the longest MSD (107.4 h) (28, 29).

In China, all provinces reported MSD values exceeding 12 h, with Henan Province reporting the shortest duration and Shanxi Province the longest duration (30, 31). West Virginia and Michigan were the only two states in the United States to report an MSD of less than 12 h (32, 33).

Across regions, a prolonged MSD was commonly observed. The orchiectomy group had a significantly longer MSD than the orchiopexy group (Figure 3D), and the orchiectomy group also had a significantly prolonged MSD in the past decade (Figure 3F).

#### 3.1.3 Orchiectomy rate and atrophy rate

Orchiectomy rates varied widely across countries. The highest orchiectomy rates were reported in Cameroon, Poland, and Kenya (34–36), whereas the lowest rates were observed in Japan, Austria, and France (17, 37–42) (Figures 3C,G). Overall, the geographic distribution of orchiectomy rates largely paralleled that of delayed consultation, with lower rates in economically developed regions.

Postoperative testicular atrophy rates showed a different distribution. Austria reported the highest atrophy rate, whereas Denmark reported the lowest (40, 43). Among China's provinces, Beijing reported the highest atrophy rate, while Tianjin reported the lowest (44, 45) (Figure 3E). Unlike orchiectomy rates, atrophy rates did not consistently align with delayed consultation patterns.

Across countries, similar global patterns emerged. Timely consultation within 6 h was rare, whereas presentation within 12–24 h was more frequent. The MSD exceeded 12 h in most regions, and higher orchiectomy rates were observed in settings with longer duration and lower timely consultation.

### 3.2 Risk factors of delayed consultation for testicular torsion

#### 3.2.1 Characteristics of the included studies

Following screening and data extraction, 15 studies met the inclusion criteria (3, 6, 35, 46–57). All included studies reported the prevalence of factors of delayed consultation odds ratio (Supplementary Table S3). A total of five studies evaluated two exposure categories (3, 6, 48, 52, 55) and one evaluated three exposure categories (54) (Table 1, Supplementary Table S3). The overall methodological quality was high, with the mean (SD) NOS score being 7.8 (0.86).

**Table 1:** Distribution of studies included for the meta-analysis of delayed medical consultations.

Classification	Total	Symptom		First consultant		Transfer		Insurance		Pandemic	
	*N* = 15	*N* = 8	Place of data origin	*N* = 5	Place of data origin	*N* = 3	Place of data origin	*N* = 2	Place of data origin	*N* = 3	Place of data origin
Year of publication											
2015–2020	4	4	Poland, US	-	-	1	US	-	-	-	-
2020–2025	11	4	China, France, and Serbia	5	Serbia, China, US, France, and Bosnia and Herzegovina	2	US	2	China and US	3	Croatia and US
Region											
Asia-Pacific	2	2	China	2	China	-	-	1	China	-	-
Eastern Europe	4	2	Serbia, Poland	2	Serbia and Bosnia and Herzegovina	-	-	-	-	1	Croatia
Western Europe	1	1	France	1	France	-	-	-	-	-	-
North America	8	3	US	1	US	3	US	1	US	2	US
Source of data											
Self-report or interview	1	-	-	-	-	1	US	-	-	-	-
Register database	15	8	Serbia, China, US, France, and Poland	5	China, France, US, Serbia, and Bosnia and Herzegovina	3	US	2	China, US	3	Croatia, US

Symptom: nausea or vomiting, fever, abdominal pain, hydrocele; first consultant: misdiagnosis, first-consultant medical institution, preoperative ultrasound, manual detorsion.

Overall, most studies were published between 2020 and 2025, accounting for 73.33% of the included literature (Table 1). Geographically, studies originated primarily from North America (53.33%), followed by Eastern Europe (26.67%), Asia-Pacific (13.33%), and Western Europe (6.67%). All studies were conducted in middle- or high-income settings; with none from low-income countries. Most relied on registry-based data, and only one study utilized interview or self-reported information (Table 1).

#### 3.2.2 Distribution of factors associated with delayed medical consultation

**Figure 4:**
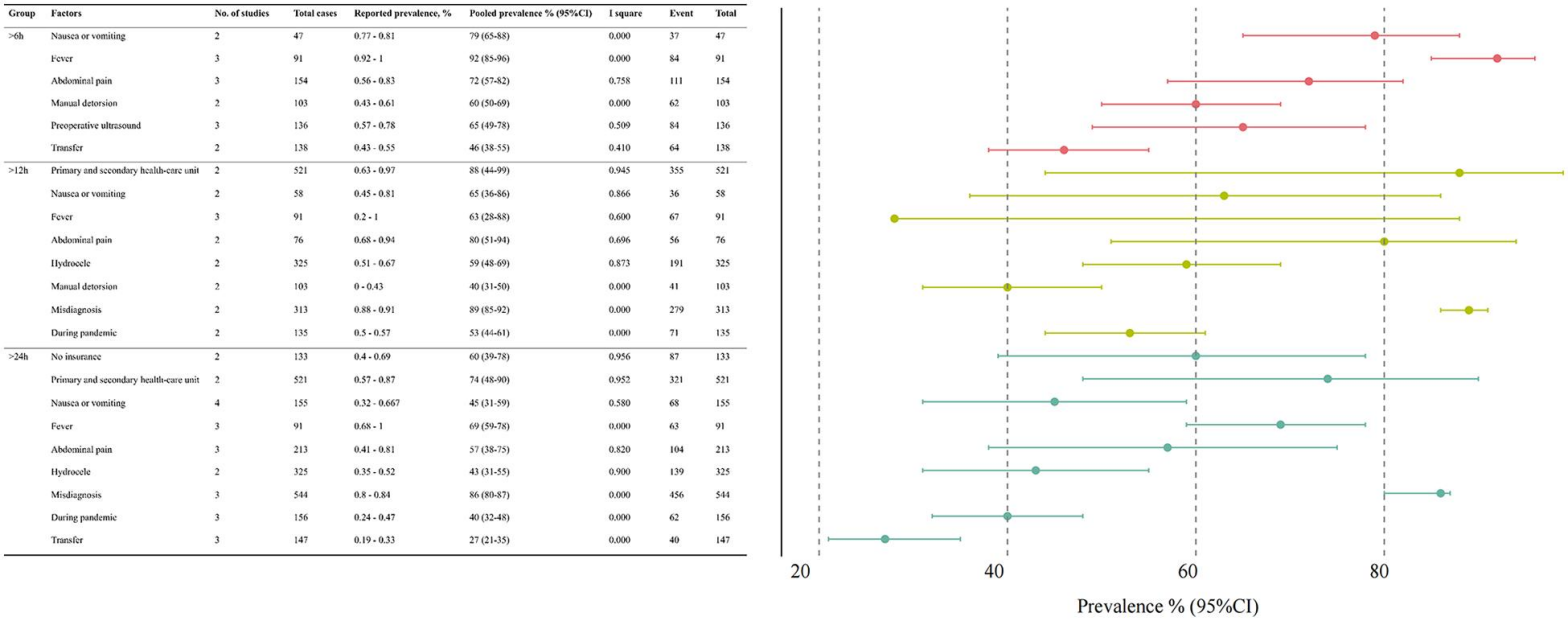
Distribution of factors associated with delayed medical consultation in patients with testicular torsion.

Exposure prevalence was pooled through a meta-analysis (Figure 4, detailed forest plots shown in Supplementary Figures S1–S5).

Among TT patients who presented over 6 h, gastrointestinal or systemic symptoms were common: nausea or vomiting (79%), fever (92%), and abdominal pain (72%). Preoperative interventions included manual detorsion (60%) and ultrasound (65%), and 46% of patients were transferred. First-consultant factors included preoperative manual detorsion (60%), preoperative ultrasound (65%), and interfacility transfer (46%).

Among patients who presented over 12 h, nausea or vomiting (65%), fever (63%), abdominal pain (80%), and hydrocele (59%) were frequently reported. Care pathway–related characteristics included initial presentation to primary or secondary healthcare facilities (88%), misdiagnosis (89%), and presentation during the pandemic period (53%).

Among patients who presented over 24 h, nausea or vomiting (45%), fever (69%), abdominal pain (57%), and hydrocele (43%) remained prevalent. At the first visit, 74% sought care in primary and secondary healthcare units and 86% were misdiagnosed. Pandemic-period presentation (40%), lack of insurance (60%), and transfer between facilities (27%) were also observed.

Overall, symptom-related and healthcare pathway factors were consistently prevalent among patients with delayed presentation. A high heterogeneity observed in the pooled analyses (*I*^2^ > 75%) for abdominal pain, hydrocele, misdiagnosis, and first presentation to primary/secondary healthcare units likely reflected variations in symptom reporting, healthcare pathways, and regional healthcare system structures, as well as the limited number of studies or cases included per factor.

#### 3.2.3 Associated risk for delayed medical consultation

**Figure 5:**
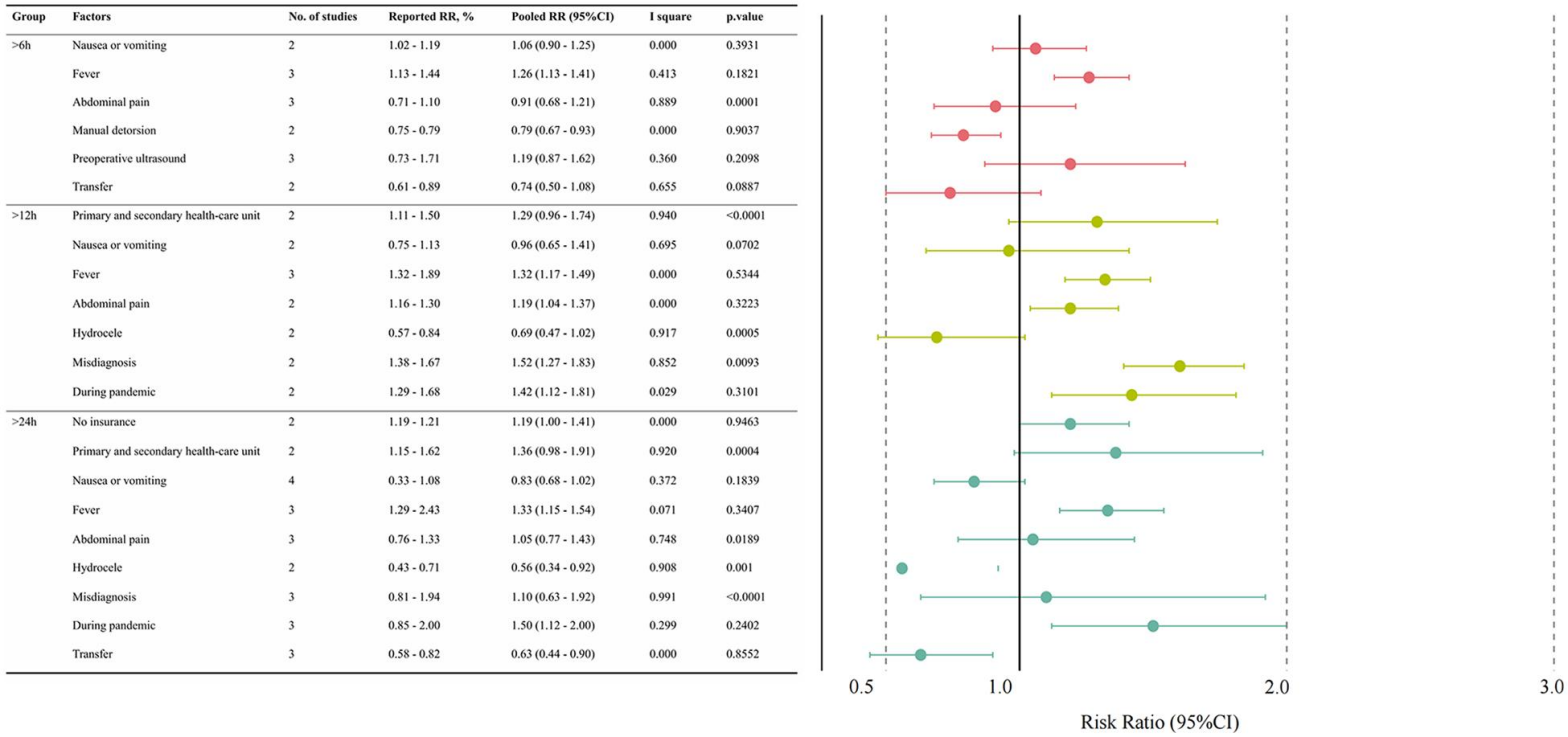
Pooled risk ratios (95% CI) for factors associated with delayed medical consultation in patients with testicular torsion.

Pooled RR analyses associated with delayed consultation are summarized in Figure 5, with detailed forest plots shown in Supplementary Figures S6–S10.

Among symptom-related factors, fever was consistently associated with increased risk (>6 h [pooled RR (95% CI): 1.26 (1.13–1.41)], >12 h [pooled RR (95% CI): 1.32 (1.17–1.49)], and >24 h [pooled RR (95% CI): 1.33 (1.15–1.54)]). Hydrocele was associated with a significantly decreased risk (>12 h [pooled RR (95% CI): 0.69 (0.47–1.02)] and >24 h [pooled RR (95% CI): 0.56 (0.34–0.92)]). Nausea and vomiting showed a modest increase in risk of delay over 6 h (>6 h [pooled RR (95% CI): 1.06 (0.90–1.25)]), but a reduced the risk of delays exceeding 12 h and 24 h (>12 h [pooled RR (95% CI): 0.96 (0.65–1.41)] and >24 h [pooled RR (95% CI): 0.83 (0.68–1.02)]). Abdominal pain significantly reduced the risk of delays over 6 h (>6 h [pooled RR (95% CI): 0.91 (0.68–1.21]) but increased the risk for delays exceeding 12 and 24 h (>12 h [pooled RR (95% CI): 1.19 (1.04–1.37)] and >24 h [pooled RR (95% CI): 1.05 (0.77–1.43)]).

Care pathway factors were also associated with delayed consultation. First consultation to primary and secondary healthcare units (>12 h [pooled RR (95% CI): 1.29 (0.96–1.74)] and >24 h [pooled RR (95% CI): 1.36 (0.98–1.91)]) and misdiagnosis (>12 h [pooled RR (95% CI): 1.52 (1.27 −1.83) and >24 h [pooled RR (95% CI): 1.10 (0.63–1.92)]) significantly increased the risk of delayed treatment, while preoperative transfer (>6 h [pooled RR (95% CI): 0.74 (0.50–1.08) and >24 h [pooled RR (95% CI): 0.63 (0.44–0.90)]) reduced the risk of delayed treatment. In addition, the risk of delaying treatment increased during the pandemic period (>12 h [pooled RR (95% CI): 1.42 (1.12–1.81)] and >24 h [pooled RR (95% CI): 1.50 (1.12–2.00)]).

Substantial heterogeneity (I² > 75%) was noted for several pooled RRs, including abdominal pain, hydrocele, misdiagnosis, and first presentation to primary or secondary healthcare units, and should be interpreted cautiously.

#### 3.2.4 Sensitivity analysis

No individual study significantly influenced the final pooled proportion estimates with the method of leave-one-out (Supplementary Figures S11, S12). Given the limited number of included studies, funnel plots and Egger's test were not applied to assess the publication bias.

### 3.3 Factors associated with mean symptom durations

#### 3.3.1 Study characteristics and quality assessment

A total of 14 studies (2015–2024) including 1,513 cases were included in this study. Overall methodological quality was high [mean (SD) NOS score 7.07(0.96)]. The study selection process is shown in Figure 1. Each study reported the MSD and at least one associated factor. The MSD was compared across different clinical contexts: pre- and postpandemic periods (three studies), patients with vs. without manual reduction (four studies), misdiagnosed vs. correctly diagnosed cases (two studies), and transferred vs. non-transferred patients (five studies) (Table 2, Supplementary Table S2).

**Table 2:** Summary of study characteristics and quality analysis of mean duration meta-analysis.

References	Country	Study Period	Group	Sample size		Age mean ± SD (year)			Study type	NOS
				Group 1	Group 2	Group 1	Group 2	*p*-value		
Lisa et al. (87)	US	2015–2019	Prepandemic vs. During Pandemic	79	38	13.7 ± 2.2	13.0 ± 1.5	0.0053	RC	8
Zenon et al. (50)	Croatia	2019–2020	Prepandemic vs. During Pandemic	68	51	14.3 ± 2.2	15.0 ± 1.5		RC	7
Sarah et al. (3)	US	2019–2020	Prepandemic vs. During Pandemic	137	84	13.2 ± 17.8	23.8 ± 31.5		RPC	7
Metin et al. (73)	Turkey	2015–2024	Manual detorsion vs. No manual detorsion	52	42	20.0 ± 6.0	26.0 ± 1.0	0.346	RCC	7
Qi et al. (88)	China	2017–2022	Manual detorsion vs. No manual detorsion	19	47	14.9 ± 2.1	15.0 ± 3.9		RCC	8
Tiziana et al. (89)	Italy	2020–2022	Manual detorsion vs. No manual detorsion	58	14	12.0 ± 2.1	11.7 ± 1.0		RCS	8
Sofia et al. (90)	Portugal	2014–2018	Manual detorsion vs. No manual detorsion	58	64	14.7 ± 5.2	11.5 ± 11.9		RC	8
Mao et al. (91)	China	2013–2021	Misdiagnosis vs. Confirm	27	46	5.5 ± 1.8	6.2 ± 3.5	0.0010	RC	6
Amihay et al. (92)	Israel	2008–2014	Misdiagnosis vs. Confirm	12	64	9.7 ± 8.1	12.7 ± 3.7		RCC	8
Emily et al. (46)	US	2018–2023	Transfer vs. No Transfer	96	37	-	-	0.32	RC	9
Lisa et al. (48)	US	2015–2020	Transfer vs. No Transfer	42	98	13.7 ± 1.3	14.1 ± 2.1		RC	8
Olivia et al. (55)	US	2016–2018	Transfer vs. No Transfer	32	9	-	-		RC	7
Janae et al. (93)	US	2011–2016	Transfer vs. No Transfer	36	89	13.1 ± 4.1	13.5 ± 3.6		RC	8
Puneeta et al. (75)	US	2005–2011	Transfer vs. No Transfer	35	79	-	-		RC	8

US, United States; RC, retrospective cohort study; RCC, retrospective case–control study; PC, prospective cohort study; RCS, retrospective case series; RPC, retrospective–prospective cohort study.

#### 3.3.2 Meta-analysis

**Figure 6:**
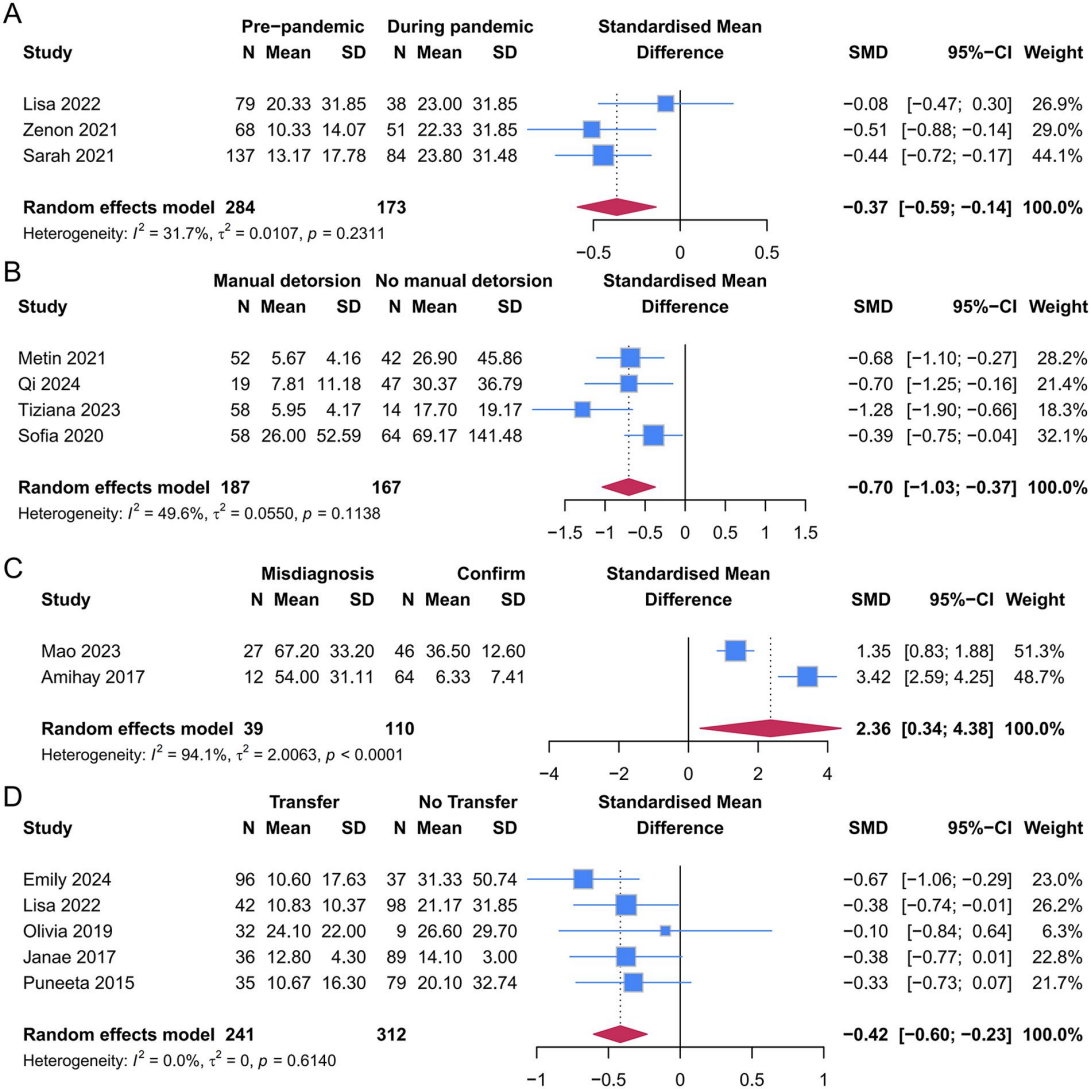
Forest plot comparisons of mean symptom durations between **(A)** pre-pandemic and during pandemic groups; **(B)** manual detorsion and no manual detorsion groups; **(C)** misdiagnosis and Confirm groups; **(D)** transfer and no transfer groups.

Patients who presented during the COVID-19 pandemic demonstrated a significantly longer MSD (SMD: −0.37, 95% CI: −0.59, −0.14, *p* = 0.002, *I*^2^ = 31.7%). The durations were significantly longer in patients without preoperative manual detorsion (SMD: −0.70, 95% CI: −1.03, −0.37, *p* < 0.0001, *I*^2^ = 49.6%). Initial misdiagnosis was associated with a longer MSD compared with accurate diagnosis (SMD: 2.36, 95% CI: 0.34, 4.38, *p* = 0.022). Heterogeneity was high for misdiagnosis (*I*^2^ = 94.1%), likely due to differences in misdiagnosis definitions and reporting across studies. Patients transferred from other hospitals experienced significantly shorter symptom durations than those who presented directly (SMD: −0.42, 95% CI: −0.60, −0.23, *p* < 0.0001; *I*² = 0.0%) (Figure 6A, Table 3).

**Table 3:** Results of factors of mean duration meta-analysis.

Factors (group)	Effect size (95% CI)	Z	*p*	*I*^2^%
Prepandemic vs. during pandemic	−0.37 (−0.59, −0.14)	−3.159	0.002	31.7
Manual detorsion vs. no manual detorsion	−0.70 (−1.03, −0.37)	−4.175	<0.0001	49.6
Misdiagnosis vs. confirm	2.36 (0.34, 4.38)	2.285	0.022	94.1
Transfer vs. no transfer	−0.42 (−0.60, −0.23)	−4.390	<0.0001	0.0

A sensitivity analysis using the leave-one-out method confirmed the stability of these results (Supplementary Figure S13).

#### 3.3.3 Trends in misdiagnosis and transfer rates

Among nine countries that reported misdiagnosis rates for TT, Brazil (58) reported the highest rate (68.32%), whereas Denmark (43) reported the lowest (3.66%). Higher misdiagnosis rates were observed in several middle- and low-income regions.

**Figure 7:**
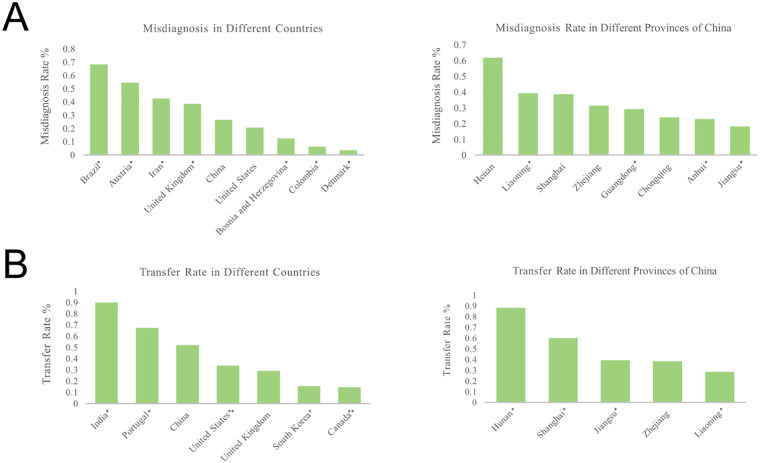
**(A)** Comparison of misdiagnosis rate in different countries and in different provinces of China. **(B)** Comparison of transfer rate in different countries and in different provinces of China. Countries or regions marked with a single asterisk (*) represent data from a single-study at a regional level. Countries marked with a double asterisk (**) represent data from a single-study at a national level. All other data are pooled estimates from multiple studies.

In China, Henan Province (61.76%) (59–61) reported the highest misdiagnosis rate, while Jiangsu Province (18.29%) (62) had the lowest. Transfer rates varied widely, with the highest rate reported in India (90.00%) (28) and the lowest in Canada (14.36%) (63). In China, studies from Hunan Province (88.24%) (64) reported the highest transfer rate, while Liaoning (28.57%) (65) reported the lowest (Figure 7). Because of limited reporting and single-study country/provincial data, these figures should be interpreted with caution.

## 4 Discussion

This systematic review and meta-analysis provides the first comprehensive global overview of delayed consultation and symptom duration in patients with TT. Our findings reveal substantial regional variation in timely consultation rates, MSD, and orchiectomy. Only a few countries in Europe and the Middle East reported consultation rates within 6 h exceeding 50%, while most Asian and African countries showed considerably longer delays. MSD and orchiectomy rates also showed marked geographic disparities, with developing regions showing prolonged presentation times and higher testicular loss rates. Overall, delayed presenters frequently exhibited systemic symptoms and followed similar care pathways characterized by initial low-tier healthcare contact, high misdiagnosis rates, substantial preoperative transfers, and increased delays during the pandemic.

Patients presenting with extrascrotal symptoms are more likely to be misdiagnosed, resulting in delayed diagnosis and treatment of TT (3). Abdominal pain, reported in 5%–22% of early TT cases (57, 66–72), is common but frequently overlooked. Our study identified a bidirectional association between abdominal pain and consultation delay. This pattern may reflect differences in pain characteristics—severe or persistent pain may prompt earlier consultation, whereas mild, intermittent, or temporarily relieved discomfort may delay care. Even early presenters could experience diagnostic delays if they are initially evaluated outside specialized units, as non-specialists may overlook scrotal examination or misattribute symptoms to abdominal disorders (66, 70). Variations in pain characteristics and incomplete medical recording likely contributed to the high heterogeneity in this study. Currently, no clinical studies have yet explored mechanisms underlying different abdominal pain patterns or the role of preoperative ultrasonography in torsion presenting primarily with abdominal pain. In this study, fever was also associated with delayed consultation, possibly reflecting a prolonged disease course or ischemic progression (3). Hydrocele, in contrast, was associated with shorter presentation times, consistent with the results of previous studies (54).

Misdiagnosis and initial presentation to non-tertiary healthcare facilities increased the risk of delayed consultation with lower testicular salvage rates, underscoring the need for improved early recognition in primary care (3, 28, 54, 58). Manual detorsion was associated with a significantly shorter MSD, likely reflecting early clinical recognition of TT. Successful manual detorsion depends on short symptom duration and minimal scrotal swelling and should not delay urgent surgical exploration (73). Interhospital transfer generally facilitates definitive management (74), but its protective effect depends on early recognition and timely specialist involvement. Transfers of children presenting at institutions without surgical capacity or who are uninsured may increase orchiectomy risk (46, 58, 75). Differences in regional referral systems may contribute to the heterogeneity, highlighting the need for further research to understand reasons and timing of transfer. According to the Royal College of Surgeons (UK) guidelines, routine use of color Doppler ultrasonography (CDUS) is not recommended for suspected TT (76). Our pooled analysis confirmed that preoperative ultrasonography did not reduce the risk of delayed consultation, and the pursuit of CDUS requiring interfacility referral may further prolong diagnosis and treatment.

Global disparities in timely consultation reflect socioeconomic and healthcare system differences. High consultation rates in France and Spain likely result from coordinated emergency referral networks, covered health insurance, and public health awareness campaigns (77–79), and physician-led and standardized EMS triage ensures that patients are directly transferred to tertiary centers when needed (80–85). In contrast, prolonged delays in African and Asian countries may stem from limited access to tertiary care and cultural barriers. In India, delayed referral after initial consultation—due to low diagnostic priority for TT, anti-inflammatory treatment, and reliance on Doppler ultrasonography—is the main contributor to a prolonged MSD (28, 29). The differences in delayed treatment in different provinces in China point to the involvement of economic ability and the allocation of medical resources under similar medical systems. In addition, the COVID-19 pandemic contributed to delayed presentation, emphasizing the importance of maintaining emergency pathways during public health crises.

These findings have important implications for clinical practice and public health. Efforts should focus on improving awareness of symptoms among adolescents, parents, and primary healthcare providers to minimize diagnostic delays. Ensuring prompt surgical exploration remains the highest priority. Current UK guidelines recommend that any appropriately trained surgeon should be authorized to perform scrotal exploration to shorten the MSD, particularly in regions with limited urological resources (76, 86).Optimizing referral pathways is also essential, especially by involving clinicians to identify appropriate facilities for patient transfer. Standardized clinical protocols may further enhance early recognition and management. In addition, establishing a national registry could facilitate systematic data collection and benchmarking of treatment timelines and outcomes across healthcare systems.

Several limitations in this study should be acknowledged. First, the number of included studies was limited and unevenly distributed across regions, with a lack of data from low-income countries. Second, high heterogeneity was observed across several pooled analyses, driven by differences in symptom reporting, healthcare pathways, and the small number of studies or cases included per factor. Third, definitions of “delay” and “timely consultation” varied across studies, which may have influenced pooled estimates and comparability. Fourth, the impact of healthcare policies, cultural factors, and patient-level determinants could not be fully assessed because of limited data availability. Fifth, our study relied heavily on published literature rather than national or population-level datasets, which may introduce reporting bias and limit representativeness. Finally, publication bias could not be statistically evaluated because of the small number of studies per factor.

## 5 Conclusion

Despite gradual improvements in early presentation, delayed consultation for testicular torsion remains prevalent worldwide, driven by clinical, healthcare system, and socioeconomic disparities. Enhancing early recognition, optimizing referral pathways, and providing targeted education for patients and primary care providers are critical to reduce delays and maximize testicular salvage. Strengthening emergency infrastructure and ensuring equitable healthcare access are essential for improving outcomes on a global scale.

## Data Availability

The original contributions presented in the study are included in the article/Supplementary Material, further inquiries can be directed to the corresponding author.
