# Supplementary material for: Are we still too late to preserve the testes? A global survey of delayed consultation and risk factors for testicular torsion: a systematic review and meta-analysis
Source: Front Reprod Health. 2026 Feb 24;8:1735652. doi: 10.3389/frph.2026.1735652 (PMC12971663; doi:10.3389/frph.2026.1735652)
Supplement: Supplementary file 1 [file Supplementaryfile1.docx]

**Table S1** Search strategy

| MEDLINE | Embase | Web of Science Core Collection | Cochrane Library |
| --- | --- | --- | --- |
| 1. "Spermatic Cord Torsion"[MeSH] OR "Testicular Torsion"[MeSH]  2. testicular torsion[tiab] OR spermatic cord torsion[tiab] OR torsion of testis[tiab]  3. "Delayed Diagnosis"[MeSH]  4. "Treatment Delay"[MeSH]  5. delayed[tiab] AND diagnosis[tiab]  6. late[tiab] AND diagnosis[tiab]  7. diagnosis delay*[tiab] OR delayed diagnosis[tiab] OR late diagnosis[tiab]  8. treatment delay*[tiab] OR delayed treatment[tiab]  9. 3 OR 4 OR 5 OR 6 OR 7 OR 8  10. 1 OR 2  11. 9 AND 10 | 1. 'testicular torsion'/exp OR 'spermatic cord torsion'/exp  2. testicular torsion:ti,ab OR spermatic cord torsion:ti,ab  3. 'delayed diagnosis'/exp  4. 'treatment delay'/exp  5. delayed:ti,ab AND diagnosis:ti,ab  6. late:ti,ab AND diagnosis:ti,ab  7. diagnosis delay*:ti,ab OR delayed diagnosis:ti,ab OR late diagnosis:ti,ab  8. treatment delay*:ti,ab OR delayed treatment:ti,ab  9. 3 OR 4 OR 5 OR 6 OR 7 OR 8  10. 1 OR 2  11. 9 AND 10 | TS = ("testicular torsion" OR "spermatic cord torsion" OR "torsion of testis")   AND   TS = ("delayed diagnosis" OR "late diagnosis" OR "diagnosis delay" OR "delayed treatment" OR "treatment delay" OR (delayed AND diagnosis) OR (late AND diagnosis)) | #1 MeSH descriptor: [Spermatic Cord Torsion] explode all trees  #2 MeSH descriptor: [Testicular Torsion] explode all trees  #3 "testicular torsion":ti,ab OR "spermatic cord torsion":ti,ab  #4 MeSH descriptor: [Delayed Diagnosis] explode all trees  #5 MeSH descriptor: [Treatment Delay] explode all trees  #6 "delayed diagnosis":ti,ab OR "late diagnosis":ti,ab  #7 diagnosis delay*:ti,ab OR delayed treatment*:ti,ab OR treatment delay*:ti,ab  #8 #4 OR #5 OR #6 OR #7  #9 #1 OR #2 OR #3  #10 #8 AND #9 |
