# Supplementary material for: Are we still too late to preserve the testes? A global survey of delayed consultation and risk factors for testicular torsion: a systematic review and meta-analysis
Source: Front Reprod Health. 2026 Feb 24;8:1735652. doi: 10.3389/frph.2026.1735652 (PMC12971663; doi:10.3389/frph.2026.1735652)
Supplement: Supplementary file 2 [file Supplementaryfile2.docx]

Table S2. Eligibility Criteria and Exposures (P–E–C–O–S Framework)

| Category | Eligibility Criteria |
| --- | --- |
| Population (P) | Patients of any age with clinically diagnosed or surgically confirmed testicular torsion (TT). |
| Exposure (E) | Potential factors influencing delayed presentation or mean symptom duration (MSD), categorized as:  1. Symptoms: nausea or vomiting, fever, abdominal pain, hydrocele  2. First-consultant factors: misdiagnosis, type of first-consultant medical institution, preoperative ultrasound, manual detorsion  3. Transfer: inter-facility transfer  4. Insurance: type and coverage  5. Pandemic: COVID-19 pandemic |
| Comparator (C) | Not applicable |
| Outcomes (O) | Studies reporting at least one of the following:  – Mean symptom duration (MSD)  – Delayed consultation rates  – Orchidectomy rates  – Testicular atrophy  – Misdiagnosis rates  – Inter-facility transfer rates |
| Study design (S) | Observational studies (cohort, case–control, cross-sectional), randomized controlled trials (RCTs), or case series.  Only studies reporting ≥10 patients were included.  Observational studies and RCTs were eligible for quantitative synthesis. |
