# Supplementary material for: Are we still too late to preserve the testes? A global survey of delayed consultation and risk factors for testicular torsion: a systematic review and meta-analysis
Source: Front Reprod Health. 2026 Feb 24;8:1735652. doi: 10.3389/frph.2026.1735652 (PMC12971663; doi:10.3389/frph.2026.1735652)
Supplement: Supplementary file 3 [file Supplementaryfile3.docx]

Table S3 Summary of study characteristics and quality analysis using Newcastle–Ottawa Scale (NOS) or Joanna Briggs Institute Critical Appraisal Tools (JBI)

| Publication | Country (Province) | Study period | Study type | Sample Size | Population | Age (y), mean±sd | <6h n(%) | <12h n(%) | <24h n(%) | Orchiectomy n (%) | Duration of symptoms (h), mean±sd | Quality |
| --- | --- | --- | --- | --- | --- | --- | --- | --- | --- | --- | --- | --- |
| Yilmaz et al (2025)^[1]^ | Turkey | 2019-2023 | RCC | 139 | ≥18y | 36.7±38.5 | - | - | - | 49 (35.3) | 29.9±54.5 | 7 |
| Chen et al (2024)^[2]^ | China, Guangdong | 2005-2022 | RC | 155 | <18y | 8.1±7.2 | - | - | - | 42 (27.1) | 21.0±20.7 | 7 |
| Marcela et al (2025)^[3]^ | Spain | 2020-2022 | PC | 93 | 3m-18y | 13.0±1.5 | - | - | - | - | 6.5±8.5 | 6 |
| Tian et al (2025)^[4]^ | China, Ningxia | 2010-2023 | RC | 176 | 2m-18y | 9.3±3.5 | 6 (3.4) | - | - | 121 (68.8) | 61.7±46.3 | 7 |
| Sasa et al (2025)^[5]^ | Serbia | 2000-2024 | RC | 103 | Children | 12.9±3.0 | 45 (43.7) | 79 (77.7) | - | - | - | 8 |
| Zlatan et al (2025)^[6]^ | Bosnia and Herzegovina | 2009-2023 | RCS | 8 | CTT, Children | 6.1±6.2 | 2 (25.0) | 3 (37.5) | 6 (75.0) | 4 (0.5) | 16.7±11.9 | 9 |
| Zhao et al (2024)^[7]^ | US, New York | 2012-2022 | RCS | 134 | - | 18.7±11.7 | - | - | - | 49 (36.6) | - | 7 |
| Chen et al (2024)^[8]^ | China, Fujian | 2017-2022 | RCC | 120 | <12y | 3.83±1.45 | - | - | - | 26 (21.7) | - | 8 |
| Metin et al (2024)^[9]^ | Turkey | 2015-2024 | RCC | 94 | Manual detorsion | 22.5±8.7 | - | - | - | 74 (78.7) | 7.3±5.9 | 7 |
| Raffee et al (2024)^[10]^ | Jordan | 2009-2010 | RC | 308 |  | 17.2±4.1 | - | - | 110 (35.7) | 194 (63.0) | 92.7±148.9 | 6 |
| Qi et al (2024)^[11]^ | China, Jiangsu | 2017-2022 | RCC | 66 |  | 14.0±3.4 | - | - | - | 27 (40.9) | 23.8±33.1 | 8 |
| Pyrgidis et al (2024)^[12]^ | Germany | 2005-2021 | CSS | 42490 |  | 17.0±6.6 | - | - | - | 11725 (27.6) | - | 8 |
| George et al (2024)^[13]^ | Colombia | 2005-2021 | RCC | 111 | <18y | 13.6±4.7 | - | - | - | 39 (35.1) | 16.0±24.4 | 8 |
| Aya et al (2024)^[14]^ | Japan | 2018-2020 | CSS | 5161 | <21y | - | - | - | - | 7 (7.1) | - | 8 |
| Deng et al (2024)^[15]^ | China, Anhui | 2018-2023 | RCC | 67 | <16y | 8.3±7.6 | 16 (23.9) | 27 (40.3) | 27 (40.3) | 40 (59.7) | 26.3±30.4 | 8 |
| Emily et al (2024)^[16]^ | US, Oregon | 2018-2023 | RC | 133 | 1-18y | - | 68 (51.1) | - | 86 (64.7) | 37 (27.8) | 18.7±34.1 | 9 |
| Buch et al (2024)^[17]^ | Denmark | 2015-2019 | RC | 94 | <16y | 9.0±4.5 | - | - | - | 20 (21.3) | 26.5 | 7 |
| Zhang et al (2023)^[18]^ | China, Anhui | 2004-2021 | RCC | 102 | 1m-16y | 7.5±5.3 | - | 24 (23.5) | 51 (50.0) | 60 (58.8) | - | 8 |
| Yi et al (2023)^[19]^ | China, Chongqing | 2012-2021 | RCC | 1005 | - | - | 239 (23.8) | 407 (40.5) | 473 (47.1) | 532 (52.9) | - | 9 |
| Awad et al (2023)^[20]^ | Bahrain | 2016-2023 | RC | 62 | - | 17.4±1.5 | 45 (72.6) | - | - | - | 5.8±1.2 | 8 |
| Park et al (2023)^[21]^ | South Korea | 2010-2022 | RC | 45 | <18y | 12.5±2.6 | - | - | - | 16 (35.6) | 9.7±17.0 | 8 |
| Emilien et al (2023)^[22]^ | France | 2005-2019 | RC | 1266 | >12y | 21.8±8.0 | 640 (50.6) | 950 (75.0) | 1071 (84.6) | 150 (11.8) | 10.5±9.8 | 8 |
| Feride et al (2023)^[23]^ | Turkey | 2015-2023 | RCS | 11 | 10-17y | 14.3±2.2 | - | - | - | - | 31.0±30.0 | 8 |
| Tiziana et al (2023)^[24]^ | Italy | 2020-2022 | RCS | 72 | 1-14y | 11.9±1.9 | - | - | - | - | 8.2±10.2 | 8 |
| Marcou et al (2023)^[25]^ | Germany | 2001-2021 | RCS | 136 | - | 18.3±5.9 | - | - | - | 48 (35.3) | 19.9 | 8 |
| Mohamed et al (2023)^[26]^ | Uganda | 2022-2023 | PC | 41 | - | - | - | 13 (31.7) | 16 (39.0) | 25 (61.0) | - | 8 |
| Adama et al (2023)^[27]^ | Burkina Faso | 2017-2021 | CSS | 22 | - | 17.8±6.7 | 6 (27.3) | - | - | 11 (50.0) | 78.8±153 | 7 |
| Mao et al (2023)^[28]^ | China, Anhui | 2013-2021 | RC | 73 | 8m-16y | 5.9±3.0 | - | - | - | 37 (50.7) | 47.9±26.8 | 6 |
| Renan et al (2023)^[29]^ | Brazil | 2018-2021 | RC | 87 | - | 23.7±25.9 | 33 (37.9) | - | 53 (60.9) | 49 (56.3) | 24.7 | 7 |
| Rub et al (2023)^[30]^ | Israel | 2009-2019 | RC | 235 | - | 17.9±9.6 | - | - | - | 33 (14.0) | - | 6 |
| Wang et al (2023)^[31]^ | China, Shandong | 2009-2022 | RCC | 292 | - | 16.8 | - | - | - | 140 (60.3) | - | 7 |
| Barkai et al (2023)^[32]^ | Israel | 2015-2020 | RCC | 50 | >18y | 25.0±7.4 | - | - | - | 14 (28.0) | 19.3±26.7 | 7 |
| Peter et al (2022)^[33]^ | Austria | 1999-2020 | RC | 24 | >18y | 15.9±8.0 | - | - | - | 4 (17.4) | 11.2±23.9 | 6 |
| Yu et al (2022)^[34]^ | China, Chongqing | 2004-2020 | RC | 145 | 6m-18y | 10.5±5.4 | - | - | - | 39 (26.9) | - | 9 |
| Miguel et al (2022)^[35]^ | Spain | 2016-2021 | RCC | 117 | <18y | 12.7±4.5 | - | - | - | - | 4.0±3.0 | 7 |
| Elisa et al (2022)^[36]^ | Italy | 2019-2021 | RC | 89 | 1m-18y | - | - | - | - | 19 (21.3) | 11.1±9.6 | 9 |
| Lisa et al (2022)^[37]^ | US, Kentucky | 2015-2019 | RC | 79 | 1m-18y | 13.7±2.2 | - | - | - | 30 (38.0) | 8.0±5.5 | 8 |
| Chun et al (2022)^[38]^ | US, national | 2010-2019 | RC | 890 | 1-18y | 12.0 | - | - | - | 562 (63.2) | - | 8 |
| Chinta et al (2022)^[39]^ | US, Wisconsin | 2017-2020 | RC | 77 | Children | 13.2±3.6 | - | - | - | 19 (24.7) | - | 8 |
| Riyad et al (2022)^[40]^ | UK | 2016-2019 | RC | 187 | <16y | 13.3±2.2 | - | - | - | 61 (32.3) | 13.0±13.9 | 7 |
| Hamarat et al (2022)^[41]^ | Turkey | 2011-2019 | RCS | 43 | - | 13.6±5.6 | - | - | - | - | 11.3±8.8 | 8 |
| Shields et al (2022)^[42]^ | US, Kentucky | 2015-2020 | RC | 140 | 1-18y | 14.0±1.9 | 57 (40.7) | 82 (58.6) | 100 (71.4) | 56 (40.0) | 21.0±31.9 | 9 |
| Lim et al (2022)^[43]^ | South Korea | 2014-2020 | CSS | 21 | 1d-30y | 14.1±5.9 | - | - | 11 (52.4) | 6 (28.6) | - | 8 |
| Lee et al (2022)^[44]^ | US, Washington | 2016-2021 | RC | 206 | ≤18y | 12.6±3.6 | 66 (32.0) | - | 138 (67.0) | 84 (40.8) | 20.0±32.0 | 8 |
| Dan et al (2022)^[45]^ | Israel | 2009-2018 | CSS | 154 | 2d-66y | 16.1±8.4 | - | - | - | 33 (21.4) | 22.3 | 7 |
| Richard et al (2022)^[46]^ | UK | 2007-2019 | CSS | 732 | - | - | - | - | - | 137 (18.7) | - | 7 |
| Benali et al (2022)^[47]^ | France | 2005-2019 | RC | 1802 | >12y | 21.0±8.0 | 892 (67.3) | 1040 (57.7) | 1122 (62.3) | 210 (11.7) | - | 8 |
| Sazgar et al (2022)^[48]^ | Iran | 2015-2020 | CSS | 70 | 3-45y | 20.1±9.6 | - | - | - | - | 4.35±4.72 | 6 |
| Pinar et al (2022)^[49]^ | France | 2015-2020 | RC | 1765 | >12y | - | - | - | - | 219 (12.4) | - | 9 |
| Hasan et al (2022)^[50]^ | Bahrain | 2016-2018 | RCS | 71 | >13y | 20.4±7.0 | - | - | - | 24 (33.8) | 36.0±55.1 | 8 |
| Blasco et al (2022)^[51]^ | Spain, France | 2000-2018 | RCS | 333 | - | - | 210 (63.2) | 260 (78.1) | - | - | - | 7 |
| Choi et al (2022)^[52]^ | South Korea | 2009-2019 | RC | 5694 | - | - | - | - | - | 1411 (24.8) | - | 7 |
| Muraliharan et al (2022)^[53]^ | Australia | 2017-2020 | CSS | 85 | 0-16y | - | - | - | - | - | 20.0 | 7 |
| Leone et al (2021)^[54]^ | Italy | 2010-2019 | RCS | 368 | - | 16.0±6.7 | 150 (46.2) | - | - | 43 (11.7) | - | 9 |
| Zenon et al (2021)^[55]^ | Croatia | 2019-2020 | RC | 119 | 1-17y | 14.7±1.9 | - | 67 (56.3) | 79 (66.4) | 33 (27.7) | 15.2±20.7 | 7 |
| Sarah et al (2021)^[56]^ | US, national | 2019-2020 | RPC | 221 | 2m-18y | 13.3±2.8 | 88 (39.8) | 126 (57.0) | 151 (68.3) | 74 (33.5) | 17.3±24.1 | 7 |
| Athénor et al (2021)^[57]^ | France | 1997-2017 | RC | 60 | CTT | 3.6±5.3 | 4 (23.5) | - | - | 28 (46.7) | - | 8 |
| Zvizdic et al (2021)^[58]^ | Bosnia and Herzegovina | 2011-2016 | RC | 31 | <16y | 11.3±6.3 | - | - | - | 13 (41.9) | 48.0±18.0 | 8 |
| Littman et al (2021)^[59]^ | US, Georgia | 2015-2020 | RC | 57 | 1-18y | 12.9±2.6 | - | - | 41 (71.9) | - | 23.2±35.0 | 7 |
| Komarowska et al (2020)^[60]^ | Poland | 2009-2019 | RCS | 9 | CTT | 8.7±5.0 | - | - | - | 8 (88.9) | 38.8±34.6 | 7 |
| Zheng et al (2021)^[61]^ | China, Shanxi | 2009-2019 | RCC | 204 | ≤30y | 17.1±4.4 | - | - | - | 174 (85.3) | 100.2±99.4 | 7 |
| Shields et al (2021)^[62]^ | US, Kentucky | 2015-2020 | RC | 140 | 1-18y | 13.3±2.2 | 44 (31.4) | 76 (54.3) | 95 (67.9) | 56 (40.0) | 21.6±32.1 | 7 |
| Zhong et al (2021)^[63]^ | China, Shanghai | 2010-2018 | RCS | 15 | Trauma, children | 10.3±3.4 | - | - | - | 10 (66.7) | 138.3±136.4 | 7 |
| Yu et al (2021)^[64]^ | China, Chongqing | 2004-2020 | RCC | 301 | 6m-18y | 10.0±0.3 | - | 81 (26.9) | 114 (37.9) | 110 (36.5) | - | 8 |
| Klinke et al (2020)^[65]^ | Germany | 2013-2019 | RC | 49 | 1-17y | 11.2±6.1 | - | - | 43 (89.6) | 730 (21.6) | - | 8 |
| Sofia et al (2020)^[66]^ | Portugal | 2014-2018 | RC | 122 | <18y | 14.7±2.1 | - | - | - | 18 (14.8) | 11.3±14.8 | 8 |
| Lim et al (2020)^[67]^ | Singapore | 2016-2018 | PC | 43 | <16y | - | 17 (39.5) | 21 (48.8) | 27 (62.8) | - | - | 8 |
| Göger et al (2020)^[68]^ | Turkey | 2012-2020 | RCC | 140 | - | 12.7±2.7 | - | - | - | 62 (44.3) | 33.8±29.8 | 7 |
| Dias et al (2020)^[69]^ | Brazil | 2012-2018 | RCS | 505 | - | 16.4±3.5 | - | - | - | - | 22.0±36.7 | 8 |
| Tian et al (2020)^[70]^ | China, Chongqing | 2009-2019 | RCC | 113 | 2-17y | 10.0±3.5 | - | 41 (36.3) | 71 (62.8) | - | - | 9 |
| Merder et al (2020)^[71]^ | Turkey | 2016-2018 | PC | 88 | 0-18y | 17.7±4.4 | 59 (67.0) | - | - | 27 (30.7) | 9.2±13.0 | 9 |
| Taskinen et al (2020)^[72]^ | Finland | 2000-2018 | RC | 54 | Children | 13.8±1.3 | - | - | - | 7 (13.0) | 13.3±9.9 | 9 |
| Kumar et al (2020)^[73]^ | India | 2007-2017 | RCS | 50 | <18y | 3.2±4.5 | - | - | - | 22 (44.0) | 79.2 | 6 |
| Zhang et al (2020)^[74]^ | China, Beijing | 1996-2014 | RC | 122 | - | 5.0±15.8 | 52 (42.6) | - | 93 (76.2) | 72 (59.0) | - | 6 |
| Guo et al (2020)^[75]^ | China, Hubei | 2007-2017 | RCS | 57 | <14y | 6.6±3.3 | 0 (0) | 0 (0) | 2 (3.5) | 16 (28.1) | 83.0±19.4 | 9 |
| Nandwani et al (2020)^[76]^ | UK | 2006-2017 | RCS | 231 | - | 32.0±54.8 | 180 (77.9) | 209 (90.4) | - | 34 (14.7) | 113.8±247.8 | 9 |
| Tanaka et al (2020)^[77]^ | Japan | 2012-2019 | RCS | 38 | ≤15y | 10.6±1.5 | - | - | - | 15 (39.5) | 41.4±29.4 | 8 |
| Hosokawa et al (2020)^[78]^ | Japan | 2014-2019 | RCS | 14 | Children | 11.4±3.8 | 5 (35.7) | 6 (42.9) | 7 (50.0) | - | 27.9±31.0 | 7 |
| Obi et al (2020)^[79]^ | Nigeria | 2011-2019 | PCS | 31 | - | 22.6±7.3 | 10 (37.0) | 19 (70.4) | 22 (81.5) | 4 (12.9) | - | 8 |
| Feng et al (2020)^[80]^ | China, Zhejiang | 2009-2018 | RC | 136 | 1-16y | 10.3±3.8 | - | - | - | 87 (64.0) | 14.8±1.8 | 7 |
| Overholt et al (2019)^[81]^ | US, West Virginia | 2008-2016 | RC | 23 | - | 17.1±5.9 | - | - | - | 6 (26.1) | 10 | 7 |
| Olivia et al (2019)^[82]^ | US, Tennessee | 2016-2018 | RC | 41 | 6m-18y | - | - | - | 25 (61.0) | 22 (54.5) | 25.3±28.0 | 7 |
| Goetz et al (2019)^[83]^ | US, Wisconsin | 2010-2017 | RC | 91 | <18y | 12.7±14.8 | - | - | - | 28 (30.8) | - | 8 |
| Gold et al (2019)^[84]^ | Israel | 1994-2014 | RCC | 219 | - | 15.0±7.7 | 111 (50.7) | 136 (62.1) | 159 (72.6) | 112 (51.1) | 12.7±14.8 | 7 |
| Zee et al (2019)^[85]^ | US, Wisconsin | 2004-2018 | NI | 135 | <18y | 13.4±2.2 | - | - | - | 37 (27.4) | 8.4±4.0 | 7 |
| Wang et al (2019)^[86]^ | China, Shandong | 2015-2016 | RCS | 9 | 10-17y | 13.9±2.1 | - | - | - | 7 (77.8) | 29.7±21.8 | 7 |
| He et al (2019)^[87]^ | China, Beijing | 2006-2018 | RCC | 112 | Children | 10.6±4.5 | - | - | - | - | 54.1±76.5 | 7 |
| Afsarlar et al (2019)^[88]^ | US, Texas | 2010-2017 | RCC | 223 | <18y | 12.2±4.4 | 63 (28.3) | 88 (39.5) | 122 (54.7) | 108 (48.4) | 39.7±47.8 | 7 |
| Romao et al (2019)^[89]^ | Canada | 2010-2014 | RC | 1713 | <18y | 14.3±2.2 | - | - | - | 473 (27.6) | - | 8 |
| Peeraully et al (2019)^[90]^ | UK | 2008-2016 | RC | 100 | Children | - | - | - | - | 34 (34.0) | 8.8±9.7 | 7 |
| Chan et al (2019)^[91]^ | Canada | 2012-2017 | RC | 46 | - | - | - | - | - | 8 (17.4) | 7.9 | 7 |
| Huang et al (2019)^[92]^ | US, Tennessee | 2008-2017 | RCC | 133 | <20y | 12.1±5.0 | 52 (39.1) | 81 (60.9) | 85 (63.9) | 60 (45.1) | 3.3±2.5 | 7 |
| Jang et al (2019)^[93]^ | South Korea | 2005-2015 | RCC | 60 | - | 14.7±2.5 | 26 (43.3) | 45 (75.0) | 60 (100) | 38 (63.3) | 8.3±5.0 | 8 |
| Buicko et al (2018)^[94]^ | US, national | 2005–2009 | RC | 1051 | <18y | 11.9±5.2 | - | - | - | 416 (39.6) | - | 9 |
| Yan et al (2018)^[95]^ | China, Hubei | 2007-2016 | RC | 107 | 1-23y | 12.5±5.5 | 13 (12.1) | 21 (19.6) | 51 (47.7) | 64 (59.8) | 48.0±43.2 | 8 |
| Yecies et al (2018)^[96]^ | US, Pennsylvania | 2003-2017 | RCC | 104 | Children | 12.6±3.9 | - | - | - | 50 (48.1) | 23.2±32.1 | 8 |
| Arevalo et al (2018)^[97]^ | US, Texas | 2012-2016 | RC | 68 | Children | 11.0±4.1 | 31 (47.7) | - | - | 38 (55.9) | - | 6 |
| Manohar et al (2018)^[98]^ | India | 2007-2016 | RC | 45 | <28y | 17.3±5.0 | - | - | - | - | 116.8±88.5 | 7 |
| Frohlich et al (2017)^[99]^ | US, Massachusetts | 2013-2015 | PC | 19 | 3m-18y | 12.5±4.3 | 7 (46.7) | 10 (66.7) | 13 (86.7) | 4 (21.1) | - | 8 |
| Demirbas et al (2017)^[100]^ | Turkey | 2011-2015 | RC | 57 | - | - | - | - | - | 9 (15.8) | - | 7 |
| Janae et al (2017)^[101]^ | US, Ohio | 2011-2016 | RC | 125 | Children | 13.4±4.3 | - | - | 89 (71.2) | 45 (36.0) | 21.4±32.1 | 8 |
| Bayne et al (2017)^[102]^ | US, Washington | 2005-2015 | RCC | 208 | Children, ≥2y | 12.8±3.4 | - | - | 114 (54.8) | 122 (58.7) | - | 7 |
| Nevo et al (2017)^[103]^ | Israel | 2008-2014 | RCC | 100 | Children | 12.3±2.9 | - | - | - | 40 (40.0) | 17.6±22.6 | 8 |
| Naouar et al (2017)^[104]^ | Tunisia | 1999-2015 | RCS | 13 | - | 13.0±13.2 | 4 (30.8) | 7 (53.8) | 12 (92.3) | 34 (23.1) | 14.5±12.1 | 8 |
| Samson et al (2017)^[105]^ | US, New York | 2009-2014 | DA | 147 | <18y | - | 35 (31.3) | 54 (36.7) | 66 (44.9) | 34 (23.1) | - | 7 |
| Bayne et al (2017)^[106]^ | US, Washington | 2005-2015 | RC | 218 | Children | 12.5±4.2 | - | - | - | 37 (17.0) | - | 7 |
| Sood et al (2016)^[107]^ | US, national | 1998-2010 | RC | 17478 | <18y | - | - | - | - | 6711 (38.4) | - | 8 |
| Afsarlar et al (2016)^[108]^ | US, Texas | 2013-2014 | RC | 57 | Children | 11.9±4.9 | - | - | - | 29 (50.9) | - | 8 |
| Ugwumba et al (2016)^[109]^ | Nigeria | 1999-2019 | CSS | 34 | 16-50y | - | 1 (2.9) | 6 (17.6) | 8 (23.5) | - | - | 6 |
| Ayvaz et al (2015)^[110]^ | Turkey | 2010-2014 | RC | 24 | Children | - | 8 (33.3) | 16 (66.7) | 24 (100) | 6 (25.0) | - | 7 |
| Johnston et al (2015)^[111]^ | New Zealand | 2001-2010 | RC | 35 | - | - | - | - | - | 7 (20.0) | 5.3±6.3 | 7 |
| Ford et al (2015)^[112]^ | UK | 2011-2013 | RC | 48 | Children | - | - | - | - | - | 34.0±29.7 | 6 |
| Puneeta et al (2015)^[113]^ | US, California | 2005-2011 | RC | 114 | Children | 11.3±4.7 | 50 (44.2) | - | - | 51 (44.7) | 15.4±23.6 | 8 |
| Güneş et al (2015)^[114]^ | Turkey | 2007-2013 | RCC | 72 | - | 17.9±4.5 | 32 (44.4) | 40 (55.6) | 52 (72.2) | 29 (40.3) | - | 8 |
| Benedetto et al (2014)^[115]^ | Italy | 2012-2013 | RCC | 14 | Children | - | - | - | - | 6 (42.9) | 12.7±3.8 | 6 |
| Moslemi et al (2014)^[116]^ | Iran | 2008-2012 | RCS | 68 | Children | 16.7±6.1 | 19 (27.9) | 22 (32.4) | 35 (51.5) | 29 (42.6) | - | 7 |
| Lee et al (2014)^[117]^ | South Korea | 2006-2011 | RC | 1591 | - | - | - | - | - | 386 (24.3) | - | 8 |
| Yiee et al (2013)^[118]^ | US, California | 2008-2010 | RC | 2794 | - | - | - | - | - | 866 (31.0) | - | 9 |
| Pogorelić et al (2013)^[119]^ | Croatia | 1999-2012 | RC | 8 | CTT, Children | 10.4±6.5 | 3 (37.5) | 4 (50.0) | - | 4 (50.0) | 28.1±24.8 | 6 |
| Huang et al (2013)^[120]^ | China, Taiwan | 1997-2010 | RCC | 86 | <25y | 12.0±6.2 | - | - | - | 22 (25.6) | - | 9 |
| Chen et al (2013)^[121]^ | China, Taiwan | 1996-2008 | RCC | 61 | - | 16.2±8.3 | - | - | - | 18 (29.5) | - | 9 |
| Liang et al (2013)^[122]^ | Canada | 2008-2011 | RCC | 35 | 1m-17y | 12.2 | - | - | - | 1 (2.9) | 20.3 | 7 |
| Barbosa et al (2013)^[123]^ | US, Massachusetts | 1965-2011 | RC | 51 | 3m-18y | 12.0 | - | - | - | 15 (29.4) | 25.0±35.8 | 9 |
| Boettcher et al (2012)^[124]^ | Germany | 2008-2009 | RCC | 19 | Children | 11.1 | - | 10 (52.6) | - | - | 17.2±19.1 | 8 |
| Yang et al (2011)^[125]^ | China, Chongqing | 1990-2008 | RCS | 103 | Children | 8.6±3.7 | 22 (21.4) | 29 (28.2) | 36 (35.0) | 72 (69.9) | 95.5±59.5 | 7 |
| Molokwu et al (2011)^[126]^ | UK | 1998-2008 | PCS | 173 | - | 25.8±18.3 | - | - | - | 16 (9.2) | - | 8 |
| Bayne et al (2010)^[127]^ | US, California | 2003-2008 | RC | 97 | Children | 11.6 | - | - | - | 62 (63.9) | 39.5 | 9 |
| Tali et al (2010)^[128]^ | Israel | 2005-2007 | RCS | 17 | 1w-18y | - | - | - | - | 3 (17.6) | - | 9 |
| Chmelnik et al (2010)^[129]^ | Germany | 2001-2008 | RCS | 25 | Children | - | - | - | - | 10 (40.0) | 21.4 | 9 |
| Kaye et al (2008)^[130]^ | US, New York | 2003-2007 | RCS | 55 | 1-17y | - | - | - | - | 39 (70.9) | 25.1±23.5 | 8 |
| Hayn et al (2008)^[131]^ | US, Pennsylvania | 1988-2006 | RC | 17 | 7-18y | 14.0±2.0 | - | - | - | 6 (35.3) | - | 7 |
| Liu et al (2007)^[132]^ | China, Taiwan | 1993-2004 | RCS | 41 | ≤25y | 14.0±5.7 | 15 (36.6) | 19 (46.3) | 23 (56.1) | 24 (58.5) | - | 8 |
| Mäkelä et al (2007)^[133]^ | Finland | 1977-1995 | RCS | 100 | <17y | - | 36 (36.0) | 52 (52.0) | 75 (75.0) | 49 (49.0) | - | 8 |
| Karmazyn et al (2005) | US, Indiana | 1997-2002 | RCC | 41 | Children | 10.4±5.5 | 20 (48.8) | - | - | 4 (9.8) | - | 8 |
| Hunayan et al (2004)^[134]^ | Kuwait | 1999-2002 | PC | 63 | - | 18.3±8.5 | - | - | 51 (81.0) | 11 (17.5) | 18.8±41.5 | 7 |
| Hegarty et al (2001)^[135]^ | Ireland | 1993-1998 | RC | 33 | 3m-37y | 15.7 | - | - | - | 4 (12.1) | 20.0 | 6 |
| Rivers et al (2000)^[136]^ | US, Michigan | 1996-1998 | RC | 11 | - | - | - | - | - | - | 8.5±13.5 | 6 |
| Barada et al (1998)^[137]^ | US, New York | 1977-1988 | RC | 30 | - | - | 11 (36.7) | 22 (73.3) | - | 9 (30.0) | - | 8 |
| Anderson et al (1988)^[138]^ | UK | 1960-1984 | RC | 624 | - | - | 226 (36.2) | 325 (52.1) | 410 (65.7) | 238 (38.1) | - | 8 |
| Jones et al (1986)^[139]^ | UK | 1972-1983 | RC | 179 | >15y | - | - | 83 (46.4) | 114 (63.7) | 45 (25.1) | - | 8 |
| Udeh et al (1985)^[140]^ | Nigeria | 1979-1983 | RCS | 74 | - | - | - | - | 27 (47.4) | - | - | 6 |
| Bartsch et al (1980)^[141]^ | Austria | 1966-1977 | RCS | 30 | - | - | - | 20 (66.7) | 22 (73.3) | 7 (8.6) | - | 7 |
| Dimopoulos et al (1976)^[142]^ | Greece | - | RCS | 40 | >14y | - | 12 (30.0) | - | - | 9 (22.5) | - | 6 |
| Mehmetoğlu et al (2023)^[23]^ | Turkey | 2015-2023 | RCS | 11 | 10-17y | 14.0±2.0 | 2 (18.2) | 4 (36.4) | 7 (63.6) | 4 (36.4) | 31.0±30.0 | 7 |
| Peretti et al (2019)^[143]^ | Italy | 2014-2016 | RCC | 8 | - | 13.3±2.2 | 4 (50.0) | - | - | 4 (50.0) | - | 6 |
| Tryfonas et al (1994)^[144]^ | Greece | 1979-1991 | RCS | 25 | <14y | - | - | 8 (32.0) | 11 (44.0) | 11 (14.7) | - | 7 |
| Zhong et al (2021)^[145]^ | China, Shanghai | 2010-2019 | RCC | 22 | CTT,  1m-16y | 6.6±7.8 | 17 (77.3) | - | - | 8 (36.4) | 27.1±33.0 | 7 |
| Rampaul et al (1998)^[146]^ | Trinidad and Tobago | - | RCS | 28 | 3m-37y | - | 11 (39.3) | - | 20 (71.4) | 6 (27.3) | 7.5 | 8 |
| Li et al (2013)^[147]^ | China, Henan | 2008-2011 | RCC | 54 | Children | 9.4 | - | - | - | - | 14.0±8.8 | 8 |
| Liu et al (2003)^[148]^ | China, Henan | 1991-2002 | RCS | 34 | 2d-18y | 9.0±4.5 | - | - | 6 (17.6) | - | - | 9 |
| Xiao et al (2025)^[149]^ | China, Jiangsu | 2014-2022 | RCS | 82 | 7-30y | 16.6±5.3 | 26 (31.7) | 46 (56.1) | 52 (63.4) | 33 (40.2) | 3.7±1.7 | 8 |
| Lihan et al (2010)^[150]^ | China, Zhejiang | 2005-2009 | RCS | 37 | 4-29y | - | - | 26 (70.2) | 31 (83.8) | 13 (35.1) | - | 10 |
| Liming et al (2018)^[151]^ | China, Tianjin | 2001-2017 | RCS | 95 | - | 19.6±7.3 | - | - | - | 41 (43.2) | - | 9 |
| Xianjin et al (2011)^[151]^ | China, Shanghai | 2004-2010 | RCS | 20 | 15-30y | 22.1±3.8 | 4 (20.0) | - | 6 (30.0) | 12 (60.0) | 177.6±539.0 | 8 |
| Wenxuan et al (2010)^[152]^ | China, Tianjin | 2000-2009 | RCS | 60 | 13-42y | 18.1±6.6 | 15 (25.0) | 25 (41.7) | 28 (46.7) | 27 (45.0) | - | 9 |
| Qun et al (2015)^[153]^ | China, Jiangsu | 2008-2014 | RCS | 49 | 13-52y | 21.0±6.0 | 2 (4.1) | 17 (34.7) | 26 (53.1) | - | - | 9 |
| Guofang et al (2004)^[154]^ | China, Henan | 2001-2004 | RCS | 45 | 8-37y | 14.6±7.3 | 2 (4.4) | - | 19 (42.2) | - | - | 6 |
| Rujian et al (2008)^[155]^ | China, Zhejiang | 1999-2006 | RCS | 42 | 6-51y | - | 4 (9.5) | - | 29 (69.0) | 21 (50.0) | - | 7 |
| Shicheng et al (2013)^[156]^ | China, Zhejiang | 2006-2012 | RC | 36 | 10-28y | 18.0±4.0 | 12 (33.3) | - | 33 (91.7) | 22 (61.1) | 9.3±8.4 | 7 |
| Jianyong et al (2008)^[157]^ | China, Hebei | 1996-2006 | RCS | 30 | 5m-28y | 16.2 | 7 (23.3) | - | 16 (53.3) | 19 (63.3) | - | 7 |
| Yangguo et al (2011)^[158]^ | China, Henan | 1987-2009 | RC | 25 | 2-44y | - | 12 (48.0) | - | 16 (64.0) | 15 (60.0) | - | 7 |
| Li et al (2020) | China, Liaoning | 2015-2017 | RCS | 20 | 5-37y | - | - | - | 9 (60.0) | 5 (25.0) |  | 9 |
| Wenlong et al (2005)^[159]^ | China, Hebei | 1993-2003 | RCS | 19 | 6-39y | - | 5 (26.3) | - | - | 14 (73.7) | - | 7 |
| Yongbiao et al (2007)^[160]^ | China, Henan | 1998-1005 | RCS | 16 | 9-50y | - | 1 (6.3) | 3 (18.8) | 5 (31.3) | 11 (78.6) | - | 7 |
| chengshang et al (2012)^[161]^ | China, Henan | 2004-2010 | RCS | 27 | 4-14y | - | 7 (25.9) | - | 16 (59.3) | 15 (62.5) | - | 7 |
| Jianjun et al (2024)^[162]^ | China, Hunan | 2017-2021 | RCC | 108 | Children | 8.9±5.1 | - | - | - | 82 (75.2) | 69.8±156.0 | 8 |
| Xiaofeng et al (2021)^[163]^ | China, Hunan | 2013-2019 | RC | 85 | 10-58y | - | - | - | - | 50 (58.8) | - | 7 |
| Dezhi et al (2007)^[164]^ | China, Liaoning | 1991-2006 | RCS | 56 | 4-15y | 8.5 | 20 (35.7) | - | 48 (85.7) | 23 (41.1) | - | 8 |
| Tianqi et al (2001)^[165]^ | China, Fujian | 1996-1999 | RCS | 18 | 1d-34y | 19 | 5 (27.8) | - | - | 13 (72.2) | - | 6 |
| Guliev et al (2023)^[166]^ | Russia | 2020-2023 | RCS | 33 | 18-35y | 24.2±5.1 | 18 (54.5) | 24 (72.7) | 31 (93.9) | 4 (12.1) | - | 7 |
| Murithi et al (2017)^[167]^ | Kenya | 2011-2015 | RCS | 90 | - | 19.0±6.0 | - | - | - | 65 (72.2) | 19.0±6.0 | 7 |
| Ibingira et al (2001)^[168]^ | Uganda | 1993-1997 | RCS | 101 | 5-40y | - | - | 24 (23.5) | 47 (46.5) | 65 (64.4) | - | 6 |
| Derbew et al (2015)^[169]^ | Ethiopia | 2012-2014 | RCS | 7 | 5d-18y | - | 1 (14.3) | 3 (28.6) | 4 (14.3) | 2 (28.6) | - | 7 |
| Gnassingbe et al (2009)^[170]^ | Mali | 2003-2007 | RCS | 17 | 7d-15y | 9 | - | - | - | 6 (35.3) | - | 8 |
| Muguti et al (2009)^[171]^ | Zimbabwe | 1987-1991 | RCS | 90 | 2m-32y | 17 | - | - | - | 32 (35.6) | - | 7 |
| Okorie et al (2011)^[172]^ | Cameroon | 2003-2011 | PCS | 16 | 13-32y | 19.8±4.8 | - | - | - | 16 (100.0) | - | 9 |
| Fayzuloev et al (2009)^[173]^ | Russia | 2000-2007 | PCS | 24 | Children | - | - | - | - | 16 (66.7) | - | 7 |
| Kartashev et al (2019)^[174]^ | Russia | - | RCS | 127 | 1d-17y |  | 46 (36.2) | - | - | 40 (31.5) | - | 8 |
| Komarova et al (2016)^[175]^ | Russia | 2005-2014 | RCS | 90 | Children | - | 31 (34.4) | 49 (54.4) | 59 (65.6) | - | - | 7 |
| Doronin et al (2016)^[176]^ | Belarus | 2003-2024 | RCS | 17 | 17-39y | 22.8±5.4 | - | 9 (52.9) | 11 (64.7) | - | - |  |

CTT, cryptorchid testicular torsion; RC, retrospective cohort study; RCC, retrospective case-control study; PC, Prospective cohort study; RCS, retrospective case series; PCS, prospective case series; CSS, Cross-sectional study; RPC, retrospective-prospective cohort study; NI, Non-Randomized Intervention Studies; DA, diagnostic accuracy study

**Reference**

1. Yilmaz AB, Ozercan AY, Erkan A, Elmaagac B, Senel C, Keten T, et al. Predictive value of hematologic parameters and HALP score for testicular viability in adults with testicular torsion: A multicentric study. *Int J Urol* 2025; 32(3):300-306.doi:10.1111/iju.15650.

2. Chen P, Huang W, He Y, Sun M, Sun X, Huang Y, et al. A nomogram for predicting risk factors of testicular salvage after testicular torsion in children. *Int J Urol* 2024; 31(5):568-574.doi:10.1111/iju.15420.

3. Valdivieso-Castro MP, Vázquez-Gómez L, Olabarri M, Presno-López I, Espinosa-Góngora R, Orejuela-Ribera A, et al. Clinical Prediction Rules for Identifying Children With Testicular Torsion: A Multicenter Prospective Study. *Pediatr Emerg Care* 2025; 41(8):620-627.doi:10.1097/pec.0000000000003394.

4. Tian Y, Xing X, Cai H, ShengZhang, Ma X, Liu Q. Predictors of testicular salvage failure in pediatric testicular torsion: a retrospective analysis of clinical and imaging risk factors. *Pediatr Surg Int* 2025; 41(1):210.doi:10.1007/s00383-025-06124-5.

5. Milivojevic S, Topalovic D, Dasic I, Cvejic S, Filipovic I, Mihajlovic S, et al. Testicular Atrophy Following Torsion in Pediatric Patients: Results of a Long-Term Follow-Up. *Urology* 2025; 198:118-124.doi:10.1016/j.urology.2025.01.017.

6. Zvizdic Z, Jonuzi A, Glamoclija U, Zvizdic D, Vranic S. Clinical characteristics and outcome of children with acute cryptorchid testicular torsion: A single-center, retrospective case series study. *Am J Emerg Med* 2024; 82:4-7.doi:10.1016/j.ajem.2024.05.010.

7. Zhao K, Lu JY, Shkolnik B, Davis RB. Practice Patterns Affecting Delays in Care of Testicular Torsion. *Urology* 2024; 184:83-86.doi:10.1016/j.urology.2023.11.007.

8. Zeng C, Fang L, Li W, Chen H. Diagnostic efficacy and value of ultrasound in children's scrotal testicular torsion: A retrospective analysis. *Medicine (Baltimore)* 2024; 103(41):e39884.doi:10.1097/md.0000000000039884.

9. Yığman M, Ekenci BY, Durak HM, Karakoyunlu AN. Predictive factors for manual detorsion success in testicular torsion. *Int Urol Nephrol* 2024; 56(12):3797-3804.doi:10.1007/s11255-024-04151-0.

10. Raffee L, Bani Ali M, Alawneh K, Negresh N, Alawneh H, Al-Shatnawi A, et al. Seasonal Variations in Testicular Torsion: A Retrospective Study. *Cureus* 2024; 16(12):e76508.doi:10.7759/cureus.76508.

11. Qi X, Yu J, Ding X, Wang Y, Zhu H. Manual reduction in testicular torsion and subsequent treatment after successful reduction: a series of reports in a single institution. *Front Pediatr* 2024; 12:1362104.doi:10.3389/fped.2024.1362104.

12. Pyrgidis N, Apfelbeck M, Stredele R, Rodler S, Kidess M, Volz Y, et al. The impact of health care on outcomes of suspected testicular torsion: results from the GRAND study. *World J Urol* 2024; 42(1):309.doi:10.1007/s00345-024-05015-z.

13. Moran GW, Wang CN, Chung R, Movassaghi M, Carpenter CP, Finkelstein JB. Atypical Presentation Delays Treatment of Pediatric Testicular Torsion. *Pediatr Emerg Care* 2024; 40(4):255-260.doi:10.1097/pec.0000000000002969.

14. Hiramatsu A, Den H, Morita M, Ogawa Y, Fukagai T, Kokaze A. A nationwide epidemiological study of testicular torsion: Analysis of the Japanese National Database. *PLoS One* 2024; 19(3):e0297888.doi:10.1371/journal.pone.0297888.

15. Deng QF, Yang C, Mao C, Chu H. Clinical and hematological analysis of testicular torsion in children. *Front Pediatr* 2024; 12:1399349.doi:10.3389/fped.2024.1399349.

16. Clennon EK, Stefanko A, Guerre M, Hecht SL, Austin JC, Seideman CA. The ball's in your court: Trends, causes, outcomes, and costs of patient transfer for pediatric testicular torsion. *J Pediatr Urol* 2024; 20(5):929-936.doi:10.1016/j.jpurol.2024.05.010.

17. Buch Kjeldgaard A, Kinder-Klausen MS, Nerstrøm M, Cohen J, Henriksen BM, Thorup JM. The impact of ultrasound on testicular loss in cases of testicular torsion in children. *Pediatr Surg Int* 2024; 40(1):83.doi:10.1007/s00383-024-05663-7.

18. Zhang K, Zhang Y, Chao M. Clinical characteristics and identification of risk factors of testicular torsion in children: A retrospective study in a single institution. *Front Surg* 2022; 9:1040487.doi:10.3389/fsurg.2022.1040487.

19. Yi H, Wang D, Wu X, Gan X, Wang D, Zhao X, et al. Analysis of factors associated with delayed diagnosis and treatment of testicular torsion in 1005 cases from Chongqing city, China: a cross-sectional study. *Sci Rep* 2023; 13(1):22765.doi:10.1038/s41598-023-49820-9.

20. Awad N, Abdulaziz K, Malalla B, Al Aradi AH, Al Rashed AA. Degrees of Testicular Atrophy Following Orchidopexy for Testicular Torsion. *Cureus* 2023; 15(12):e50543.doi:10.7759/cureus.50543.

21. Park JS, Kim D, Chun MK, Choi SJ, Lee JS, Ryu JM, et al. Implementing Point-of-Care Ultrasound for Acute Scrotal Pain in the Pediatric Emergency Department: Screening Testicular Torsion and Patient Flow Analysis. *J Ultrasound Med* 2023; 42(12):2757-2764.doi:10.1002/jum.16312.

22. Seizilles de Mazancourt E, Khene Z, Sbizerra M, Kaulanjan K, Plassais C, Bardet F, et al. Cut-off time for surgery and prediction of orchiectomy in spermatic cord torsion: a retrospective multicentric study over 15 years. *World J Urol* 2023; 41(12):3789-3794.doi:10.1007/s00345-023-04671-x.

23. Mehmetoğlu F. Patient Query in Adolescent Testicular Torsion Cases: "Was it Necessary to Mention My Scrotal Pain?". *Cureus* 2023; 15(10):e47386.doi:10.7759/cureus.47386.

24. Russo T, Cozzi DA, Gaglione G, Ceccanti S. The Role of Manual Detorsion in Pediatric Testicular Torsion During the Global COVID-19 Pandemic: Experience From 2 Centres. *Urology* 2023; 180:227-234.doi:10.1016/j.urology.2023.04.025.

25. Marcou M, Hartmann A, Wullich B, Apel H, Hirsch-Koch K. Retrospective histological evaluation of orchiectomy specimens following testicular torsion reveals a 10% incidence of reversible injury. Is it time for a change of strategy? *Andrology* 2023; 11(6):1044-1049.doi:10.1111/andr.13368.

26. Mohamed MA, Abraha D, Olasinde AA, Kiswezi A, Molen SF, Muhumuza J, et al. Testicular salvageability and its predictors among patients with testicular torsion in a resource limited setting: a multicentre longitudinal study. *BMC Surg* 2023; 23(1):248.doi:10.1186/s12893-023-02118-z.

27. Ouattara A, Paré AK, Yé D, Traoré MZ, Simporé M, Rouamba M, et al. Prevalence and Management of Spermatic Cord Torsion (SCT): A Five-Year Review in Souro Sanou University Hospital of Bobo-Dioulasso (Burkina Faso). *Res Rep Urol* 2023; 15:381-385.doi:10.2147/rru.S419786.

28. Mao CK, Cao YS, Liu X, Peng B, Chu H, Deng QF, et al. The diagnosis and treatment of testicular torsion in children with non-scrotal initial symptoms. *Front Pediatr* 2023; 11:1176345.doi:10.3389/fped.2023.1176345.

29. Hayashi RM, Hidaka AK, Glina FPA, Smaidi K, Pazeto CL, Nascimento FJ, et al. Spermatic cord torsion: a retrospective analysis. *Einstein (Sao Paulo)* 2023; 21:eAO0238.doi:10.31744/einstein_journal/2023AO0238.

30. Rub R, Lidawi G, Laukhtina E, Asali M, Majdoub M. Impact of seasonal variations on incidence and laterality of testicular torsion. *Int J Biometeorol* 2023; 67(5):857-863.doi:10.1007/s00484-023-02460-0.

31. Wang X, Wang T, Wu G, Xu Z, Sun F, Liu C, et al. Testicular Torsion in Jiaodong Peninsula: A 14-Year Multicenter Retrospective Study. *Arch Esp Urol* 2023; 76(9):690-695.doi:10.56434/j.arch.esp.urol.20237609.84.

32. Barkai E, Dekalo S, Yossepowitch O, Ben-Chaim J, Bar-Yosef Y, Beri A, et al. Complete Blood Count Markers and C-Reactive Protein as Predictors of Testicular Viability in the Event of Testicular Torsion in Adults. *Urol Int* 2023; 107(8):801-806.doi:10.1159/000531145.

33. Törzsök P, Steiner C, Pallauf M, Abenhardt M, Milinovic L, Plank B, et al. Long-Term Follow-Up after Testicular Torsion: Prospective Evaluation of Endocrine and Exocrine Testicular Function, Fertility, Oxidative Stress and Erectile Function. *J Clin Med* 2022; 11(21).doi:10.3390/jcm11216507.

34. Yu CJ, Zhao J, Luo J, Hong YF, Zhao TX, Wen S, et al. Long-term follow-up results of testicular torsion in children. *Asian J Androl* 2022; 24(6):653-659.doi:10.4103/aja2021127.

35. Delgado-Miguel C, García A, Muñoz-Serrano AJ, López-Pereira P, Martínez-Urrutia MJ, Martínez L. The role of neutrophil-to-lymphocyte ratio as a predictor of testicular torsion in children. *J Pediatr Urol* 2022; 18(5):697.e691-697.e696.doi:10.1016/j.jpurol.2022.09.010.

36. Zambaiti E, Cerchia E, Guanà R, Scottoni F, Giannotti G, Dalla Rosa D, et al. Testicular torsion during the COVID-19 pandemic: Results of a multicenter study in northern Italy. *J Pediatr Urol* 2022; 18(4):530.e531-530.e536.doi:10.1016/j.jpurol.2022.06.010.

37. Shields LBE, Daniels MW, Peppas DS, White JT, Mohamed AZ, Canalichio K, et al. Surge in testicular torsion in pediatric patients during the COVID-19 pandemic. *J Pediatr Surg* 2022; 57(8):1660-1663.doi:10.1016/j.jpedsurg.2021.07.008.

38. Chun B, Colaco M, Fox JA, Cannon GM, Schneck FX, Chaudhry R, et al. Salvage Surgery Rates for Pediatric Testicular Torsion: Comparison of the Pre- and Post-Quality Metric Eras. *J Urol* 2022; 208(2):441-447.doi:10.1097/ju.0000000000002677.

39. Chinta SS, Gray MP, Kopetsky M, Baumer-Mouradian SH, Drendel AL, Roth E, et al. Quality Improvement Project to Improve the Timeliness of Care for Children With Testicular Torsion in the Emergency Department. *Pediatr Qual Saf* 2022; 7(4):e576.doi:10.1097/pq9.0000000000000576.

40. Peeraully R, John M, Ellis R, Green S, Jancauskaite M, Smart T, et al. Does decentralisation of surgical management improve outcomes for paediatric testicular torsion? *J Pediatr Urol* 2022; 18(3):302.e301-302.e308.doi:10.1016/j.jpurol.2022.03.020.

41. Hamarat MB, Dönmez M, Sezgin T, Ünlü MZ, Kocaoğlu C, Özkent MS, et al. Testicular volume loss in the long-term follow-up after surgical detorsion of the testis. *Pediatr Surg Int* 2022; 38(6):907-911.doi:10.1007/s00383-022-05118-x.

42. Shields LB, Daniels MW, Peppas DS, Rosenberg E. Impact of Distance From the Hospital and Patient Transfer on Pediatric Testicular Torsion Outcomes. *Cureus* 2022; 14(5):e25284.doi:10.7759/cureus.25284.

43. Lim Y, Hwang J, Park J, Kim YC, Min SJ. A simplified ultrasound approach to diagnose testicular torsion and predict unsalvageable testis. *Med Ultrason* 2022; 24(3):305-313.doi:10.11152/mu-3507.

44. Lee AS, Pohl HG, Rushton HG, Rana MS, Davis TD. Do healthcare disparities play a role in pediatric testicular torsion? - Analysis of a single large pediatric center. *J Pediatr Urol* 2022; 18(2):210.e211-210.e217.doi:10.1016/j.jpurol.2022.01.011.

45. Halevy D, Simanovsky N, Lev-Cohain N, Sosna J, Hiller N, Duvdevani M, et al. Parenchymal echotexture changes as a predictor of viability in testicular torsion. *Emerg Radiol* 2022; 29(2):359-363.doi:10.1007/s10140-021-02014-0.

46. Menzies-Wilson R, Folkard SS, Sevdalis N, Green JSA. Serious incidents in testicular torsion management in England, 2007-2019: optimizing individual and training factors are the key to improved outcomes. *BJU Int* 2022; 129(2):249-257.doi:10.1111/bju.15414.

47. Ali Benali N, Pradère B, Lannes F, Thi Dang V, Mauger de Varennes A, Gaillard C, et al. TORSAFUF - Surgical exploration for torsion of spermatic cord suspicion and risk factors for unnecessary surgery: Results of a French nationwide retrospective study on 2940 patients. *Prog Urol* 2022; 32(2):92-100.doi:10.1016/j.purol.2021.10.011.

48. Sazgar M, Montazer SH, Hosseininejad SM, Jahanian F, Rezaimehr B, Behbohaninia M, et al. Clinical Predictors of Testicular Torsion in Patients with Acute Scrotum; a Cross-Sectional Study. *Arch Acad Emerg Med* 2022; 10(1):e9.doi:10.22037/aaem.v10i1.1484.

49. Pinar U, Duquesne I, Lannes F, Bardet F, Kaulanjan K, Michiels C, et al. The Use of Doppler Ultrasound for Suspected Testicular Torsion: Lessons Learned from a 15-Year Multicentre Retrospective Study of 2922 Patients. *Eur Urol Focus* 2022; 8(1):105-111.doi:10.1016/j.euf.2021.02.011.

50. Hasan O, Mubarak M, Mohamed Jawad Alwedaie S, Baksh H, Alaradi H, Alarayedh A, et al. Ultrasound heterogeneity as an indicator of testicular salvage in testicular torsion: A single center experience. *Asian J Urol* 2022; 9(1):57-62.doi:10.1016/j.ajur.2021.05.006.

51. Vargas-Blasco C, Martin-Fumadó C, Benet-Travé J, Fuz F, Romero G, Autran M, et al. Clinical Safety Recommendations for Testicular Torsion: Analysis of 101 Claims Settled with Compensation in Spain and France. *Urol Int* 2022; 106(11):1095-1099.doi:10.1159/000526829.

52. Choi JB, Han KH, Lee Y, Ha US, Cho KJ, Kim JC, et al. The incidence of testicular torsion and testicular salvage rate in Korea over 10 years: A nationwide population-based study. *Investig Clin Urol* 2022; 63(4):448-454.doi:10.4111/icu.20220122.

53. Muraliharan JS, Jacob TJK, Bollu BK, Graf N, Sebastian T, Thomas G. Surgical management of the acute paediatric scrotum: a three-year single centre experience. *ANZ J Surg* 2021; 91(11):2514-2517.doi:10.1111/ans.17231.

54. Leone N, Morlacco A, D'Elia C, Amodeo A, Vecchio D, Tiscione D, et al. A retrospective multicentric analysis on testicular torsion: is there still something to learn? *Scand J Urol* 2021; 55(5):408-411.doi:10.1080/21681805.2021.1889026.

55. Pogorelić Z, Milanović K, Veršić AB, Pasini M, Divković D, Pavlović O, et al. Is there an increased incidence of orchiectomy in pediatric patients with acute testicular torsion during COVID-19 pandemic?-A retrospective multicenter study. *J Pediatr Urol* 2021; 17(4):479.e471-479.e476.doi:10.1016/j.jpurol.2021.04.017.

56. Holzman SA, Ahn JJ, Baker Z, Chuang KW, Copp HL, Davidson J, et al. A multicenter study of acute testicular torsion in the time of COVID-19. *J Pediatr Urol* 2021; 17(4):478.e471-478.e476.doi:10.1016/j.jpurol.2021.03.013.

57. Dupond-Athénor A, Peycelon M, Abbo O, Rod J, Haraux E, Scalabre A, et al. A multicenter review of undescended testis torsion: A plea for early management. *J Pediatr Urol* 2021; 17(2):191.e191-191.e196.doi:10.1016/j.jpurol.2020.12.004.

58. Zvizdic Z, Aganovic A, Milisic E, Jonuzi A, Zvizdic D, Vranic S. Duration of symptoms is the only predictor of testicular salvage following testicular torsion in children: A case-control study. *Am J Emerg Med* 2021; 41:197-200.doi:10.1016/j.ajem.2020.11.023.

59. Littman AR, Janssen KM, Tong L, Wu H, Wang MD, Blum E, et al. Did COVID-19 Affect Time to Presentation in the Setting of Pediatric Testicular Torsion? *Pediatr Emerg Care* 2021; 37(2):123-125.doi:10.1097/pec.0000000000002333.

60. Komarowska MD, Pawelczyk A, Matuszczak E, Dębek W, Hermanowicz A. Is Testicular Torsion a Real Problem in Pediatric Patients With Cryptorchidism? *Front Pediatr* 2020; 8:575741.doi:10.3389/fped.2020.575741.

61. Zheng WX, Hou GD, Zhang W, Wei D, Gao XL, Chen MH, et al. Establishment and internal validation of preoperative nomograms for predicting the possibility of testicular salvage in patients with testicular torsion. *Asian J Androl* 2021; 23(1):97-102.doi:10.4103/aja.aja_31_20.

62. Shields LBE, Daniels MW, Peppas DS, Rosenberg E. Testicular Torsion in Patients With Intellectual and Developmental Disabilities. *Glob Pediatr Health* 2021; 8:2333794x211059119.doi:10.1177/2333794x211059119.

63. Zhong H, Bi Y. Pediatric Trauma-Induced Testicular Torsion: A Surgical Emergency. *Urol Int* 2021; 105(3-4):221-224.doi:10.1159/000511747.

64. Yu C, Zhao J, Lu J, Wei Y, Jiang L, Zhao T, et al. Demographic, clinical, and socioeconomic factors associated with delayed diagnosis and management of pediatric testicular torsion in West China: a retrospective study of 301 cases in a single tertiary children's hospital. *BMC Pediatr* 2021; 21(1):553.doi:10.1186/s12887-021-03001-7.

65. Klinke M, Elrod J, Stiel C, Ghadban T, Wenskus J, Herrmann J, et al. The BAL-Score Almost Perfectly Predicts Testicular Torsion in Children: A Two-Center Cohort Study. *Front Pediatr* 2020; 8:601892.doi:10.3389/fped.2020.601892.

66. Vasconcelos-Castro S, Flor-de-Lima B, Campos JM, Soares-Oliveira M. Manual detorsion in testicular torsion: 5 years of experience at a single center. *J Pediatr Surg* 2020; 55(12):2728-2731.doi:10.1016/j.jpedsurg.2020.02.026.

67. Lim X, Angus MI, Panchalingam V, Chng KI, Choo CS, Chen Y, et al. Revisiting testicular torsion scores in an Asian healthcare system. *J Pediatr Urol* 2020; 16(6):821.e821-821.e827.doi:10.1016/j.jpurol.2020.09.023.

68. Göger YE, Özkent MS, Ünlü MZ, Kocaoğlu C, Madenci H, Pişkin MM. Evaluation of parental sociocultural background and education level in response to pediatric testis torsion. *J Pediatr Urol* 2020; 16(6):820.e821-820.e826.doi:10.1016/j.jpurol.2020.09.011.

69. Dias ACF, Maroccolo MVO, Ribeiro HP, Riccetto CLZ. Presentation delay, misdiagnosis, inter-hospital transfer times and surgical outcomes in testicular torsion: analysis of statewide case series from central Brazil. *Int Braz J Urol* 2020; 46(6):972-981.doi:10.1590/s1677-5538.Ibju.2019.0660.

70. Tian XM, Tan XH, Shi QL, Wen S, Lu P, Liu X, et al. Risk Factors for Testicular Atrophy in Children With Testicular Torsion Following Emergent Orchiopexy. *Front Pediatr* 2020; 8:584796.doi:10.3389/fped.2020.584796.

71. Merder E, Bozkurt M, Ariman A, Sezgin MA, Culha MG, Altunrende F. Comprehensive examination of haematological parameters of patients operated due to testicular torsion. *Andrologia* 2020; 52(9):e13674.doi:10.1111/and.13674.

72. Taskinen S, Mäkelä E, Raivio T. Effect of Pediatric Testicular Torsion on Testicular Function in the Short Term. *J Pediatr Surg* 2020; 55(8):1613-1615.doi:10.1016/j.jpedsurg.2019.10.023.

73. Kumar V, Matai P, Prabhu SP, Sundeep PT. Testicular Loss in Children Due to Incorrect Early Diagnosis of Torsion. *Clin Pediatr (Phila)* 2020; 59(4-5):436-438.doi:10.1177/0009922820903037.

74. Zhang X, Zhang J, Cai Z, Wang X, Lu W, Li H. Effect of unilateral testicular torsion at different ages on male fertility. *J Int Med Res* 2020; 48(4):300060520918792.doi:10.1177/0300060520918792.

75. Guo X, Sun L, Lei W, Li S, Guo H. Management of testicular torsion <360° in children: a single-center, retrospective study. *J Int Med Res* 2020; 48(4):300060519895861.doi:10.1177/0300060519895861.

76. Nandwani GM, Anwar A, Singh R, Stewart AB, Forster JA, Addla SK. Assessment of Age and Duration of Symptoms on Outcomes of Emergency Scrotal Exploration for Acute Scrotal Pain. *J Coll Physicians Surg Pak* 2020; 30(2):201-204.doi:10.29271/jcpsp.2020.02.201.

77. Tanaka K, Ogasawara Y, Nikai K, Yamada S, Fujiwara K, Okazaki T. Acute scrotum and testicular torsion in children: a retrospective study in a single institution. *J Pediatr Urol* 2020; 16(1):55-60.doi:10.1016/j.jpurol.2019.11.007.

78. Hosokawa T, Takahashi H, Tanami Y, Sato Y, Ishimaru T, Tanaka Y, et al. Diagnostic Accuracy of Ultrasound for the Directionality of Testicular Rotation and the Degree of Spermatic Cord Twist in Pediatric Patients With Testicular Torsion. *J Ultrasound Med* 2020; 39(1):119-126.doi:10.1002/jum.15084.

79. Obi AO, Okeke CJ, Ugwuidu EI. Acute testicular torsion: A critical analysis of presentation, management and outcome in southeast Nigeria. *Niger J Clin Pract* 2020; 23(11):1536-1541.doi:10.4103/njcp.njcp_188_20.

80. Feng S, Yang H, Lou Y, Ru W, Wang A, Liu W. Clinical Characteristics of Testicular Torsion and Identification of Predictors of Testicular Salvage in Children: A Retrospective Study in a Single Institution. *Urol Int* 2020; 104(11-12):878-883.doi:10.1159/000506236.

81. Overholt T, Jessop M, Barnard J, Al-Omar O. Pediatric testicular torsion: does patient transfer affect time to intervention or surgical outcomes at a rural tertiary care center? *BMC Urol* 2019; 19(1):39.doi:10.1186/s12894-019-0473-5.

82. Morin OA, Carr MG, Holcombe JM, Bhattacharya SD. Optimal Predictor of Gonadal Viability in Testicular Torsion: Time to Treat Versus Duration of Symptoms. *J Surg Res* 2019; 244:574-578.doi:10.1016/j.jss.2019.06.033.

83. Goetz J, Roewe R, Doolittle J, Roth E, Groth T, Mesrobian HG, et al. A comparison of clinical outcomes of acute testicular torsion between prepubertal and postpubertal males. *J Pediatr Urol* 2019; 15(6):610-616.doi:10.1016/j.jpurol.2019.07.020.

84. Gold DD, Lorber A, Levine H, Rosenberg S, Duvdevani M, Landau EH, et al. Door To Detorsion Time Determines Testicular Survival. *Urology* 2019; 133:211-215.doi:10.1016/j.urology.2019.08.003.

85. Zee RS, Bayne CE, Gomella PT, Pohl HG, Rushton HG, Davis TD. Implementation of the accelerated care of torsion pathway: a quality improvement initiative for testicular torsion. *J Pediatr Urol* 2019; 15(5):473-479.doi:10.1016/j.jpurol.2019.07.011.

86. Wang F, Mo Z. Clinical evaluation of testicular torsion presenting with acute abdominal pain in young males. *Asian J Urol* 2019; 6(4):368-372.doi:10.1016/j.ajur.2018.05.009.

87. He M, Zhang W, Sun N. Can haematologic parameters be used to predict testicular viability in testicular torsion? *Andrologia* 2019; 51(9):e13357.doi:10.1111/and.13357.

88. Afsarlar CE, Cakmakci E, Demir E, Guney G, Komut E, Elizondo R, et al. Novel prognostic grayscale ultrasonographic findings in the testis from a comprehensive analysis of pediatric patients with testicular torsion. *J Pediatr Urol* 2019; 15(5):480.e481-480.e487.doi:10.1016/j.jpurol.2019.08.002.

89. Romao RLP, Anderson KH, MacLellan D, Anderson P. Point-of-care influences orchiectomy rates in pediatric patients with testicular torsion. *J Pediatr Urol* 2019; 15(4):367.e361-367.e367.doi:10.1016/j.jpurol.2019.04.014.

90. Peeraully R, Jancauskaite M, Dawes S, Green S, Fraser N. Does the source of referral affect outcomes for paediatric testicular torsion? *Ann R Coll Surg Engl* 2019; 101(6):411-414.doi:10.1308/rcsann.2019.0045.

91. Chan EP, Wang PZT, Myslik F, Chen H, Dave S. Identifying systems delays in assessment, diagnosis, and operative management for testicular torsion in a single-payer health-care system. *J Pediatr Urol* 2019; 15(3):251.e251-251.e257.doi:10.1016/j.jpurol.2019.03.017.

92. Huang A, Delozier S, Lauderdale CJ, Zhao S, Clayton DB, Pope JCt, et al. Do repeat ultrasounds affect orchiectomy rate in patients with testicular torsion treated at a pediatric institution? *J Pediatr Urol* 2019; 15(2):179.e171-179.e175.doi:10.1016/j.jpurol.2018.12.002.

93. Jang JB, Ko YH, Choi JY, Song PH, Moon KH, Jung HC. Neutrophil-Lymphocyte Ratio Predicts Organ Salvage in Testicular Torsion with Marginal Diagnostic Delay. *World J Mens Health* 2019; 37(1):99-104.doi:10.5534/wjmh.180049.

94. Buicko JL, Satahoo SS, Rao KA, Sola JE, Neville HL. Disparities in pediatric gonadal torsion: Does gender, race and insurance status affect outcomes? *J Pediatr Surg* 2018; 53(7):1392-1395.doi:10.1016/j.jpedsurg.2018.02.046.

95. Yan Y, Chen S, Chen Z, Pei X, Zhou J, Xiao Y, et al. The applied value of medical history, physical examination, colour-Doppler ultrasonography and testis scintigraphy in the differential diagnosis of acute scrotum. *Andrologia* 2018.doi:10.1111/and.12973.

96. Yecies T, Bandari J, Schneck F, Cannon G. Direction of Rotation in Testicular Torsion and Identification of Predictors of Testicular Salvage. *Urology* 2018; 114:163-166.doi:10.1016/j.urology.2017.11.034.

97. Arevalo MK, Sheth KR, Menon VS, Ostrov L, Hennes H, Singla N, et al. Straight to the Operating Room: An Emergent Surgery Track for Acute Testicular Torsion Transfers. *J Pediatr* 2018; 192:178-183.doi:10.1016/j.jpeds.2017.09.009.

98. Manohar CS, Gupta A, Keshavamurthy R, Shivalingaiah M, Sharanbasappa BR, Singh VK. Evaluation of Testicular Workup for Ischemia and Suspected Torsion score in patients presenting with acute scrotum. *Urol Ann* 2018; 10(1):20-23.doi:10.4103/ua.Ua_35_17.

99. Frohlich LC, Paydar-Darian N, Cilento BG, Jr., Lee LK. Prospective Validation of Clinical Score for Males Presenting With an Acute Scrotum. *Acad Emerg Med* 2017; 24(12):1474-1482.doi:10.1111/acem.13295.

100. Demirbas A, Demir DO, Ersoy E, Kabar M, Ozcan S, Karagoz MA, et al. Should manual detorsion be a routine part of treatment in testicular torsion? *BMC Urol* 2017; 17(1):84.doi:10.1186/s12894-017-0276-5.

101. Preece J, Ching C, Yackey K, Jayanthi V, McLeod D, Alpert S, et al. Indicators and outcomes of transfer to tertiary pediatric hospitals for patients with testicular torsion. *J Pediatr Urol* 2017; 13(4):388.e381-388.e386.doi:10.1016/j.jpurol.2017.03.034.

102. Bayne CE, Villanueva J, Davis TD, Pohl HG, Rushton HG. Factors Associated with Delayed Presentation and Misdiagnosis of Testicular Torsion: A Case-Control Study. *J Pediatr* 2017; 186:200-204.doi:10.1016/j.jpeds.2017.03.037.

103. Nevo A, Mano R, Sivan B, Ben-Meir D. Missed Torsion of the Spermatic Cord: A Common yet Underreported Event. *Urology* 2017; 102:202-206.doi:10.1016/j.urology.2016.12.041.

104. Naouar S, Braiek S, El Kamel R. Testicular torsion in undescended testis: A persistent challenge. *Asian J Urol* 2017; 4(2):111-115.doi:10.1016/j.ajur.2016.05.007.

105. Samson P, Hartman C, Palmerola R, Rahman Z, Siev M, Palmer LS, et al. Ultrasonographic Assessment of Testicular Viability Using Heterogeneity Levels in Torsed Testicles. *J Urol* 2017; 197(3 Pt 2):925-930.doi:10.1016/j.juro.2016.09.112.

106. Bayne CE, Gomella PT, DiBianco JM, Davis TD, Pohl HG, Rushton HG. Testicular Torsion Presentation Trends before and after Pediatric Urology Subspecialty Certification. *J Urol* 2017; 197(2):507-515.doi:10.1016/j.juro.2016.09.090.

107. Sood A, Li H, Suson KD, Majumder K, Sedki M, Abdollah F, et al. Treatment patterns, testicular loss and disparities in inpatient surgical management of testicular torsion in boys: a population-based study 1998-2010. *BJU Int* 2016; 118(6):969-979.doi:10.1111/bju.13557.

108. Afsarlar CE, Ryan SL, Donel E, Baccam TH, Jones B, Chandwani B, et al. Standardized process to improve patient flow from the Emergency Room to the Operating Room for pediatric patients with testicular torsion. *J Pediatr Urol* 2016; 12(4):233.e231-234.doi:10.1016/j.jpurol.2016.04.019.

109. Ugwumba FO, Okoh AD, Echetabu KN. Acute and intermittent testicular torsion: Analysis of presentation, management, and outcome in South East, Nigeria. *Niger J Clin Pract* 2016; 19(3):407-410.doi:10.4103/1119-3077.179291.

110. Ayvaz OD, Celayir AC, Moralioglu S, Bosnali O, Pektas OZ, Pelin AK, et al. Four-year retrospective look for acute scrotal pathologies. *North Clin Istanb* 2015; 2(3):182-188.doi:10.14744/nci.2016.16768.

111. Johnston JW, Larsen P, El-Haddawi FH, Fancourt MW, Farrant GJ, Gilkison WT, et al. Time delays in presentation and treatment of acute scrotal pain in a provincial hospital. *ANZ J Surg* 2015; 85(5):330-333.doi:10.1111/ans.12601.

112. Ford KE, Cooper LL, Thenabadu S. Acute testicular pain in children: collaboration in timely management. *Eur J Emerg Med* 2015; 22(1):55-57.doi:10.1097/mej.0000000000000188.

113. Ramachandra P, Palazzi KL, Holmes NM, Marietti S. Factors influencing rate of testicular salvage in acute testicular torsion at a tertiary pediatric center. *West J Emerg Med* 2015; 16(1):190-194.doi:10.5811/westjem.2014.11.22495.

114. Güneş M, Umul M, Altok M, Akyüz M, İşoğlu CS, Uruç F, et al. Is it possible to distinguish testicular torsion from other causes of acute scrotum in patients who underwent scrotal exploration? A multi-center clinical trial. *Cent European J Urol* 2015; 68(2):252-256.doi:10.5173/ceju.2015.542.

115. Benedetto G, Nigro F, Bratti E, Tasca A. Modifications of echogenicity of the testis during acute torsion may be a predictive factor of organ damage? *Arch Ital Urol Androl* 2014; 86(4):371-372.doi:10.4081/aiua.2014.4.371.

116. Moslemi MK, Kamalimotlagh S. Evaluation of acute scrotum in our consecutive operated cases: a one-center study. *Int J Gen Med* 2014; 7:75-78.doi:10.2147/ijgm.S52413.

117. Lee SM, Huh JS, Baek M, Yoo KH, Min GE, Lee HL, et al. A nationwide epidemiological study of testicular torsion in Korea. *J Korean Med Sci* 2014; 29(12):1684-1687.doi:10.3346/jkms.2014.29.12.1684.

118. Yiee JH, Chang L, Kaplan A, Kwan L, Chung PJ, Litwin MS. Patterns of care in testicular torsion: influence of hospital transfer on testicular outcomes. *J Pediatr Urol* 2013; 9(6 Pt A):713-720.doi:10.1016/j.jpurol.2013.06.003.

119. Pogorelić Z, Mrklić I, Jurić I, Biočić M, Furlan D. Testicular torsion in the inguinal canal in children. *J Pediatr Urol* 2013; 9(6 Pt A):793-797.doi:10.1016/j.jpurol.2012.10.013.

120. Huang WY, Chen YF, Chang HC, Yang TK, Hsieh JT, Huang KH. The incidence rate and characteristics in patients with testicular torsion: a nationwide, population-based study. *Acta Paediatr* 2013; 102(8):e363-367.doi:10.1111/apa.12275.

121. Chen JS, Lin YM, Yang WH. Diurnal temperature change is associated with testicular torsion: a nationwide, population based study in Taiwan. *J Urol* 2013; 190(1):228-232.doi:10.1016/j.juro.2013.02.013.

122. Liang T, Metcalfe P, Sevcik W, Noga M. Retrospective review of diagnosis and treatment in children presenting to the pediatric department with acute scrotum. *AJR Am J Roentgenol* 2013; 200(5):W444-449.doi:10.2214/ajr.12.10036.

123. Barbosa JA, Tiseo BC, Barayan GA, Rosman BM, Torricelli FC, Passerotti CC, et al. Development and initial validation of a scoring system to diagnose testicular torsion in children. *J Urol* 2013; 189(5):1859-1864.doi:10.1016/j.juro.2012.10.056.

124. Boettcher M, Bergholz R, Krebs TF, Wenke K, Aronson DC. Clinical predictors of testicular torsion in children. *Urology* 2012; 79(3):670-674.doi:10.1016/j.urology.2011.10.041.

125. Yang C, Jr., Song B, Liu X, Wei GH, Lin T, He DW. Acute scrotum in children: an 18-year retrospective study. *Pediatr Emerg Care* 2011; 27(4):270-274.doi:10.1097/PEC.0b013e318213144e.

126. Molokwu CN, Somani BK, Goodman CM. Outcomes of scrotal exploration for acute scrotal pain suspicious of testicular torsion: a consecutive case series of 173 patients. *BJU Int* 2011; 107(6):990-993.doi:10.1111/j.1464-410X.2010.09557.x.

127. Bayne AP, Madden-Fuentes RJ, Jones EA, Cisek LJ, Gonzales ET, Jr., Reavis KM, et al. Factors associated with delayed treatment of acute testicular torsion-do demographics or interhospital transfer matter? *J Urol* 2010; 184(4 Suppl):1743-1747.doi:10.1016/j.juro.2010.03.073.

128. Beni-Israel T, Goldman M, Bar Chaim S, Kozer E. Clinical predictors for testicular torsion as seen in the pediatric ED. *Am J Emerg Med* 2010; 28(7):786-789.doi:10.1016/j.ajem.2009.03.025.

129. Chmelnik M, Schenk JP, Hinz U, Holland-Cunz S, Günther P. Testicular torsion: sonomorphological appearance as a predictor for testicular viability and outcome in neonates and children. *Pediatr Surg Int* 2010; 26(3):281-286.doi:10.1007/s00383-009-2534-4.

130. Kaye JD, Shapiro EY, Levitt SB, Friedman SC, Gitlin J, Freyle J, et al. Parenchymal echo texture predicts testicular salvage after torsion: potential impact on the need for emergent exploration. *J Urol* 2008; 180(4 Suppl):1733-1736.doi:10.1016/j.juro.2008.03.104.

131. Hayn MH, Herz DB, Bellinger MF, Schneck FX. Intermittent torsion of the spermatic cord portends an increased risk of acute testicular infarction. *J Urol* 2008; 180(4 Suppl):1729-1732.doi:10.1016/j.juro.2008.03.101.

132. Liu CC, Huang SP, Chou YH, Li CC, Wu MT, Huang CH, et al. Clinical presentation of acute scrotum in young males. *Kaohsiung J Med Sci* 2007; 23(6):281-286.doi:10.1016/s1607-551x(09)70410-3.

133. Mäkelä E, Lahdes-Vasama T, Rajakorpi H, Wikström S. A 19-year review of paediatric patients with acute scrotum. *Scand J Surg* 2007; 96(1):62-66.doi:10.1177/145749690709600112.

134. Al-Hunayan AA, Hanafy AM, Kehinde EO, Al-Awadi KA, Ali YM, Al-Twheed AR, et al. Testicular torsion: a perspective from the Middle East. *Med Princ Pract* 2004; 13(5):255-259.doi:10.1159/000079523.

135. Hegarty PK, Walsh E, Corcoran MO. Exploration of the acute scrotum: a retrospective analysis of 100 consecutive cases. *Ir J Med Sci* 2001; 170(3):181-182.doi:10.1007/bf03173885.

136. Rivers KK, Rivers EP, Stricker HJ, Lewis J, Urrunaga J, Karriem V. The clinical utility of serologic markers in the evaluation of the acute scrotum. *Acad Emerg Med* 2000; 7(9):1069-1072.doi:10.1111/j.1553-2712.2000.tb02103.x.

137. Barada JH, Weingarten JL, Cromie WJ. Testicular salvage and age-related delay in the presentation of testicular torsion. *J Urol* 1989; 142(3):746-748.doi:10.1016/s0022-5347(17)38875-4.

138. Anderson JB, Williamson RC. Testicular torsion in Bristol: a 25-year review. *Br J Surg* 1988; 75(10):988-992.doi:10.1002/bjs.1800751015.

139. Jones DJ, Macreadie D, Morgans BT. Testicular torsion in the armed services: twelve year review of 179 cases. *Br J Surg* 1986; 73(8):624-626.doi:10.1002/bjs.1800730813.

140. Udeh FN. Testicular torsion: Nigerian experience. *J Urol* 1985; 134(3):482-484.doi:10.1016/s0022-5347(17)47250-8.

141. Bartsch G, Frank S, Marberger H, Mikuz G. Testicular torsion: late results with special regard to fertility and endocrine function. *J Urol* 1980; 124(3):375-378.doi:10.1016/s0022-5347(17)55456-7.

142. Dimopoulos C, Giannopoulos A, Doïkas J, Ntoutsias A. Unusual presentation of testicular torsion. A review of 40 cases. *Eur Urol* 1976; 2(4):179-181.doi:10.1159/000471998.

143. Peretti M, Zampieri N, Bertozzi M, Bianchi F, Patanè S, Spigo V, et al. Mean Platelet Volume and Testicular Torsion: New Findings. *Urol J* 2019; 16(1):83-85.doi:10.22037/uj.v0i0.4042.

144. Tryfonas G, Violaki A, Tsikopoulos G, Avtzoglou P, Zioutis J, Limas C, et al. Late postoperative results in males treated for testicular torsion during childhood. *J Pediatr Surg* 1994; 29(4):553-556.doi:10.1016/0022-3468(94)90090-6.

145. Zhong HJ, Tang LF, Bi YL. Cryptorchid testicular torsion in children: characteristics and treatment outcomes. *Asian J Androl* 2021; 23(5):468-471.doi:10.4103/aja.aja_10_21.

146. Rampaul MS, Hosking SW. Testicular torsion: most delay occurs outside hospital. *Ann R Coll Surg Engl* 1998; 80(3):169-172

147. Qi L, Li-hui W, Jiao Z, Da Z, Ying-zhong F, Bao-ping Q, et al. Establishment of predictive model of testicular torsion in pediatric patients with acute scrotum. *Chinese Journal of Applied Clinical Pediatrics* 2013; 28(16):1273-1275.doi:10.3760/cma.j.issn.2095-428X.2013.16.021.

148. Chunfan L. 34 cases of testicular torsion in children. *Chinese Journal of Applied Clinical Pediatrics* 2003; 18(07):570-571.doi:10.3969/j.issn.1003-515X.2003.07.046.

149. Xiaoxiao Y, Jing J, Xin R, Wenjing C, Aiping Q, Zhenyong L, et al. Clinical study of peripubertal testicular torsion analysis and ultrasonic manifestations. *International Journal of Urology and Nephrology* 2025; 45(02):91-94.doi:10.3760/cma.j.cn431460-20230720-00071.

150. Lihan W, Danbo F, Baihua S, Shuo W, Liping X. Early diagnosis and treatment of 37 cases of testicular torsion in adolescent scrotal emergencies. *Chinese Journal of Emergency Medicine* 2010; 19(11):1212-1214.doi:10.3760/cma.j.issn.1671-0282.2010.11.026.

151. Xianjin W, Zhoujun S, Shan Z, Cunming Z, Zhaowei Z, Yuxuan W, et al. Diagnosis and treatment of testicular torsion ( report of 20 cases ). *International Journal of Urology and Nephrology* 2011; 31(01):1-4.doi:10.3760/cma.j.issn.1673-4416.2011.01.001.

152. Wenxuan C, Zhe C, Yingwu W, Liming L. Analysis of diagnosis and treatment of 60 cases of testicular torsion. *Chinese Journal of Postgraduates of Medicine* 2010; 33(23):59-61.doi: 10.3760/cma.j.issn.1673-4904.2010.23.026.

153. Qun L, Changwei J, Gutian Z, Huibo L, Shiwei Z, Xiaogong L, et al. Clinical analysis of 49 cases with testicular torsion. *Chinese Journal of Surgery* 2015; 53(08):599-602.doi:10.3760/cma.j.issn.0529-5815.2015.08.009.

154. Guofang Z, Rui W, Baoxin J. Clinical analysis of 45 cases of testicular torsion. *Chinese Journal of Practical Medicine* 2004; 31(16):17-17

155. Rujian Z, Fanghu S, Guanfu W, Hongyuan Y, Tianji W. Report of 42 cases of testicular torsion. *Chinese Journal of Urology* 2008; 29(10):716-716

156. Shicheng Y, Peter C, Haiyang W, Liwei X, Guoqing D, Weiping Z, et al. Analysis of diagnosis and treatment of 36 cases of testicular torsion. *Chinese Journal of Surgery* 2013; 51(10):949-950

157. Jianyong G. Report of 30 cases of testicular torsion. *Chinese Journal of Primary Medicine and Pharmacy* 2008; 15(03):486-486.doi:10.3760/cma.j.issn.1008-6706.2008.03.091.

158. Yangguo S, Xinjun Y. Clinical analysis of 25 cases of testicular torsion. *Chinese Journal of Practical Medicine* 2011; 38(03):71-72.doi:10.3760/cma.j.issn.1674-4756.2011.03.035.

159. Wenlong M, Xiaoyun Z. Report of 19 Cases of Testicular Torsion. *Chinese Journal of Urology* 2006; 27(02):113-113

160. Yongbiao Z. Experience with the diagnosis and treatment of 16 cases of testicular torsion. *Chinese Journal of Practical Medicine* 2007; 34(14):40-41

161. Chengshang H. Clinical Analysis of 27 Cases of Testicular Torsion in Children. *Chinese Journal of Practical Medicine* 2012; 39(13):81-82

162. Jianjun H, Yaowang Z, Yifu C, Tianqu H, Liucheng P. Clinical diagnosis and treatment of 108 cases of testicular torsion and multivariate regression analysis. *International Journal of Urology and Nephrology* 2024; 44(03):521-525.doi:10.3760/cma.j.cn431460-20220822-00129.

163. Xiaofeng C, Wei Z, Bihua D, Wanglong D, Dong W, Yihua Z. Clinical Features, Diagnosis, and Treatment of Testicular Torsion in 85 Cases. *International Journal of Urology and Nephrology* 2021; 41(02):345-347

164. Dezhi L, Jing H. Diagnosis and treatment of testicular torsion in 56 children. *Chinese Pediatric Emergency Medicine* 2007; 14(03):263-264.doi:10.3760/cma.j.issn.1673-4912.2007.03.035.

165. Tianqi Z. Report of 18 cases of testicular torsion. *Chinese Journal of Urology* 2001; 22(02):98-99

166. Гулиев БГ, Шевнин МВ, Васильева ЕИ. ПЕРЕКРУТ ЯИЧКА У ВЗРОСЛЫХ: ОПЫТ СТАЦИОНАРА. *Медицина: теория и практика* 2023; 8(4):176-182.doi:10.56871/MTP.2023.29.98.027.

167. Murithi J, Mwachi A, Abdalla R, Chavda S. Management and outcome of Testicular Torsion. *Annals of African Surgery* 2017; 14(2)

168. Ibingira C. Management of Testicular torsion in Mulago Hospital over a 5-year period. *East and Central African Journal of Surgery* 2001; 6(2)

169. Derbew M, Laytin A. Testicular torsion in Ethiopia: a case series and systematic review of the sub-saharan african literature. *East and Central African Journal of Surgery* 2015; 20(2):17-23

170. Gnassingbe K, Akakpo-Numado K, Songne-G B, Anoukoum T, Kao M, Tékou H. Testicular Torsion in Children. *African Journal of Urology* 2009; 15(4)

171. Muguti G, Kalgudi R. Torsion of the testis: review of clinical experience in Zimbabwe. *The Central African Journal of Medicine* 1994; 40(5):119-122

172. Okorie CO. Unilateral testicular torsion with necrotic outcome: dilemmas of surgical timing. *Urology* 2011; 78(6):1232-1234.doi:10.1016/j.urology.2011.08.059.

173. Файзулоев ДА, Шерназаров И, Ахмедов Р. Отдалённые результаты хирургического лечения и наблюдения детей с перекрутом яичка. *Вестник Авиценны* 2009; (3 (40))

174. Карташев В, Румянцева Г, Аврасин А. Опыт лечения перекрута яичка у детей. *Урологические ведомости* 2019; (Спецвыпуск):46-47

175. Комарова СЮ, Цап Н, Чукреев В. Особенности консервативной и оперативной тактики при перекруте яичка. *Детская хирургия* 2016; 20(4):185-188

176. Доронин М, Дуб И, Стриго А, Дубовский И, Мурашко Ю, Щетко Г, et al. Вопросы диагностики перекрута яичка в вооруженных силах Республики Беларусь. 2024;
