# Supplementary material for: Are we still too late to preserve the testes? A global survey of delayed consultation and risk factors for testicular torsion: a systematic review and meta-analysis
Source: Front Reprod Health. 2026 Feb 24;8:1735652. doi: 10.3389/frph.2026.1735652 (PMC12971663; doi:10.3389/frph.2026.1735652)
Supplement: Supplementary file 4 [file Datasheet1.pdf]

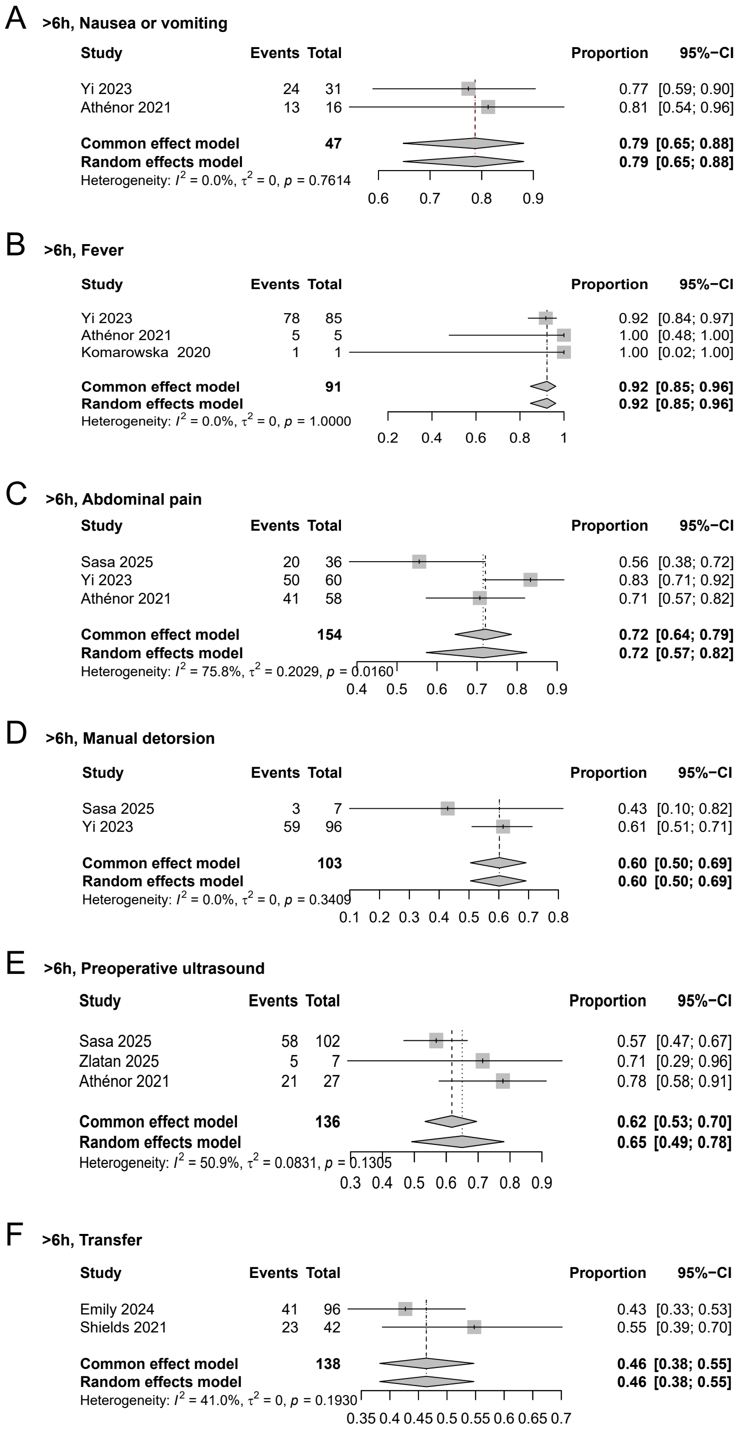
A
>6h, Nausea or vomiting
Study Events Total Proportion 95%−CI
Yi 2023 24 31 0.77 [0.59; 0.90]
Athénor 2021 13 16 0.81 [0.54; 0.96]
Common effect model 47 0.79 [0.65; 0.88]
Random effects model 0.79 [0.65; 0.88]
Heterogeneity: I^2 = 0.0%, τ^2 = 0, p = 0.7614
0.6 0.7 0.8 0.9
B
>6h, Fever
Study Events Total Proportion 95%−CI
Yi 2023 78 85 0.92 [0.84; 0.97]
Athénor 2021 5 5 1.00 [0.48; 1.00]
Komarowska 2020 1 1 1.00 [0.02; 1.00]
Common effect model 91 0.92 [0.85; 0.96]
Random effects model 0.92 [0.85; 0.96]
Heterogeneity: I^2 = 0.0%, τ^2 = 0, p = 1.0000
0.2 0.4 0.6 0.8 1
C
>6h, Abdominal pain
Study Events Total Proportion 95%−CI
Sasa 2025 20 36 0.56 [0.38; 0.72]
Yi 2023 50 60 0.83 [0.71; 0.92]
Athénor 2021 41 58 0.71 [0.57; 0.82]
Common effect model 154 0.72 [0.64; 0.79]
Random effects model 0.72 [0.57; 0.82]
Heterogeneity: I^2 = 75.8%, τ^2 = 0.2029, p = 0.0160
0.4 0.5 0.6 0.7 0.8 0.9
D
>6h, Manual detorsion
Study Events Total Proportion 95%−CI
Sasa 2025 3 7 0.43 [0.10; 0.82]
Yi 2023 59 96 0.61 [0.51; 0.71]
Common effect model 103 0.60 [0.50; 0.69]
Random effects model 0.60 [0.50; 0.69]
Heterogeneity: I^2 = 0.0%, τ^2 = 0, p = 0.3409
0.1 0.2 0.3 0.4 0.5 0.6 0.7 0.8
E
>6h, Preoperative ultrasound
Study Events Total Proportion 95%−CI
Sasa 2025 58 102 0.57 [0.47; 0.67]
Zlatan 2025 5 7 0.71 [0.29; 0.96]
Athénor 2021 21 27 0.78 [0.58; 0.91]
Common effect model 136 0.62 [0.53; 0.70]
Random effects model 0.65 [0.49; 0.78]
Heterogeneity: I^2 = 50.9%, τ^2 = 0.0831, p = 0.1305
0.3 0.4 0.5 0.6 0.7 0.8 0.9
F
>6h, Transfer
Study Events Total Proportion 95%−CI
Emily 2024 41 96 0.43 [0.33; 0.53]
Shields 2021 23 42 0.55 [0.39; 0.70]
Common effect model 138 0.46 [0.38; 0.55]
Random effects model 0.46 [0.38; 0.55]
Heterogeneity: I^2 = 41.0%, τ^2 = 0, p = 0.1930
0.35 0.4 0.45 0.5 0.55 0.6 0.65 0.7
