# Supplementary material for: Are we still too late to preserve the testes? A global survey of delayed consultation and risk factors for testicular torsion: a systematic review and meta-analysis
Source: Front Reprod Health. 2026 Feb 24;8:1735652. doi: 10.3389/frph.2026.1735652 (PMC12971663; doi:10.3389/frph.2026.1735652)
Supplement: Supplementary file 5 [file Datasheet2.pdf]

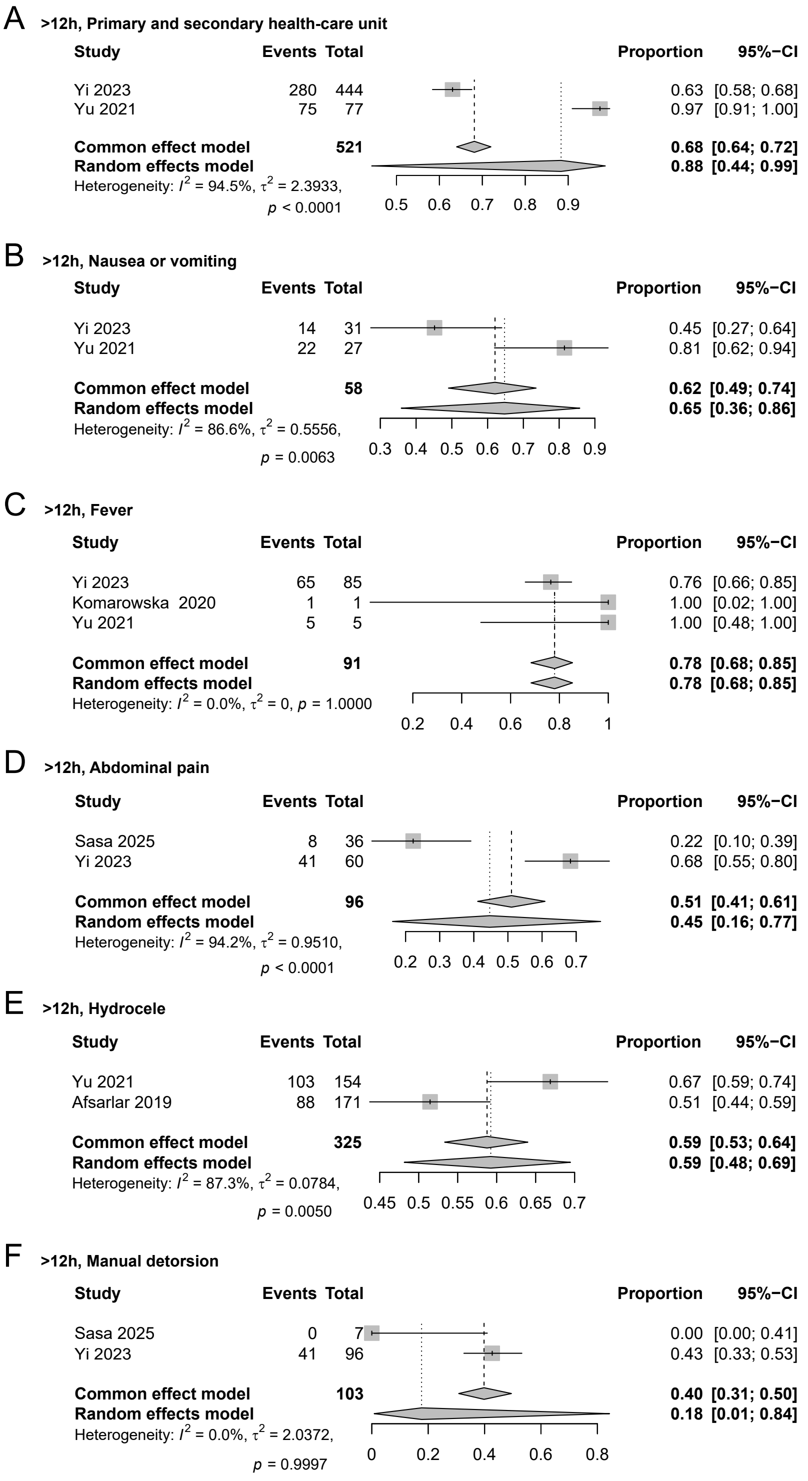
A
>12h, Primary and secondary health-care unit
Study Events Total Proportion 95%−CI
Yi 2023 280 444 0.63 [0.58; 0.68]
Yu 2021 75 77 0.97 [0.91; 1.00]
Common effect model 521 0.68 [0.64; 0.72]
Random effects model 0.88 [0.44; 0.99]
Heterogeneity: I^2 = 94.5%, τ^2 = 2.3933, p < 0.0001
0.5 0.6 0.7 0.8 0.9
B
>12h, Nausea or vomiting
Study Events Total Proportion 95%−CI
Yi 2023 14 31 0.45 [0.27; 0.64]
Yu 2021 22 27 0.81 [0.62; 0.94]
Common effect model 58 0.62 [0.49; 0.74]
Random effects model 0.65 [0.36; 0.86]
Heterogeneity: I^2 = 86.6%, τ^2 = 0.5556, p = 0.0063
0.3 0.4 0.5 0.6 0.7 0.8 0.9
C
>12h, Fever
Study Events Total Proportion 95%−CI
Yi 2023 65 85 0.76 [0.66; 0.85]
Komarowska 2020 1 1 1.00 [0.02; 1.00]
Yu 2021 5 5 1.00 [0.48; 1.00]
Common effect model 91 0.78 [0.68; 0.85]
Random effects model 0.78 [0.68; 0.85]
Heterogeneity: I^2 = 0.0%, τ^2 = 0, p = 1.0000
0.2 0.4 0.6 0.8 1
D
>12h, Abdominal pain
Study Events Total Proportion 95%−CI
Sasa 2025 8 36 0.22 [0.10; 0.39]
Yi 2023 41 60 0.68 [0.55; 0.80]
Common effect model 96 0.51 [0.41; 0.61]
Random effects model 0.45 [0.16; 0.77]
Heterogeneity: I^2 = 94.2%, τ^2 = 0.9510, p < 0.0001
0.2 0.3 0.4 0.5 0.6 0.7
E
>12h, Hydrocele
Study Events Total Proportion 95%−CI
Yu 2021 103 154 0.67 [0.59; 0.74]
Afsarlar 2019 88 171 0.51 [0.44; 0.59]
Common effect model 325 0.59 [0.53; 0.64]
Random effects model 0.59 [0.48; 0.69]
Heterogeneity: I^2 = 87.3%, τ^2 = 0.0784, p = 0.0050
0.45 0.5 0.55 0.6 0.65 0.7
F
>12h, Manual detorsion
Study Events Total Proportion 95%−CI
Sasa 2025 0 7 0.00 [0.00; 0.41]
Yi 2023 41 96 0.43 [0.33; 0.53]
Common effect model 103 0.40 [0.31; 0.50]
Random effects model 0.18 [0.01; 0.84]
Heterogeneity: I^2 = 0.0%, τ^2 = 2.0372, p = 0.9997
0 0.2 0.4 0.6 0.8
