# Supplementary figures and images for: Are we still too late to preserve the testes? A global survey of delayed consultation and risk factors for testicular torsion: a systematic review and meta-analysis
Source: Front Reprod Health. 2026 Feb 24;8:1735652. doi: 10.3389/frph.2026.1735652 (PMC12971663; doi:10.3389/frph.2026.1735652)

G

&gt;12h, Misdiagnosis

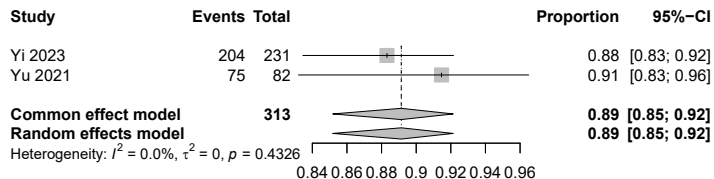

H

&gt;12h, During pandemic

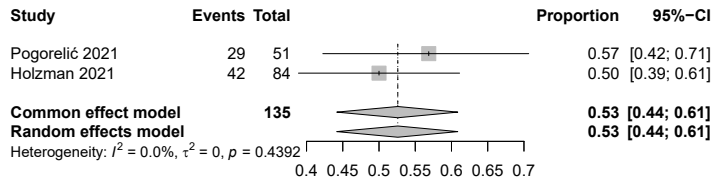

Supplement: Supplementary file 6 [file Datasheet3.pdf]

## G >24h, Misdiagnosis

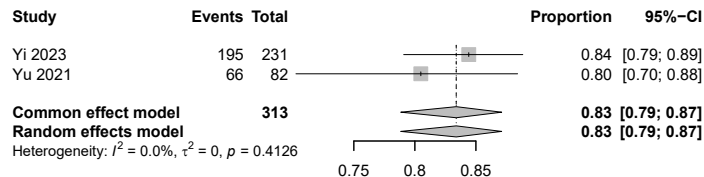

## H >24h, During pandemic

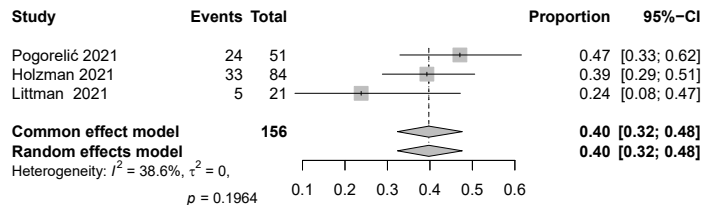

## I >24h, Transfer

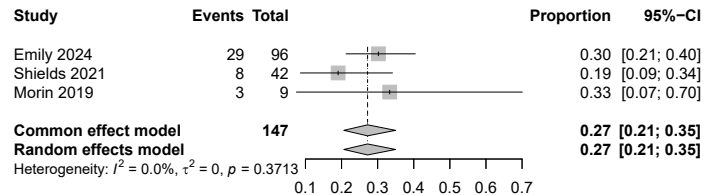

Supplement: Supplementary file 8 [file Datasheet5.pdf]

F >24h, Hydrocele

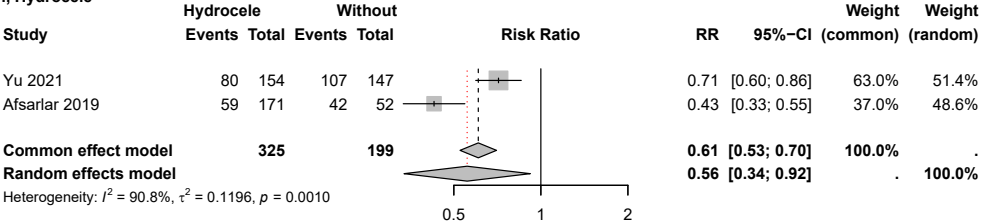

G >24h, Misdiagnosis

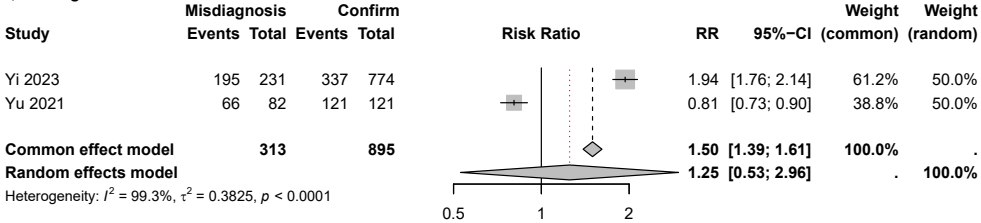

H >24h, During pandemic

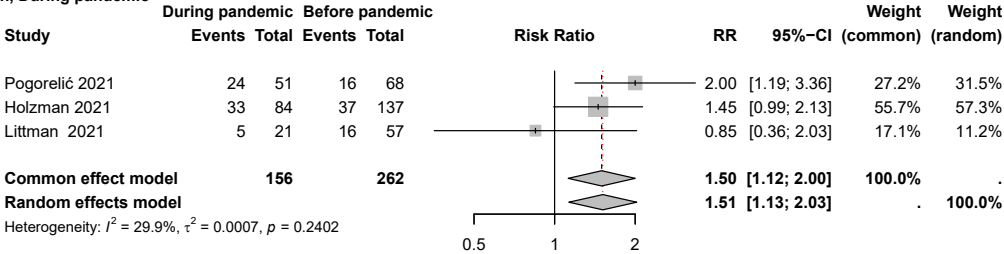

I >24h, Transfer

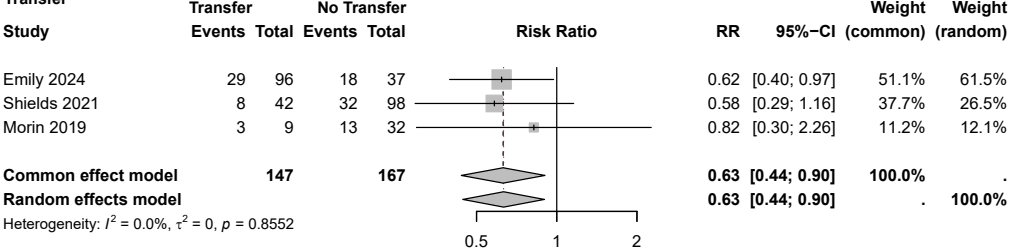

Supplement: Supplementary file 13 [file Datasheet10.pdf]

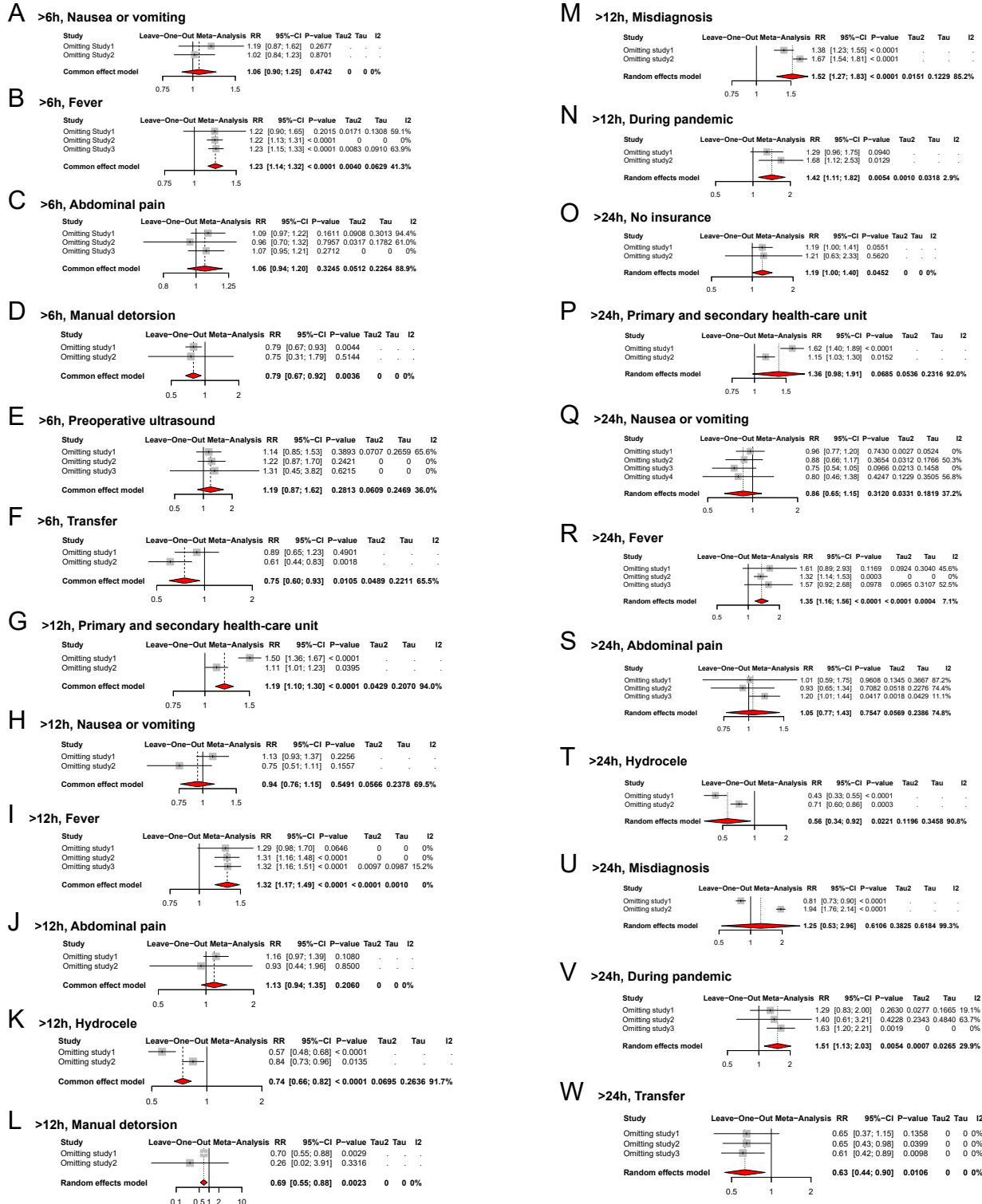

Supplement: Supplementary file 15 [file Datasheet12.pdf]

A

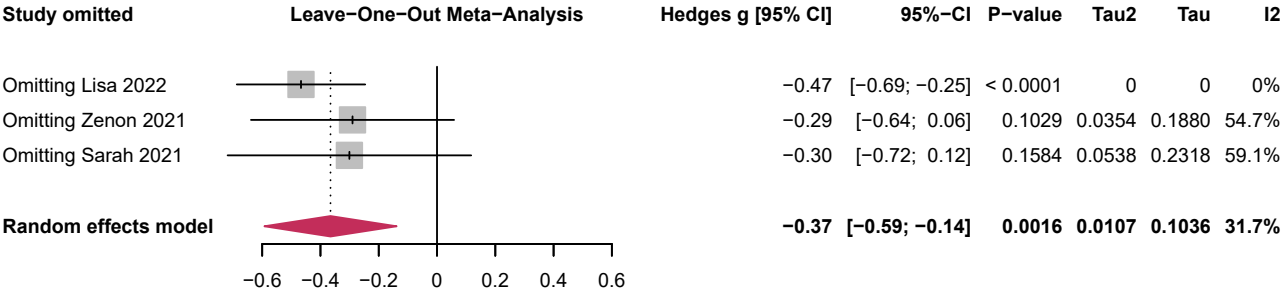

B

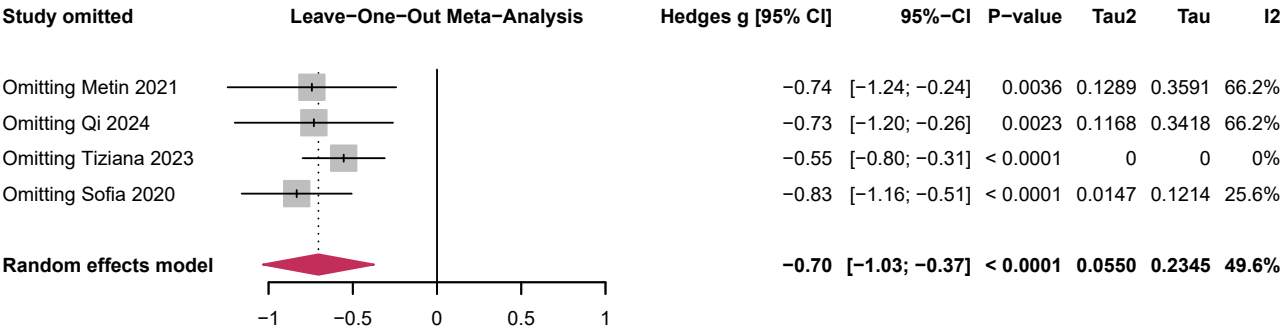

C

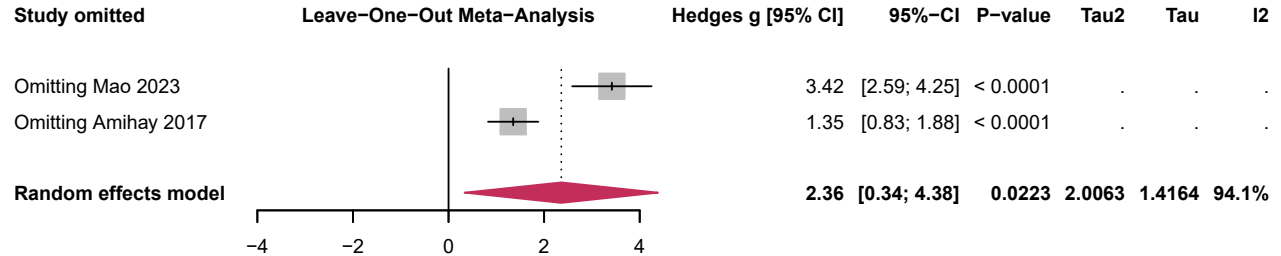

D

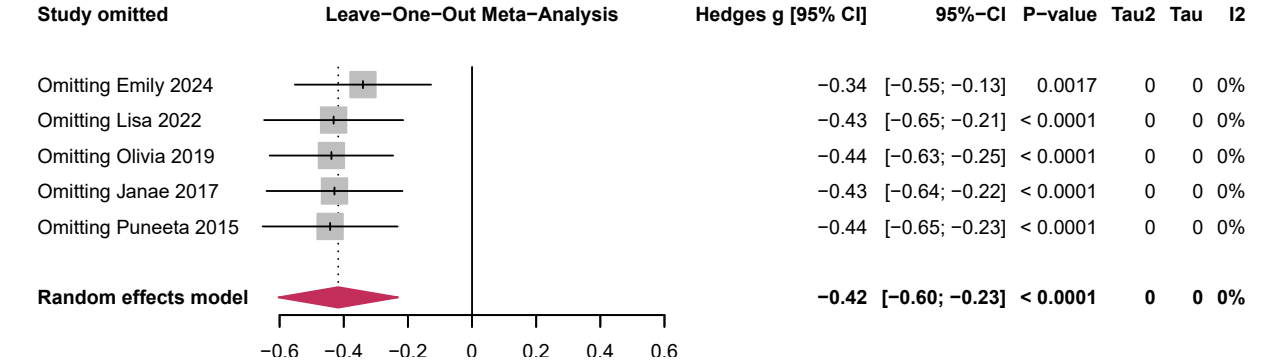

Supplement: Supplementary file 16 [file Datasheet13.pdf]
