# Supplementary material for: Are we still too late to preserve the testes? A global survey of delayed consultation and risk factors for testicular torsion: a systematic review and meta-analysis
Source: Front Reprod Health. 2026 Feb 24;8:1735652. doi: 10.3389/frph.2026.1735652 (PMC12971663; doi:10.3389/frph.2026.1735652)
Supplement: Supplementary file 7 [file Datasheet4.pdf]

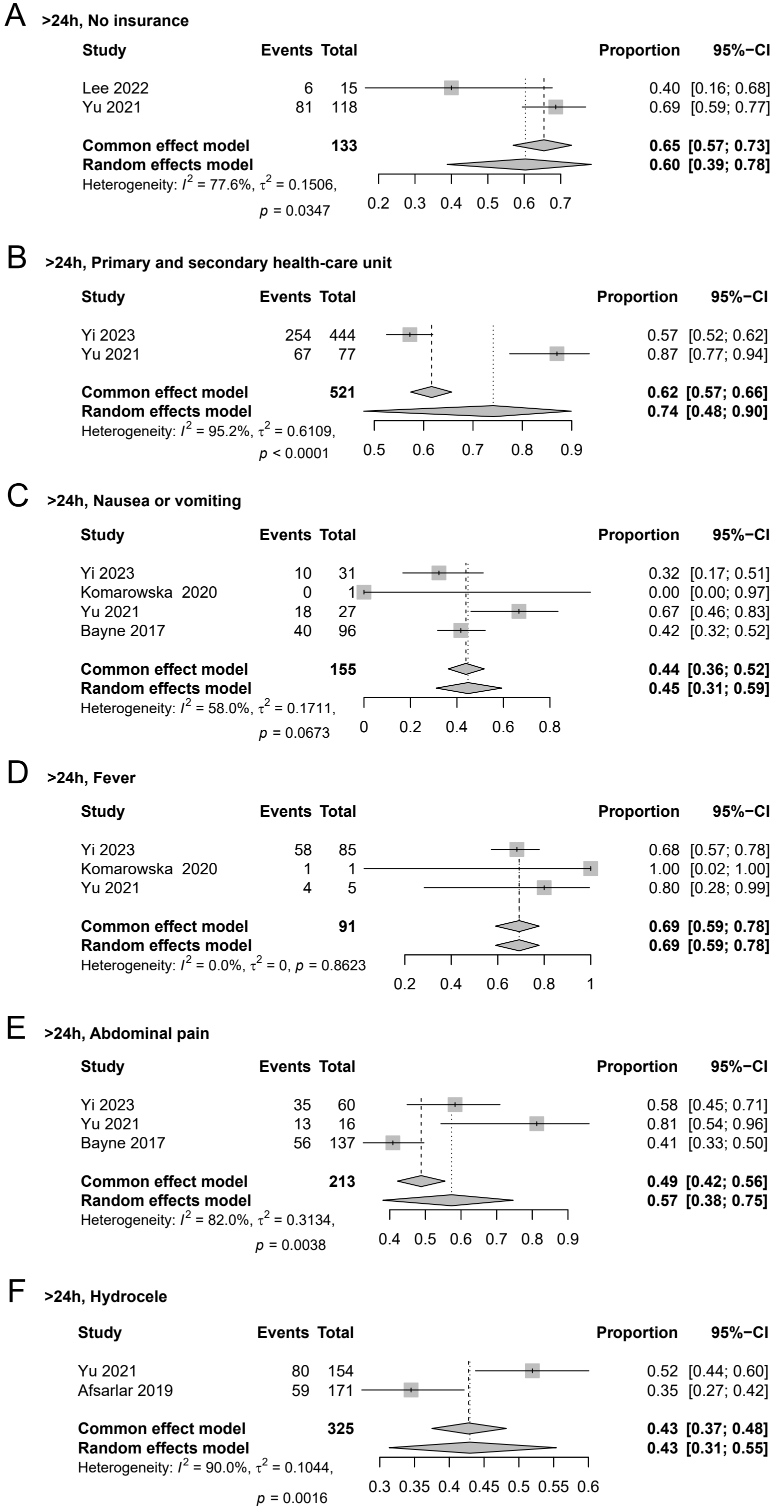
A >24h, No insurance
Study Events Total Proportion 95%−CI
Lee 2022 6 15 0.40 [0.16; 0.68]
Yu 2021 81 118 0.69 [0.59; 0.77]
Common effect model 133 0.65 [0.57; 0.73]
Random effects model 0.60 [0.39; 0.78]
Heterogeneity: I² = 77.6%, τ² = 0.1506, p = 0.0347
0.2 0.3 0.4 0.5 0.6 0.7
B >24h, Primary and secondary health-care unit
Study Events Total Proportion 95%−CI
Yi 2023 254 444 0.57 [0.52; 0.62]
Yu 2021 67 77 0.87 [0.77; 0.94]
Common effect model 521 0.62 [0.57; 0.66]
Random effects model 0.74 [0.48; 0.90]
Heterogeneity: I² = 95.2%, τ² = 0.6109, p < 0.0001
0.5 0.6 0.7 0.8 0.9
C >24h, Nausea or vomiting
Study Events Total Proportion 95%−CI
Yi 2023 10 31 0.32 [0.17; 0.51]
Komarowska 2020 0 1 0.00 [0.00; 0.97]
Yu 2021 18 27 0.67 [0.46; 0.83]
Bayne 2017 40 96 0.42 [0.32; 0.52]
Common effect model 155 0.44 [0.36; 0.52]
Random effects model 0.45 [0.31; 0.59]
Heterogeneity: I² = 58.0%, τ² = 0.1711, p = 0.0673
0 0.2 0.4 0.6 0.8
D >24h, Fever
Study Events Total Proportion 95%−CI
Yi 2023 58 85 0.68 [0.57; 0.78]
Komarowska 2020 1 1 1.00 [0.02; 1.00]
Yu 2021 4 5 0.80 [0.28; 0.99]
Common effect model 91 0.69 [0.59; 0.78]
Random effects model 0.69 [0.59; 0.78]
Heterogeneity: I² = 0.0%, τ² = 0, p = 0.8623
0.2 0.4 0.6 0.8 1
E >24h, Abdominal pain
Study Events Total Proportion 95%−CI
Yi 2023 35 60 0.58 [0.45; 0.71]
Yu 2021 13 16 0.81 [0.54; 0.96]
Bayne 2017 56 137 0.41 [0.33; 0.50]
Common effect model 213 0.49 [0.42; 0.56]
Random effects model 0.57 [0.38; 0.75]
Heterogeneity: I² = 82.0%, τ² = 0.3134, p = 0.0038
0.4 0.5 0.6 0.7 0.8 0.9
F >24h, Hydrocele
Study Events Total Proportion 95%−CI
Yu 2021 80 154 0.52 [0.44; 0.60]
Afsarlar 2019 59 171 0.35 [0.27; 0.42]
Common effect model 325 0.43 [0.37; 0.48]
Random effects model 0.43 [0.31; 0.55]
Heterogeneity: I² = 90.0%, τ² = 0.1044, p = 0.0016
0.3 0.35 0.4 0.45 0.5 0.55 0.6
