# Supplementary material for: Are we still too late to preserve the testes? A global survey of delayed consultation and risk factors for testicular torsion: a systematic review and meta-analysis
Source: Front Reprod Health. 2026 Feb 24;8:1735652. doi: 10.3389/frph.2026.1735652 (PMC12971663; doi:10.3389/frph.2026.1735652)
Supplement: Supplementary file 9 [file Datasheet6.pdf]

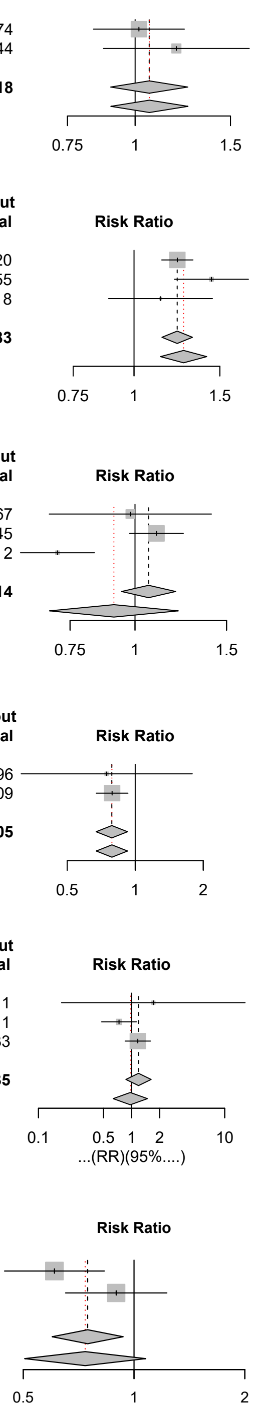

A **>6h, Nausea or vomiting**

Study	Nausea or vomiting Events	Nausea or vomiting Total	Without Events	Without Total	RR	95%−CI	Weight (common)	Weight (random)
Yi 2023	24	31	742	974	1.02	[0.84; 1.23]	74.1%	72.0%
Athénor 2021	13	16	30	44	1.19	[0.87; 1.62]	25.9%	28.0%
**Common effect model**		**47**		**1018**	**1.06**	**[0.90; 1.25]**	**100.0%**	**.**
**Random effects model**					**1.06**	**[0.90; 1.25]**	**.**	**100.0%**

Heterogeneity: $I^2 = 0.0\%$, $\tau^2 = 0$, $p = 0.3931$

B **>6h, Fever**

Study	Fever Events	Fever Total	Without Events	Without Total	RR	95%−CI	Weight (common)	Weight (random)
Yi 2023	78	85	688	920	1.23	[1.14; 1.32]	92.0%	58.0%
Athénor 2021	5	5	38	55	1.44	[1.21; 1.72]	5.9%	26.2%
Komarowska 2020	1	1	7	8	1.13	[0.89; 1.45]	2.2%	15.9%
**Common effect model**		**91**		**983**	**1.23**	**[1.14; 1.32]**	**100.0%**	**.**
**Random effects model**					**1.26**	**[1.13; 1.41]**	**.**	**100.0%**

Heterogeneity: $I^2 = 41.3\%$, $\tau^2 = 0.0040$, $p = 0.1821$

C **>6h, Abdominal pain**

Study	Abdominal pain Events	Abdominal pain Total	Without Events	Without Total	RR	95%−CI	Weight (common)	Weight (random)
Sasa 2025	20	36	38	67	0.98	[0.68; 1.40]	22.7%	25.0%
Yi 2023	50	60	716	945	1.10	[0.98; 1.24]	73.2%	38.6%
Athénor 2021	41	58	2	2	0.71	[0.60; 0.84]	4.1%	36.4%
**Common effect model**		**154**		**1014**	**1.06**	**[0.94; 1.20]**	**100.0%**	**.**
**Random effects model**					**0.91**	**[0.68; 1.21]**	**.**	**100.0%**

Heterogeneity: $I^2 = 88.9\%$, $\tau^2 = 0.0512$, $p = 0.0001$

D **>6h, Manual detorsion**

Study	Manual detorsion Events	Manual detorsion Total	Without Events	Without Total	RR	95%−CI	Weight (common)	Weight (random)
Sasa 2025	3	7	55	96	0.75	[0.31; 1.79]	5.2%	3.3%
Yi 2023	59	96	707	909	0.79	[0.67; 0.93]	94.8%	96.7%
**Common effect model**		**103**		**1005**	**0.79**	**[0.67; 0.92]**	**100.0%**	**.**
**Random effects model**					**0.79**	**[0.67; 0.93]**	**.**	**100.0%**

Heterogeneity: $I^2 = 0.0\%$, $\tau^2 = 0$, $p = 0.9037$

E **>6h, Preoperative ultrasound**

Study	Preoperative ultrasound Events	Preoperative ultrasound Total	Without Events	Without Total	RR	95%−CI	Weight (common)	Weight (random)
Sasa 2025	58	102	0	1	1.71	[0.18; 16.56]	4.2%	3.3%
Zlatan 2025	5	7	1	1	0.73	[0.48; 1.13]	10.4%	42.7%
Athénor 2021	21	27	22	33	1.17	[0.85; 1.60]	85.4%	53.9%
**Common effect model**		**136**		**35**	**1.19**	**[0.87; 1.62]**	**100.0%**	**.**
**Random effects model**					**0.97**	**[0.63; 1.48]**	**.**	**100.0%**

Heterogeneity: $I^2 = 36.0\%$, $\tau^2 = 0.0609$, $p = 0.2098$

F **>6h, Transfer**

Study	Transfer Events	Transfer Total	No Transfer Events	No Transfer Total	RR	95%−CI	Weight (common)	Weight (random)
Emily 2024	41	96	26	37	0.61	[0.44; 0.83]	51.0%	50.2%
Shields 2021	23	42	60	98	0.89	[0.65; 1.23]	49.0%	49.8%
**Common effect model**		**138**		**135**	**0.75**	**[0.60; 0.93]**	**100.0%**	**.**
**Random effects model**					**0.74**	**[0.50; 1.08]**	**.**	**100.0%**

Heterogeneity: $I^2 = 65.5\%$, $\tau^2 = 0.0489$, $p = 0.0887$
