# Supplementary material for: Are we still too late to preserve the testes? A global survey of delayed consultation and risk factors for testicular torsion: a systematic review and meta-analysis
Source: Front Reprod Health. 2026 Feb 24;8:1735652. doi: 10.3389/frph.2026.1735652 (PMC12971663; doi:10.3389/frph.2026.1735652)
Supplement: Supplementary file 10 [file Datasheet7.pdf]

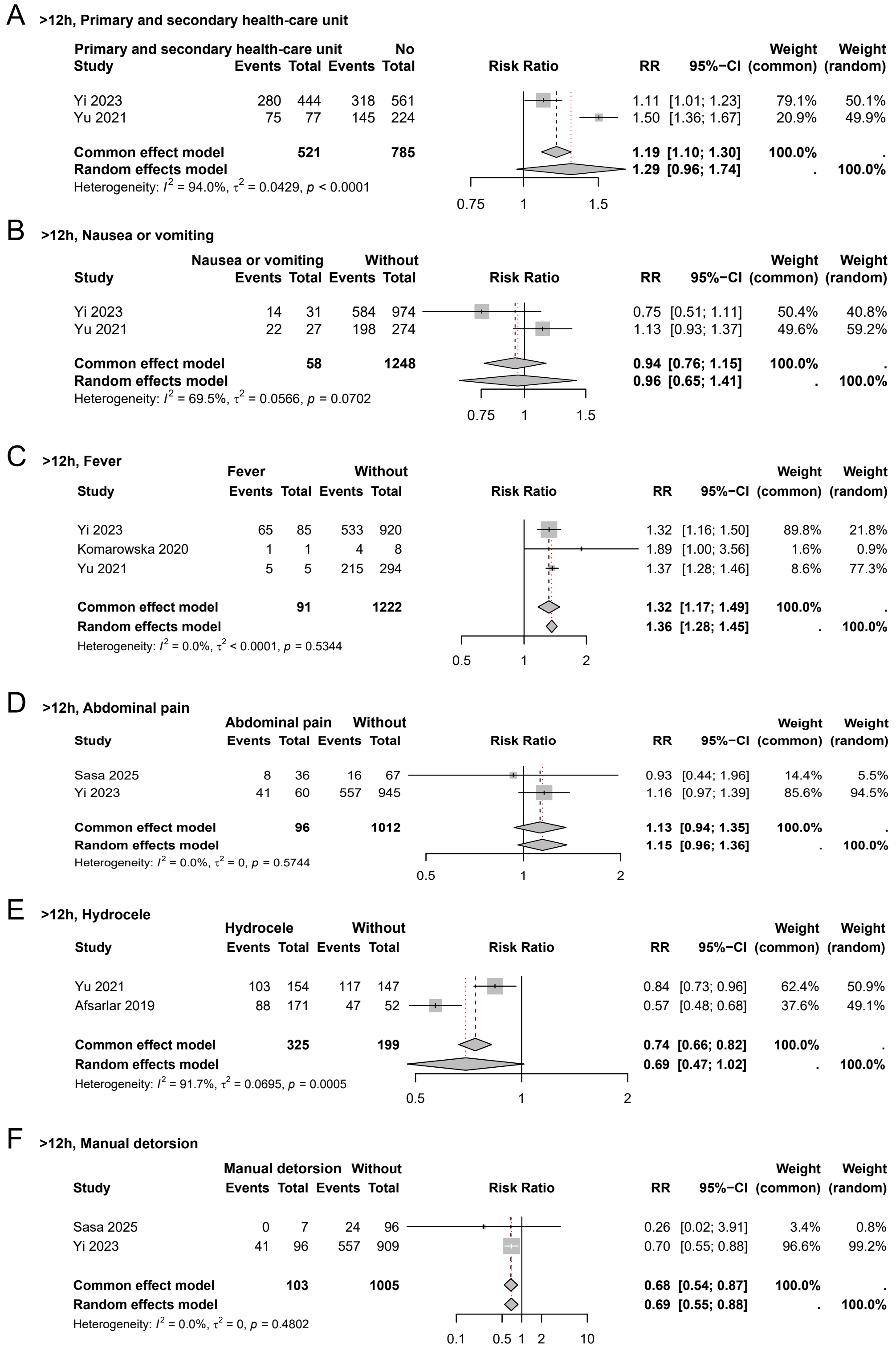
A
>12h, Primary and secondary health-care unit
Primary and secondary health-care unit
No
Study
Events Total Events Total
Risk Ratio
RR 95%−CI
Weight (common)
Weight (random)
Yi 2023 280 444 318 561 1.11 [1.01; 1.23] 79.1% 50.1%
Yu 2021 75 77 145 224 1.50 [1.36; 1.67] 20.9% 49.9%
Common effect model 521 785 1.19 [1.10; 1.30] 100.0% .
Random effects model 1.29 [0.96; 1.74] . 100.0%
Heterogeneity: I2 = 94.0%, τ2 = 0.0429, p < 0.0001
0.75
1
1.5
B
>12h, Nausea or vomiting
Nausea or vomiting
Without
Study
Events Total Events Total
Risk Ratio
RR 95%−CI
Weight (common)
Weight (random)
Yi 2023 14 31 584 974 0.75 [0.51; 1.11] 50.4% 40.8%
Yu 2021 22 27 198 274 1.13 [0.93; 1.37] 49.6% 59.2%
Common effect model 58 1248 0.94 [0.76; 1.15] 100.0% .
Random effects model 0.96 [0.65; 1.41] . 100.0%
Heterogeneity: I2 = 69.5%, τ2 = 0.0566, p = 0.0702
0.75
1
1.5
C
>12h, Fever
Fever
Without
Study
Events Total Events Total
Risk Ratio
RR 95%−CI
Weight (common)
Weight (random)
Yi 2023 65 85 533 920 1.32 [1.16; 1.50] 89.8% 21.8%
Komarowska 2020 1 1 4 8 1.89 [1.00; 3.56] 1.6% 0.9%
Yu 2021 5 5 215 294 1.37 [1.28; 1.46] 8.6% 77.3%
Common effect model 91 1222 1.32 [1.17; 1.49] 100.0% .
Random effects model 1.36 [1.28; 1.45] . 100.0%
Heterogeneity: I2 = 0.0%, τ2 < 0.0001, p = 0.5344
0.5
1
2
D
>12h, Abdominal pain
Abdominal pain
Without
Study
Events Total Events Total
Risk Ratio
RR 95%−CI
Weight (common)
Weight (random)
Sasa 2025 8 36 16 67 0.93 [0.44; 1.96] 14.4% 5.5%
Yi 2023 41 60 557 945 1.16 [0.97; 1.39] 85.6% 94.5%
Common effect model 96 1012 1.13 [0.94; 1.35] 100.0% .
Random effects model 1.15 [0.96; 1.36] . 100.0%
Heterogeneity: I2 = 0.0%, τ2 = 0, p = 0.5744
0.5
1
2
E
>12h, Hydrocele
Hydrocele
Without
Study
Events Total Events Total
Risk Ratio
RR 95%−CI
Weight (common)
Weight (random)
Yu 2021 103 154 117 147 0.84 [0.73; 0.96] 62.4% 50.9%
Afsarlar 2019 88 171 47 52 0.57 [0.48; 0.68] 37.6% 49.1%
Common effect model 325 199 0.74 [0.66; 0.82] 100.0% .
Random effects model 0.69 [0.47; 1.02] . 100.0%
Heterogeneity: I2 = 91.7%, τ2 = 0.0695, p = 0.0005
0.5
1
2
F
>12h, Manual detorsion
Manual detorsion
Without
Study
Events Total Events Total
Risk Ratio
RR 95%−CI
Weight (common)
Weight (random)
Sasa 2025 0 7 24 96 0.26 [0.02; 3.91] 3.4% 0.8%
Yi 2023 41 96 557 909 0.70 [0.55; 0.88] 96.6% 99.2%
Common effect model 103 1005 0.68 [0.54; 0.87] 100.0% .
Random effects model 0.69 [0.55; 0.88] . 100.0%
Heterogeneity: I2 = 0.0%, τ2 = 0, p = 0.4802
0.1
0.5
1
2
10
