# Supplementary material for: Are we still too late to preserve the testes? A global survey of delayed consultation and risk factors for testicular torsion: a systematic review and meta-analysis
Source: Front Reprod Health. 2026 Feb 24;8:1735652. doi: 10.3389/frph.2026.1735652 (PMC12971663; doi:10.3389/frph.2026.1735652)
Supplement: Supplementary file 11 [file Datasheet8.pdf]

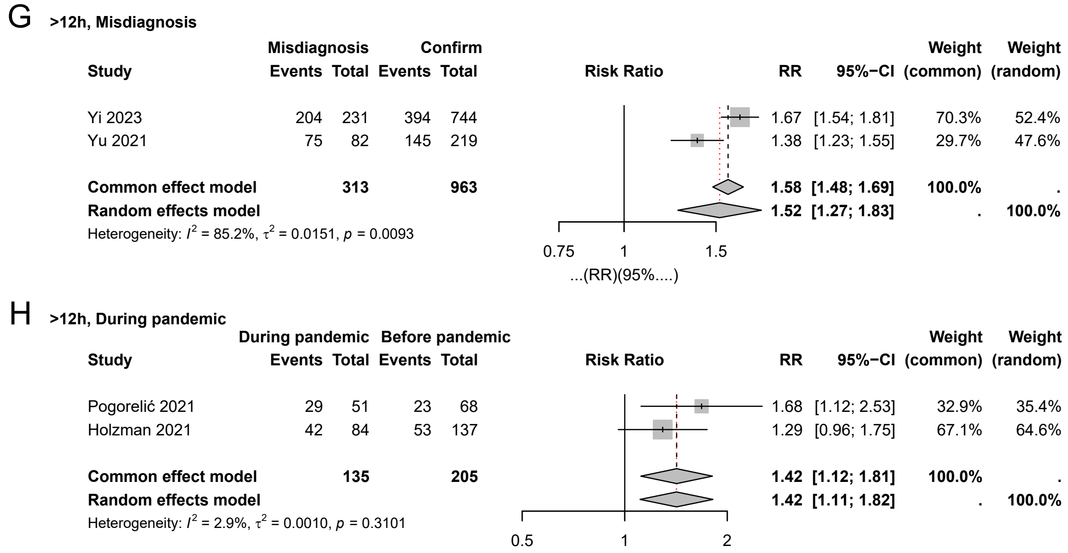
G
>12h, Misdiagnosis
Misdiagnosis
Confirm
Study
Events Total Events Total
Risk Ratio
RR
95%−CI
Weight (common)
Weight (random)
Yi 2023 204 231 394 744 1.67 [1.54; 1.81] 70.3% 52.4%
Yu 2021 75 82 145 219 1.38 [1.23; 1.55] 29.7% 47.6%
Common effect model 313 963 1.58 [1.48; 1.69] 100.0% .
Random effects model 1.52 [1.27; 1.83] . 100.0%
Heterogeneity: I^2 = 85.2%, τ^2 = 0.0151, p = 0.0093
0.75
1
1.5
...(RR)(95%....)
H
>12h, During pandemic
During pandemic
Before pandemic
Study
Events Total Events Total
Risk Ratio
RR
95%−CI
Weight (common)
Weight (random)
Pogorelić 2021 29 51 23 68 1.68 [1.12; 2.53] 32.9% 35.4%
Holzman 2021 42 84 53 137 1.29 [0.96; 1.75] 67.1% 64.6%
Common effect model 135 205 1.42 [1.12; 1.81] 100.0% .
Random effects model 1.42 [1.11; 1.82] . 100.0%
Heterogeneity: I^2 = 2.9%, τ^2 = 0.0010, p = 0.3101
0.5
1
2
