# Supplementary material for: Are we still too late to preserve the testes? A global survey of delayed consultation and risk factors for testicular torsion: a systematic review and meta-analysis
Source: Front Reprod Health. 2026 Feb 24;8:1735652. doi: 10.3389/frph.2026.1735652 (PMC12971663; doi:10.3389/frph.2026.1735652)
Supplement: Supplementary file 12 [file Datasheet9.pdf]

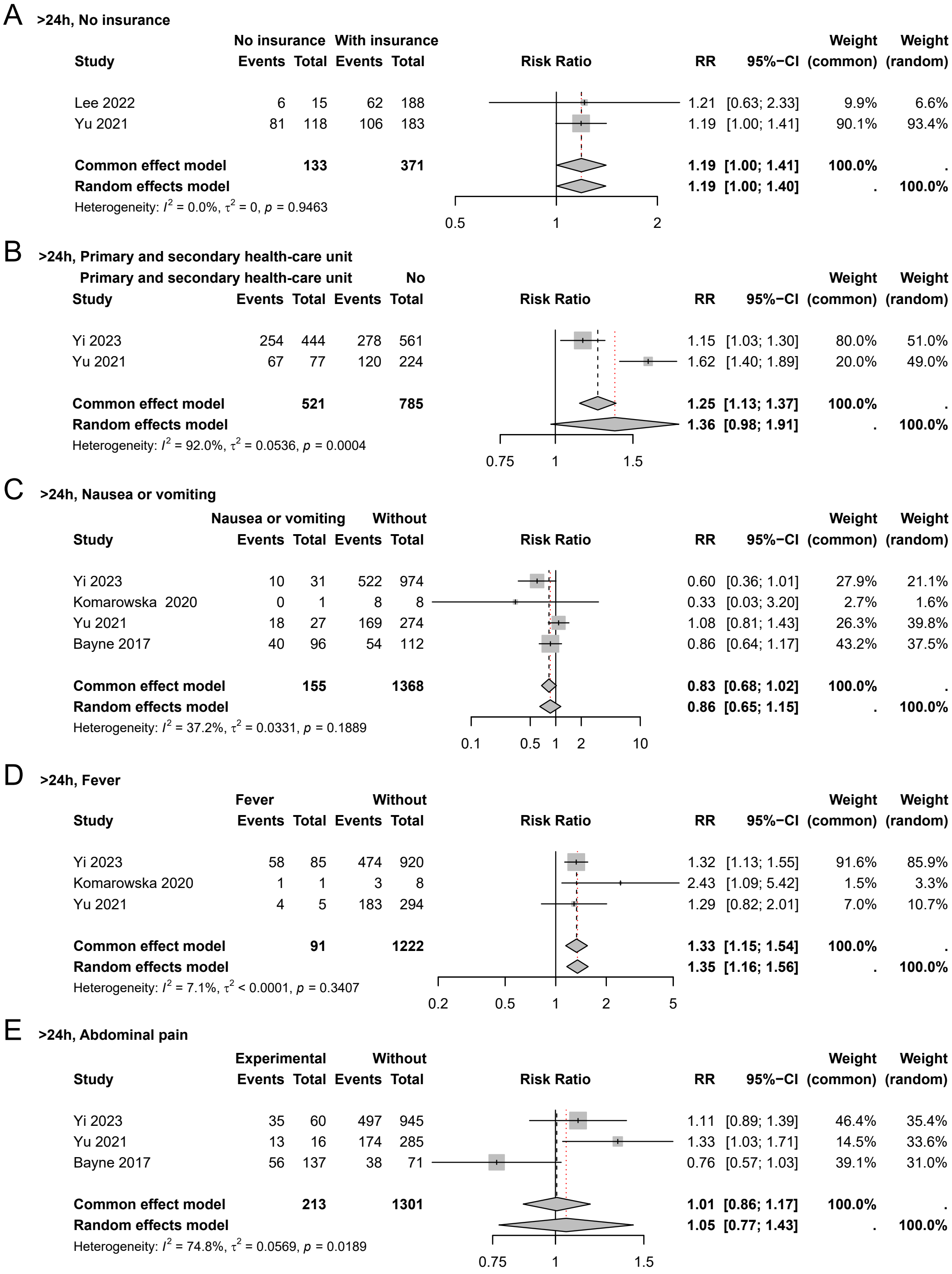
A
>24h, No insurance
No insurance With insurance Weight Weight
Study Events Total Events Total Risk Ratio RR 95%−CI (common) (random)
Lee 2022 6 15 62 188 1.21 [0.63; 2.33] 9.9% 6.6%
Yu 2021 81 118 106 183 1.19 [1.00; 1.41] 90.1% 93.4%
Common effect model 133 371 1.19 [1.00; 1.41] 100.0% .
Random effects model 1.19 [1.00; 1.40] . 100.0%
Heterogeneity: $I^2$ = 0.0%, $\tau^2$ = 0, $p$ = 0.9463
0.5 1 2
B
>24h, Primary and secondary health-care unit
Primary and secondary health-care unit No Weight Weight
Study Events Total Events Total Risk Ratio RR 95%−CI (common) (random)
Yi 2023 254 444 278 561 1.15 [1.03; 1.30] 80.0% 51.0%
Yu 2021 67 77 120 224 1.62 [1.40; 1.89] 20.0% 49.0%
Common effect model 521 785 1.25 [1.13; 1.37] 100.0% .
Random effects model 1.36 [0.98; 1.91] . 100.0%
Heterogeneity: $I^2$ = 92.0%, $\tau^2$ = 0.0536, $p$ = 0.0004
0.75 1 1.5
C
>24h, Nausea or vomiting
Nausea or vomiting Without Weight Weight
Study Events Total Events Total Risk Ratio RR 95%−CI (common) (random)
Yi 2023 10 31 522 974 0.60 [0.36; 1.01] 27.9% 21.1%
Komarowska 2020 0 1 8 8 0.33 [0.03; 3.20] 2.7% 1.6%
Yu 2021 18 27 169 274 1.08 [0.81; 1.43] 26.3% 39.8%
Bayne 2017 40 96 54 112 0.86 [0.64; 1.17] 43.2% 37.5%
Common effect model 155 1368 0.83 [0.68; 1.02] 100.0% .
Random effects model 0.86 [0.65; 1.15] . 100.0%
Heterogeneity: $I^2$ = 37.2%, $\tau^2$ = 0.0331, $p$ = 0.1889
0.1 0.5 1 2 10
D
>24h, Fever
Fever Without Weight Weight
Study Events Total Events Total Risk Ratio RR 95%−CI (common) (random)
Yi 2023 58 85 474 920 1.32 [1.13; 1.55] 91.6% 85.9%
Komarowska 2020 1 1 3 8 2.43 [1.09; 5.42] 1.5% 3.3%
Yu 2021 4 5 183 294 1.29 [0.82; 2.01] 7.0% 10.7%
Common effect model 91 1222 1.33 [1.15; 1.54] 100.0% .
Random effects model 1.35 [1.16; 1.56] . 100.0%
Heterogeneity: $I^2$ = 7.1%, $\tau^2$ < 0.0001, $p$ = 0.3407
0.2 0.5 1 2 5
E
>24h, Abdominal pain
Experimental Without Weight Weight
Study Events Total Events Total Risk Ratio RR 95%−CI (common) (random)
Yi 2023 35 60 497 945 1.11 [0.89; 1.39] 46.4% 35.4%
Yu 2021 13 16 174 285 1.33 [1.03; 1.71] 14.5% 33.6%
Bayne 2017 56 137 38 71 0.76 [0.57; 1.03] 39.1% 31.0%
Common effect model 213 1301 1.01 [0.86; 1.17] 100.0% .
Random effects model 1.05 [0.77; 1.43] . 100.0%
Heterogeneity: $I^2$ = 74.8%, $\tau^2$ = 0.0569, $p$ = 0.0189
0.75 1 1.5
