# Supplementary material for: Are we still too late to preserve the testes? A global survey of delayed consultation and risk factors for testicular torsion: a systematic review and meta-analysis
Source: Front Reprod Health. 2026 Feb 24;8:1735652. doi: 10.3389/frph.2026.1735652 (PMC12971663; doi:10.3389/frph.2026.1735652)
Supplement: Supplementary file 14 [file Datasheet11.pdf]

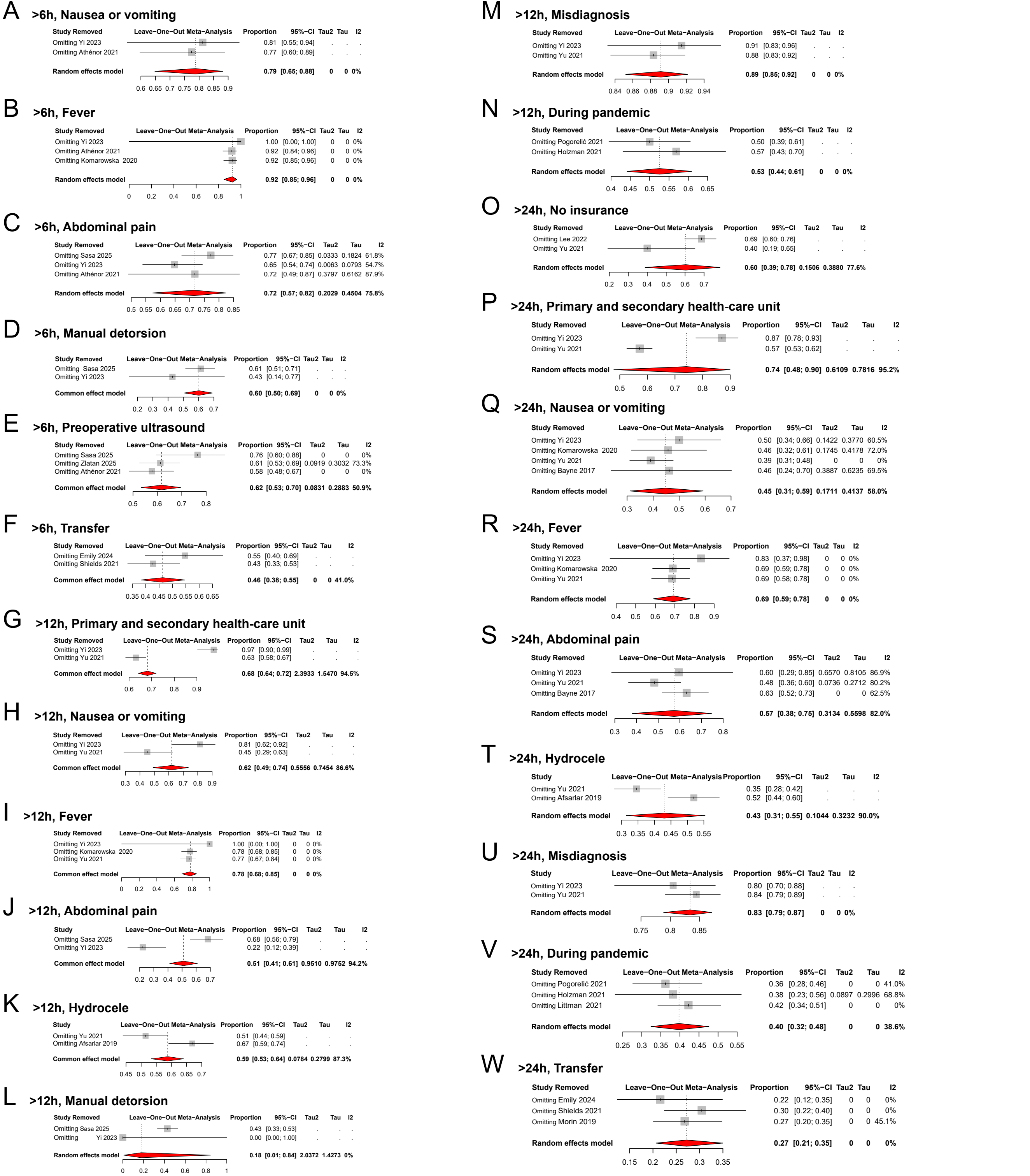
A >6h, Nausea or vomiting
Study Removed Leave-One-Out Meta-Analysis Proportion 95%-CI Tau2 Tau I2
Omitting Yi 2023 0.81 [0.55; 0.94] . . .
Omitting Athénor 2021 0.77 [0.60; 0.89] . . .
Random effects model 0.79 [0.65; 0.88] 0 0 0%
0.6 0.65 0.7 0.75 0.8 0.85 0.9
B >6h, Fever
Study Removed Leave-One-Out Meta-Analysis Proportion 95%-CI Tau2 Tau I2
Omitting Yi 2023 1.00 [0.00; 1.00] 0 0 0%
Omitting Athénor 2021 0.92 [0.84; 0.96] 0 0 0%
Omitting Komarowska 2020 0.92 [0.85; 0.96] 0 0 0%
Random effects model 0.92 [0.85; 0.96] 0 0 0%
0 0.2 0.4 0.6 0.8 1
C >6h, Abdominal pain
Study Removed Leave-One-Out Meta-Analysis Proportion 95%-CI Tau2 Tau I2
Omitting Sasa 2025 0.77 [0.67; 0.85] 0.0333 0.1824 61.8%
Omitting Yi 2023 0.65 [0.54; 0.74] 0.0063 0.0793 54.7%
Omitting Athénor 2021 0.72 [0.49; 0.87] 0.3797 0.6162 87.9%
Random effects model 0.72 [0.57; 0.82] 0.2029 0.4504 75.8%
0.5 0.55 0.6 0.65 0.7 0.75 0.8 0.85
D >6h, Manual detorsion
Study Removed Leave-One-Out Meta-Analysis Proportion 95%-CI Tau2 Tau I2
Omitting Sasa 2025 0.61 [0.51; 0.71] . . .
Omitting Yi 2023 0.43 [0.14; 0.77] . . .
Common effect model 0.60 [0.50; 0.69] 0 0 0%
0.2 0.3 0.4 0.5 0.6 0.7
E >6h, Preoperative ultrasound
Study Removed Leave-One-Out Meta-Analysis Proportion 95%-CI Tau2 Tau I2
Omitting Sasa 2025 0.76 [0.60; 0.88] 0 0 0%
Omitting Zlatan 2025 0.61 [0.53; 0.69] 0.0919 0.3032 73.3%
Omitting Athénor 2021 0.58 [0.48; 0.67] 0 0 0%
Common effect model 0.62 [0.53; 0.70] 0.0831 0.2883 50.9%
0.5 0.6 0.7 0.8
F >6h, Transfer
Study Removed Leave-One-Out Meta-Analysis Proportion 95%-CI Tau2 Tau I2
Omitting Emily 2024 0.55 [0.40; 0.69] . . .
Omitting Shields 2021 0.43 [0.33; 0.53] . . .
Common effect model 0.46 [0.38; 0.55] 0 0 41.0%
0.35 0.4 0.45 0.5 0.55 0.6 0.65
G >12h, Primary and secondary health-care unit
Study Removed Leave-One-Out Meta-Analysis Proportion 95%-CI Tau2 Tau I2
Omitting Yi 2023 0.97 [0.90; 0.99] . . .
Omitting Yu 2021 0.63 [0.58; 0.67] . . .
Common effect model 0.68 [0.64; 0.72] 2.3933 1.5470 94.5%
0.6 0.7 0.8 0.9
H >12h, Nausea or vomiting
Study Removed Leave-One-Out Meta-Analysis Proportion 95%-CI Tau2 Tau I2
Omitting Yi 2023 0.81 [0.62; 0.92] . . .
Omitting Yu 2021 0.45 [0.29; 0.63] . . .
Common effect model 0.62 [0.49; 0.74] 0.5556 0.7454 86.6%
0.3 0.4 0.5 0.6 0.7 0.8 0.9
I >12h, Fever
Study Removed Leave-One-Out Meta-Analysis Proportion 95%-CI Tau2 Tau I2
Omitting Yi 2023 1.00 [0.00; 1.00] 0 0 0%
Omitting Komarowska 2020 0.78 [0.68; 0.85] 0 0 0%
Omitting Yu 2021 0.77 [0.67; 0.84] 0 0 0%
Common effect model 0.78 [0.68; 0.85] 0 0 0%
0 0.2 0.4 0.6 0.8 1
J >12h, Abdominal pain
Study Leave-One-Out Meta-Analysis Proportion 95%-CI Tau2 Tau I2
Omitting Sasa 2025 0.68 [0.56; 0.79] . . .
Omitting Yi 2023 0.22 [0.12; 0.39] . . .
Common effect model 0.51 [0.41; 0.61] 0.9510 0.9752 94.2%
0.2 0.3 0.4 0.5 0.6 0.7
K >12h, Hydrocele
Study Leave-One-Out Meta-Analysis Proportion 95%-CI Tau2 Tau I2
Omitting Yu 2021 0.51 [0.44; 0.59] . . .
Omitting Afsarlar 2019 0.67 [0.59; 0.74] . . .
Common effect model 0.59 [0.53; 0.64] 0.0784 0.2799 87.3%
0.45 0.5 0.55 0.6 0.65 0.7
L >12h, Manual detorsion
Study Removed Leave-One-Out Meta-Analysis Proportion 95%-CI Tau2 Tau I2
Omitting Sasa 2025 0.43 [0.33; 0.53] . . .
Omitting Yi 2023 0.00 [0.00; 1.00] . . .
Random effects model 0.18 [0.01; 0.84] 2.0372 1.4273 0%
0 0.2 0.4 0.6 0.8 1
M >12h, Misdiagnosis
Study Removed Leave-One-Out Meta-Analysis Proportion 95%-CI Tau2 Tau I2
Omitting Yi 2023 0.91 [0.83; 0.96] . . .
Omitting Yu 2021 0.88 [0.83; 0.92] . . .
Random effects model 0.89 [0.85; 0.92] 0 0 0%
0.84 0.86 0.88 0.9 0.92 0.94
N >12h, During pandemic
Study Removed Leave-One-Out Meta-Analysis Proportion 95%-CI Tau2 Tau I2
Omitting Pogorelić 2021 0.50 [0.39; 0.61] . . .
Omitting Holzman 2021 0.57 [0.43; 0.70] . . .
Random effects model 0.53 [0.44; 0.61] 0 0 0%
0.4 0.45 0.5 0.55 0.6 0.65
O >24h, No insurance
Study Removed Leave-One-Out Meta-Analysis Proportion 95%-CI Tau2 Tau I2
Omitting Lee 2022 0.69 [0.60; 0.76] . . .
Omitting Yu 2021 0.40 [0.19; 0.65] . . .
Random effects model 0.60 [0.39; 0.78] 0.1506 0.3880 77.6%
0.2 0.3 0.4 0.5 0.6 0.7
P >24h, Primary and secondary health-care unit
Study Removed Leave-One-Out Meta-Analysis Proportion 95%-CI Tau2 Tau I2
Omitting Yi 2023 0.87 [0.78; 0.93] . . .
Omitting Yu 2021 0.57 [0.53; 0.62] . . .
Random effects model 0.74 [0.48; 0.90] 0.6109 0.7816 95.2%
0.5 0.6 0.7 0.8 0.9
Q >24h, Nausea or vomiting
Study Removed Leave-One-Out Meta-Analysis Proportion 95%-CI Tau2 Tau I2
Omitting Yi 2023 0.50 [0.34; 0.66] 0.1422 0.3770 60.5%
Omitting Komarowska 2020 0.46 [0.32; 0.61] 0.1745 0.4178 72.0%
Omitting Yu 2021 0.39 [0.31; 0.48] 0 0 0%
Omitting Bayne 2017 0.46 [0.24; 0.70] 0.3887 0.6235 69.5%
Random effects model 0.45 [0.31; 0.59] 0.1711 0.4137 58.0%
0.3 0.4 0.5 0.6 0.7
R >24h, Fever
Study Removed Leave-One-Out Meta-Analysis Proportion 95%-CI Tau2 Tau I2
Omitting Yi 2023 0.83 [0.37; 0.98] 0 0 0%
Omitting Komarowska 2020 0.69 [0.59; 0.78] 0 0 0%
Omitting Yu 2021 0.69 [0.58; 0.78] 0 0 0%
Random effects model 0.69 [0.59; 0.78] 0 0 0%
0.4 0.5 0.6 0.7 0.8 0.9
S >24h, Abdominal pain
Study Removed Leave-One-Out Meta-Analysis Proportion 95%-CI Tau2 Tau I2
Omitting Yi 2023 0.60 [0.29; 0.85] 0.6570 0.8105 86.9%
Omitting Yu 2021 0.48 [0.36; 0.60] 0.0736 0.2712 80.2%
Omitting Bayne 2017 0.63 [0.52; 0.73] 0 0 62.5%
Random effects model 0.57 [0.38; 0.75] 0.3134 0.5598 82.0%
0.3 0.4 0.5 0.6 0.7 0.8
T >24h, Hydrocele
Study Leave-One-Out Meta-Analysis Proportion 95%-CI Tau2 Tau I2
Omitting Yu 2021 0.35 [0.28; 0.42] . . .
Omitting Afsarlar 2019 0.52 [0.44; 0.60] . . .
Random effects model 0.43 [0.31; 0.55] 0.1044 0.3232 90.0%
0.3 0.35 0.4 0.45 0.5 0.55
U >24h, Misdiagnosis
Study Leave-One-Out Meta-Analysis Proportion 95%-CI Tau2 Tau I2
Omitting Yi 2023 0.80 [0.70; 0.88] . . .
Omitting Yu 2021 0.84 [0.79; 0.89] . . .
Random effects model 0.83 [0.79; 0.87] 0 0 0%
0.75 0.8 0.85
V >24h, During pandemic
Study Removed Leave-One-Out Meta-Analysis Proportion 95%-CI Tau2 Tau I2
Omitting Pogorelić 2021 0.36 [0.28; 0.46] 0 0 41.0%
Omitting Holzman 2021 0.38 [0.23; 0.56] 0.0897 0.2996 68.8%
Omitting Littman 2021 0.42 [0.34; 0.51] 0 0 0%
Random effects model 0.40 [0.32; 0.48] 0 0 38.6%
0.25 0.3 0.35 0.4 0.45 0.5 0.55
W >24h, Transfer
Study Removed Leave-One-Out Meta-Analysis Proportion 95%-CI Tau2 Tau I2
Omitting Emily 2024 0.22 [0.12; 0.35] 0 0 0%
Omitting Shields 2021 0.30 [0.22; 0.40] 0 0 0%
Omitting Morin 2019 0.27 [0.20; 0.35] 0 0 45.1%
Random effects model 0.27 [0.21; 0.35] 0 0 0%
0.15 0.2 0.25 0.3 0.35
